# CRISPR-Assisted Multiplex Base Editing System in *Pseudomonas putida* KT2440

**DOI:** 10.3389/fbioe.2020.00905

**Published:** 2020-07-31

**Authors:** Jun Sun, Li-Bing Lu, Tian-Xin Liang, Li-Rong Yang, Jian-Ping Wu

**Affiliations:** Institute of Bioengineering, College of Chemical and Biological Engineering, Zhejiang University, Hangzhou, China

**Keywords:** *Pseudomonas putida* KT2440, cytidine deaminase, base editing, gene inactivation, multiplex genome editing, Cas9 nickase

## Abstract

*Pseudomonas putida* (*P. putida*) KT2440 is a paradigmatic environmental-bacterium that possesses significant potential in synthetic biology, metabolic engineering and biodegradation applications. However, most genome editing methods of *P. putida KT2440* depend on heterologous repair proteins and the provision of donor DNA templates, which is laborious and inefficient. In this report, an efficient cytosine base editing system was established by using cytidine deaminase (APOBEC1), enhanced specificity Cas9 nickase (eSpCas9pp^D10A^) and the uracil DNA glycosylase inhibitor (UGI). This constructed base editor converts C-G into T-A in the absence of DNA strands breaks and donor DNA templates. By introducing a premature stop codon in target spacers, we successfully applied this system for gene inactivation with an efficiency of 25–100% in various *Pseudomonas* species, including *P. putida* KT2440, *P. aeruginosa* PAO1, *P. fluorescens* Pf-5 and *P. entomophila* L48. We engineered an eSpCas9pp^D10A^-NG variant with a NG protospacer adjacent motif to expand base editing candidate sites. By modifying the APOBEC1 domain, we successfully narrowed the editable window to increase gene inactivation efficiency in cytidine-rich spacers. Additionally, multiplex base editing in double and triple loci was achieved with mutation efficiencies of 90–100% and 25–35%, respectively. Taken together, the establishment of a fast, convenient and universal base editing system will accelerate the pace of future research undertaken with *P. putida* KT2440 and other *Pseudomonas* species.

## Introduction

*Pseudomonas* spp. are well-known gram-negative environmental bacteria, which contain more than 200 species (Nikel et al., 2014). *Pseudomonas* spp. can inhabit in a large diversity of niches, including the surface of plants, rhizosphere, insects and even humans (Silby et al., 2011). The strong environmental adaptability and great metabolic versatility of *Pseudomonas* spp. not only contribute to its survival under harsh conditions, but also attract more research into the field (Stover et al., 2000; Moreno and Rojo, 2014). The research area of *Pseudomonas* spp. can be divided mainly into three fields: Non-pathogenic *Pseudomonas putida* (*P. putida*) KT2440 is used as a chassis for synthetic biology, metabolic engineering and biocatalysis (Poblete-Castro et al., 2012); the opportunistic pathogen *P. aeruginosa* is regarded as a model strain for investigation of antibiotics resistance and disinfectants (Stover et al., 2000); the plant commensal *P. fluorescens* is well known for its biological control properties (Paulsen et al., 2005).

Targeted genome editing is an essential approach to exploit the physiological character of bacteria and in metabolic engineering and synthetic biology applications. The invention of counter-selection markers (*sacB* and *upp*) (Quénée et al., 2005; Graf and Altenbuchner, 2011) and heterologous recombinases (Flp and Cre) (Hoang et al., 1998; Luo et al., 2016) have increased the application of allelic exchange methods in *Pseudomonas* spp. Bacteriophage-based recombination proteins (λ-Red, Red/ET and Ssr) (Wenzel et al., 2005; Liang and Liu, 2010) are used to enhance the recombination efficiencies in *Pseudomonas* spp. The I-SceI homing endonuclease based system is a marker-free genome editing approach (Martínez-García and de Lorenzo, 2011) that has been used to delete a large DNA fragment in *P. putida* KT2440. However, genome editing using these approaches is inefficient and manipulation is time-consuming and tedious. In recent years, Cas9 assisted genome editing systems have revolutionarily accelerated the development of genetic studies in different organisms (Jakoèiunas et al., 2016), including *Pseudomonas* spp. (Aparicio et al., 2018; Sun et al., 2018; Wu et al., 2019a). Nevertheless, the developed *P. putida* CRISPR/Cas9 systems require the introduction of a heterologous recombination system because of inefficient homology-directed repair (HDR), and the provision of donor DNA templates or single-stranded DNA is also not straightforward (Aparicio et al., 2018; Sun et al., 2018). Additionally, the superior characteristics of the CRISPR/Cas9 system in multiplex genome editing has only been achieved in two loci with extremely low efficiency (Aparicio et al., 2018).

The CRISPR-assisted cytidine deaminase system is a newly developed base editing approach that uses cytidine deaminase (APOBEC1 or AID) with a catalytically impaired Cas9 to enable C:G to T:A mutations (Komor et al., 2016; Nishida et al., 2016). In the absence of double-stranded DNA breaks (DSBs) or donor DNA templates, this base editing system can directly target single-stranded DNA because Cas9 binding facilitates the recruitment of cytidine deaminases (Komor et al., 2016). By targeting CGA (Arg), CAG (Gln), and CAA (Gln) in the coding strand or ACC (Trp) in the non-coding strand, the cytidine deaminase base editor introduces a premature stop codon for gene inactivation (Wang Y. et al., 2018). However, the efficiency of cytosine base editing *in vivo* is potentially affected by the endogenous base excision repair enzyme uracil *N*-glycosylase (UNG) (Rees and Liu, 2018). To circumvent reversal of this base conversion by UNG, a bacteriophage derived uracil DNA glycosylase inhibitor (UGI) is usually fused to the C-terminus of the APOBEC1-nCas9^D10A^ complex to inhibit UNG and improve base editing efficiencies. Currently, cytidine deaminase-based base editing system have been extended to *Escherichia coli* (Nishida et al., 2016), *Clostridium beijerinckii* (Li et al., 2019), *Klebsiella pneumonia* (Yu et al., 2018), *Corynebacterium glutamicum* (Wang et al., 2019), and some *Pseudomonas* spp. (Chen et al., 2018). However, this *Pseudomonas* cytosine base editing system is highly dependent on a NGG protospacer-adjacent motif (PAM). The potential editable window is located between position −18 and −13 upstream of the PAM sequence, and base editing efficiencies are influenced by nucleotides neighboring C bases and the size of the editable window (Komor et al., 2016; Chen et al., 2018).

**FIGURE 1:**
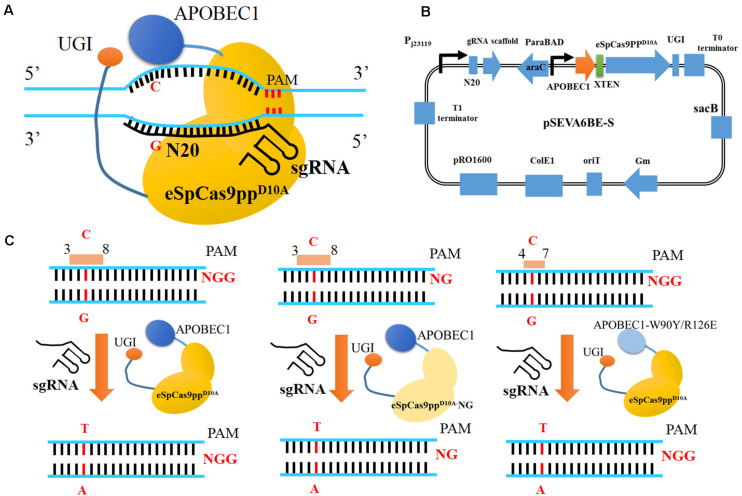
CRISPR-Assisted cytosine base editing in *Pseudomonas putida* KT2440. **(A)** Schematic representation of CRISRP/Cas9 system-assisted base editing. **(B)** The plasmid containing APOBEC1, eSpCas9pp^D10A^, sgRNA, UGI and *sacB* was named as pSEVA6BE-S. **(C)** Three strategies for base editing in target nucleotides (cytidines). APOBEC1, rat cytosine deaminase; N20, the 20-bp sequence at the 5′ end upstream of PAM; PAM, protospacer adjacent motif; UGI, uracil DNA glycosylase inhibitor.

In this study, we have established a CRISPR-assisted templates-free base editing system (pSEVA6BE) in *P. putida* KT2440 (Figure 1A). This system (Figure 1B) comprises an enhanced specificity Cas9 nickase variant [eSpCas9pp^D10A^ containing the mutations K848A/K1003A/R1060A (Slaymaker et al., 2016)], a cytidine deaminase (APOBEC1) and a uracil DNA glycosylase inhibitor (UGI), and converted specific C nucleotides to T. By introducing a premature stop codon, this base editor was used for gene inactivation in *P. putida* KT2440 with maximum efficiencies of 100%. The cytosine base editing system was successfully extended into *P. aeruginosa* PAO1, *P. fluorescens* Pf-5 and *P. entomophila* L48. To expand the base editing scope, we introduced seven mutations [L1111R/D1135V/G1218R/E1219F/A1322R/R1335A/T1337R (Endo et al., 2019)] into eSpCas9pp^D10A^ to modify PAM specificities from NGG to NG (Figure 1C). To obtain precise base editing in cytidine-rich spacers, we engineered an APOBEC1 variant [mutations W90Y and R126E (Kim et al., 2017)] with a narrow editing window (Figure 1C). Multiplex base editing of double-locus and triple-locus in *P. putida* KT2440 was proved effective by one-plasmid and two-plasmid systems. By utilizing the base editor, gene knockout and amino acid substitution were implemented in *P. putida* KT2440 for enhancing the production of protocatechuic acid.

## Materials and Methods

### Strains, Growth Conditions and Reagent

All of the strains used in this study are listed in Supplementary Table S1. *Escherichia coli* DH5α was used for cloning and maintenance. Luria Broth (LB) medium was used for cell growth. King’s medium (18 g/L Glycerol, 20 g/L Tryptone, 1.498 g/L MgSO_4_.7H_2_O and 0.673 g/L K_2_HPO_4_.3H_2_O) was used for shaking flask fermentation. *P. putida* KT2440, *P. entomophila* L48 and *P. fluorescens* Pf-5 were grown at 30°C, while *P. aeruginosa* PAO1 and *Escherichia coli* were cultivated at 37°C. Antibiotics were added as the following concentrations: gentamicin (Gm), 50 μg/mL (*E. coli*), 100 μg/mL (*P. putida* KT2440, *P. entomophila* L48, and *P. protegens* Pf-5) and 30 μg/mL (*P. aeruginosa* PAO1); kanamycin (Km), 50 μg/mL (*E. coli*) and 100 μg/mL (*P. putida* KT2440).

Phanta Max Super-Fidelity DNA Polymerase was used for DNA amplification, and Green Taq mix was applied to colony PCR. The One Step Cloning Kit was used for seamless cloning. All of these reagents were purchased from Vazyme Biotech Co., Ltd (Nanjing, China).

### Plasmid Construction

All of the plasmids and primers used in this study are listed in Supplementary Tables S2, S3. The genes eSpCas9pp [enhanced specificity Cas9 containing three mutations K848A/K1003A/R1060A (Slaymaker et al., 2016)] and APOBEC1 [cytidine deaminase (Komor et al., 2016)] were synthesized by Genscript (Nanjing, China) with codon optimized according to the codon preference of *Pseudomonas putida* KT2440. Gene SpCas9 was amplified from pCAS-RK2T (Sun et al., 2018) using primers S9-F/R. Uracil DNA glycosylase inhibitor (UGI) was cloned from pCMV-BE3 using primers U-F/R. To construct Cas9 nickase variants, the mutation of D10A in eSpCas9pp and SpCas9 was introduced by point mutations using primers eC9D10A-F/R and C9D10A-F/R, respectively. The NG PAM recognizing eSpCas9pp^D10A^-NG was obtained by introducing seven mutations [L1111R, D1135V, G1218R, E1219F, A1322R, R1335A, and T1337R (Endo et al., 2019)] into eSpCas9pp^D10A^ using primers 11-F/R, 12-F/R, and 13-F/R. The variant APOBEC1-YE1 with a narrow editing window was obtained by site-directed mutagenesis at W90Y and R126E (Kim et al., 2017) using primers YE-1F/R.

Plasmid pSEVA-gRNAF (Sun et al., 2018) was used as template for plasmid construction. pSEVA-TtgA was modified from pSEVA-gRNAF, which was obtained by introducing a TtgA spacer. Here, we constructed 5 expression modules for cytidine deaminase-mediated base editing on the basis of pSEVA-TtgA. Among these five modules, XTEN linker (Komor et al., 2016) was used to link APOBEC1 to the N-terminus of SpCas9^D10A^ or eSpCas9pp^D10A^. The cassettes APOBEC1-XTEN-SpCas9^D10A^ and APOBEC1-XTEN-eSpCas9pp^D10A^ under the control of the constitutive promoter Pbs (Yang et al., 2018) were inserted into pSEVA-TtgA, which generates pSEVA-Module 1 and pSEVA-Module 3, respectively. Inducible promoters Xyls-Pm and AraC-ParaBAD were amplified from pSEVA258 and pCAS-RK2T, respectively. The constitutive promoter Pbs in pSEVA-Module 1 and pSEVA-Module 3 was replaced by AraC-ParaBAD, generating pSEVA-Module 2 and pSEVA-Module 4, respectively. pSEVA-Module 5 was constructed by substituting Xyls-Pm for AraC-ParaBAD in pSEVA-Module 4. The element UGI was fused to the C-terminus of APOBEC1-XTEN- eSpCas9pp^D10A^ in pSEVA-Module 4, giving rise to pSEVA-Module 6. pSEVA-Module 6 was named pSEVA6BE, which was used as the template for the construction of following base editing plasmids (Supplementary Table S2). All of the base editing spacers (Supplementary Table S4) used in this study were designed and analyzed by CasOT (Xiao et al., 2014), gBIG (Wang et al., 2019), or BE-Designer (Hwang et al., 2018).

By replacing eSpCas9pp^D10A^ with eSpCas9pp^D10A^-NG, a PAM-altering plasmid pSEVA6BE-NG was derived from pSEVA6BE using primers eC9NG-1F/1R and eC9NG-2F/2R. In a similar way, plasmid pSEVA6BE-YE1 was obtained by the substitution of APOBEC1-YE1 for APOBEC1 (using primers BE-1F/1R and BE-2F/2R) in pSEVA6BE. By the combination of the backbone T1 terminator-sgRNA cassette-cytosine base editor Module 6-T0 terminator from pSEVA6BE and the fragment RSF1010 replicon-kanamycin resistance marker from pVLT33 using primers 62-1F/1R and 62-2F/2R, pSEVA2BE was constructed by seamless cloning.

The sequence of counter-selection marker *sacB* was cloned from pCAS-RK2T, and inserted into the plasmids pSEVA2BE, pSEVA6BE-YE1, pSEVA6BE-NG and pSEVA6BE via seamless cloning using primers Sac-1F/1R and Sac-2F/2R, which generated pSEVA2BE-S, pSEVA6BE-YE1-S, pSEVA6BE-NG-S, and pSEVA6BE-S, respectively.

### Base Editing in *Pseudomonas*

pSEVA6BE, pSEVA6BE-NG, pSEVA6BE-YE1 or other derivative plasmids was transformed into *Pseudomonas* according to a previous electroporation method (Sun et al., 2018). After electroporation, the cells were recovered at 30°C (*P. aeruginosa* PAO1 at 37°C) for 2 h. Next, the recovered cells were concentrated and then plated onto LB agar containing antibiotics to select recombinants. The individual transformant was inoculated into a 5 mL LB tube together with the addition of antibiotics and inducers (6 mg/mL L-arabinose or 2 mg/mL *m*-toluic acid) and then cultivated at 30°C (*P. aeruginosa* PAO1 at 37°C) for 24 h. After the cultivation process, the base editing cells were streaked onto the selective plates and cultured until the appearance of visible colonies. Colony PCR was used to amplify the target PCR products and base editing results were confirmed by DNA sequencing.

In particular, pSEVA6BE-PobA (or pSEVA6BE-PobA-TrpE) and pSEVA2BE-QuiC were co-transformed into *P. putida* KT2440 by electroporation for achieving multiplex base editing. In the coelectroporation recovery process, the cultivation time was extended to 3 h. To enhance the editing efficiency in multiple loci by one-plasmid or two-plasmid system, individual transformants colonies were cultured for 36 h.

### Plasmid Curing

To facilitate the loss of sacB-containing plasmids in cells, the mutant *Pseudomonas* strains were inoculated into a 5 mL LB tube containing 10 g/L sucrose and 5 g/L glucose, and then cultivated at the optimum growth temperature for 24 h. Next, the cultivated cells were streaked onto LB agar and plasmid-curing was identified by colony PCR using primers C9-F/R.

### Analytical Methods

Protocatechuic acid (PCA) was purchased from Aladdin Chemistry (Shanghai, China) and used as standards. The edited *P. putida* KT2440 strains were inoculated into LB medium without adding antibiotics. The cultivated strains were used as seed cultures and then transferred into 250 mL flasks containing 50 mL King’s medium under agitation rate of 250 rpm at 30°C for 66 h. The titer of PCA in *P. putida* KT2440 fermentation cultures was analyzed by HPLC (SHIMADZU, Prominence LC-20A) with a reverse phase C18 column (Pntulips^TM^ QS-C18, 5 μm, 250 × 4.6 mm). The flowing phase comprised 70% solvent A (water with 0.1% formic acid) and 30% solvent B (methanol). The optimal ultraviolet absorbance for PCA was set to 270 nm. The temperature of column oven was controlled at 40°C and the flowing rate was adjusted to 0.5 mL/min.

## Results

### Establishment of a Cytosine Base Editing System in *P. putida* KT2440

**FIGURE 2:**
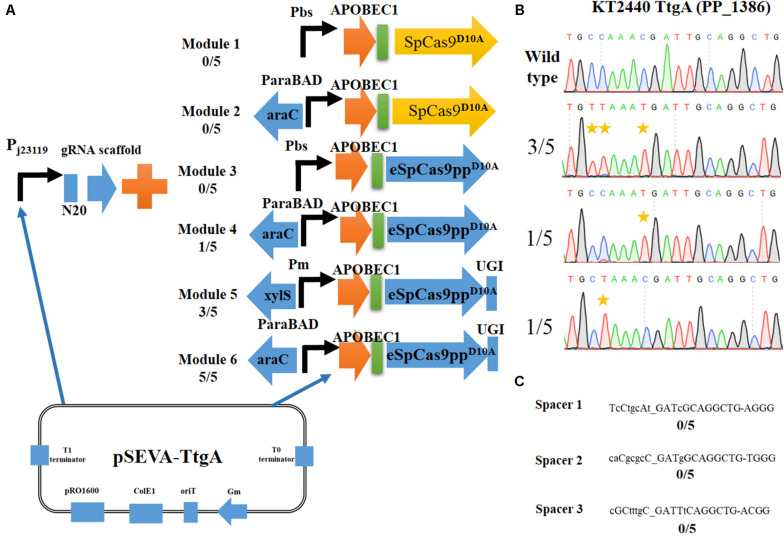
Construction of an efficient cytosine base editing system in *Pseudomonas putida* KT2440. **(A)** Six different modules for cytosine base editing designed in this study. **(B)** Determination of pSEVA-Module 6 (pSEVA6BE) mediated cytosine base editing in the TtgA spacer region. **(C)** Potential off-target loci of TtgA in *P. putida* KT2440 genome.

Six expression combinations (Figure 2A) of APOBEC1 and Cas9^D10A^ nickase (a codon non-optimized SpCas9^D10A^ or a codon-optimized and enhanced specificity eSpCas9pp^D10A^) were evaluated to establish an efficient cytidine base editing system in *P. putida* KT2440. Using the plasmid pSEVA-gRNAF (Sun et al., 2018) as the template, a N20 sequence (TtgA spacer targeting the *ttgA* gene in KT2440) was used as the target site and inserted into pSEVA-gRNAF to give pSEVA-TtgA.

Initially, we constructed two different Cas9^D10A^ nickase expression modules, Module 1 (containing SpCas9^D10A^) and Module 3 (containing eSpCas9pp^D10A^). Both of these modules were under the control of the strong constitutive promoter Pbs (Yang et al., 2018). Performance of the base editing process revealed that none of the ten selected colonies achieved cytidine mutations in the target-editing window. The constitutive promoter Pbs was exchanged with the inducible arabinose promoter, AraC-ParaBAD to generate Modules 2 and Modules 4. Modules 4 converted C to T successfully in one of the five colonies, whereas no base editing was detected for Module 2. Thus, cytosine base editing was achieved by overexpression of Module 4; however, the mutation efficiency was low. We hypothesized that cellular cytidine base editing could be reversed by base excision repair, which reduces the base editing efficiency. To verify our hypothesis, the uracil DNA glycosylase inhibitor (UGI) was fused to the C-terminus of APOBEC1-eSpCas9pp^D10A^to yield the cassette APOBEC1-eSpCas9pp^D10A^-UGI. Two common inducible promoters Xyls-Pm and AraC-ParaBAD, were introduced to control this cassette to give Modules 5 and 6. After the base editing procedure, DNA sequencing showed that three C-to-T mutants were identified from five random colonies harboring Module 5, and all five randomly selected strains containing Modules 6 converted C to T, which indicates that Module 6 exhibited a higher efficiency than Module 5. To verify the effects of adding UGI, we designed another two spacers GllA and MexE as target sites by using pSEVA-Module 4 and pSEVA-Module 6. DNA sequencing results (Supplementary Figures S1, S2) showed that the mutation rate of cytosine to guanine for spacers GllA and MexE increased from 60 and 40% in Module 4 to 100 and 80% in Module 6, which proves that the addition of UGI improved the editing efficiency.

DNA sequencing results of colonies containing Module 6 detected three different cytidine substitutions (Figure 2B). Among the five C→T mutations, 3/5 base editing substitution occurred at positions 3 and 4, 1/5 occurred at position 8 and 1/5 at position 4, with the PAM sequence at position 21–23. The editing efficiency of cytidines (TC ≥ CC ≥ AC > GC) was in consistent with previous reports in mammalian cells (Komor et al., 2016). Mutations at potential off-target loci by the Module 6-containing base editing system were evaluated. With the help of CasOT (Xiao et al., 2014), three top-similar DNA sequences (Figure 2C) of this *ttgA* N20 sequence were identified in the *P. putida* KT2440 genome and DNA sequencing of these spacer sites showed that none of them had point mutations (Supplementary Figure S3). These results confirmed that the Module 6-containing cytosine base editing system exhibited high specificity. Thus, Module 6 was identified as the optimal expression cassette. The plasmid containing Module 6 was named pSEVA6BE and used as the backbone for the following base editing experiments.

### Application of the Cytosine Base Editing System for Gene Inactivation in *P. putida* KT2440

Having demonstrated the feasibility of pSEVA6BE for cytosine base editing in *P. putida* KT2440, we sought to apply this system for gene inactivation by introducing a premature stop codon. Because of its capability to convert codons CAA, CAG, CGA, and TGG into a stop codon, the cytidine base editing system can be used to inactivate genes without generating DSBs and the provision of DNA repairing templates (Arazoe et al., 2018). To assess the efficiencies of gene inactivation, we selected *hmgA*, *pobA*, *quiC*, and *ttgA* as target sites.

**FIGURE 3:**
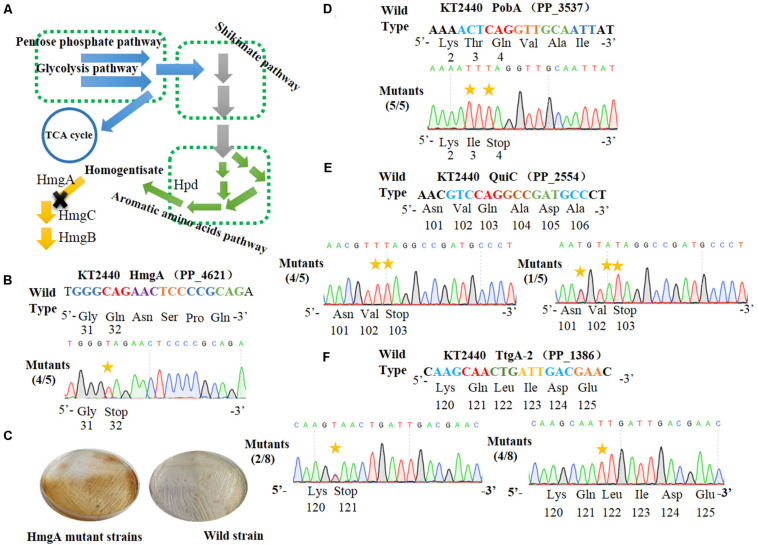
Cytosine base editor-mediated gene inactivation in *Pseudomonas putida* KT2440. **(A)** Overview of the homogentisate pathway in *P. putida* KT2440. **(B)** Mutation alignment of the targeted HmgA spacer. **(C)** Phenotypes of *hmgA* knockout mutants. The *hmgA* mutant strains of *P. putida* KT2440 were streaked on selective plates. **(D)** The PobA spacer in *pobA* gene was selected for base editing to achieve gene inactivation. **(E)** Base editing results of Quic spacer in *quiC* gene. **(F)** Sequence alignment of the TtgA mutants after cytosine base editing.

*HmgA*, encoding homogentisate dioxygenase, is a key gene of the homogentisate pathway (Figure 3A) in *P. putida* KT2440 (Arias-Barrau et al., 2004). The deletion of *hmgA* can disable the ring-cleavage reaction of homogentisate, which leads to the accumulation of homogentisate. As homogentisate can be oxidized into dark brown by oxygen, we used *hmgA* as a reporter to assess the efficiency of gene inactivation in *P. putida* KT2440. After the plasmid pSEVA6BE-HmgA targeting *hmgA* was introduced into *P. putida* KT2440, the transformant colonies were inoculated, cultivated and streaked on selective LB agar plates. As shown in Figure 3C, mutant cells produced the dark brown pigment when compared with that of the control wild-type strain. Five random mutant colonies were selected for colony PCR and DNA sequencing. The sequencing results (Figure 3B) showed that the codon CAG representing residue Gln32 was successfully mutated to TAG in four out of five colonies, thus disabling the activity of HmgA. In the case of PobA, the N20 sequence harbors two potential editable cytidines (Cs) at positions 5 and 7. To achieve gene inactivation of *pobA*, substitution of C→T should occur at position 7, or positions 5 and 7. DNA sequencing of the base editing results (Figure 3D) showed that all five randomly picked colonies carried the C to T mutation, yielding an editing efficiency for *pobA* of 100%. The N20 sequence from *quiC* contains three editable Cs at positions of 3, 6, and 7. Base editing results (Figure 3E) showed that all five identified strains had successfully performed gene inactivation by introduction of a stop codon at amino acid position 103. Among these mutants, 80% of the strains had the C→T substitution at positions of 6 and 7, and the remaining 20% harbored two C→T mutations (at positions 3 and 7) and one C→A mutation (at position 6) within the base editing window. Next, the plasmid pSEVA6BE-TtgA-2 was transformed into *P. putida* KT2440 to test the editing efficiency of the TtgA-2 spacer. The possible editable Cs in the TtgA-2 spacer are located at positions of 5 and 8. The results of colony PCR (Figure 3F) showed that half of the eight selected strains exhibited the C→T substitution at position 8, and only 25% of the strains were identified to achieve gene inactivation by mutating CAA to TAA at position 5. The high mutation efficiencies in *hmgA*, *pobA*, and *quiC* demonstrated that the cytosine base editing system is an efficient tool for gene inactivation in *P. putida* KT2440. The editing results of TtgA-2 revealed the discrepancy of base editing toward cytosines with different adjacent nucleotides, which indicates that the AC motif had a higher base editing efficiency than GC. This is in agreement with the previously reported mutation preference of APOBEC1 (Chen et al., 2018).

### Cytosine Base Editing in *P. aeruginosa* PAO1, *P. fluorescens* Pf-5 and *P. entomophila* L48

**FIGURE 4:**
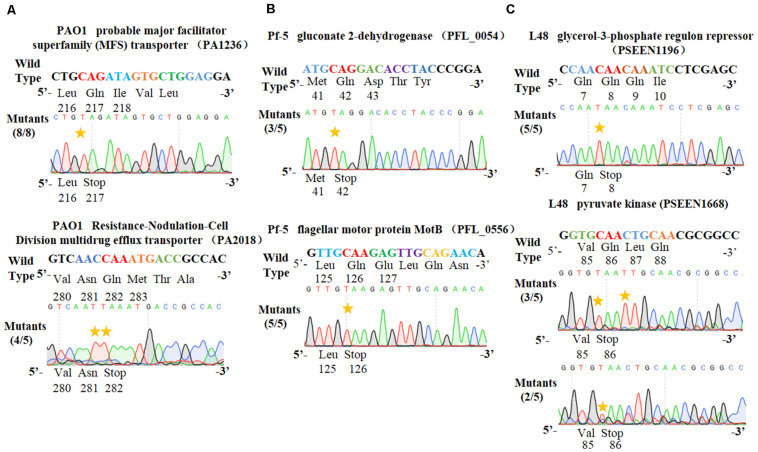
Application of the PSEVA6BE system into *Pseudomonas aeruginosa* PAO1, *Pseudomonas fluorescens* Pf-5 and *Pseudomonas entomophila* L48. **(A)** Examples of gene activation in *P*. *aeruginosa* PAO1 by cytosine base editor. **(B)** Results of cytosine base editing experiments in *P*. *fluorescens* Pf-5. **(C)** Examination of pSEVA6BE-mediated gene knockout in *P. entomophila* L48.

To test the universality of our cytosine base editor in *Pseudomonas* species, we tried to extend the pSEVA6BE system into *P. aeruginosa* PAO1, *P. fluorescens* Pf-5 and *P. entomophila* L48. *PA1236* (encoding probable major facilitator superfamily transporter) and *PA2018* (encoding resistance-nodulation-cell division multidrug efflux transporter) were selected as target sites for investigation of knock-out efficiency in *P. aeruginosa* PAO1. After base editing, DNA sequencing (Figure 4A) of PCR products showed that TAG and TAA could be generated with efficiencies of 100% and 80%, respectively. In *P. fluorescens* Pf-5, *PFL_0054* (encoding gluconate 2-dehydrogenase) and *PFL_0556* (encoding flagellar motor protein MotB) were designed as target base editing sites, respectively. As shown in Figure 4B, the codons CAG (Gln) and CAA (Gln) could be modified to the stop codons TAG and TAA with efficiencies of 3/5 and 5/5, respectively. Plasmids pSEVA6BE-L48glpR and pSEVA6BE-L48pyrF were designed to target the *PSEEN1196* and *PSEEN1668* genes in *P. entomophila* L48, respectively. Base editing results (Figure 4C) showed that C→T substitution could be achieved at position 5 in spacers PSEEN1196 and PSEEN1668 which resulted in knockout of these two genes. The application of the cytosine base editor in these three *Pseudomonas* species demonstrated the pSEVA6BE system can be a convenient and highly efficient genome editing tool in a wide range of *Pseudomonas* species.

### Expansion of the Base Editing Candidate Sites by Modification of PAM Specificities

**FIGURE 5:**
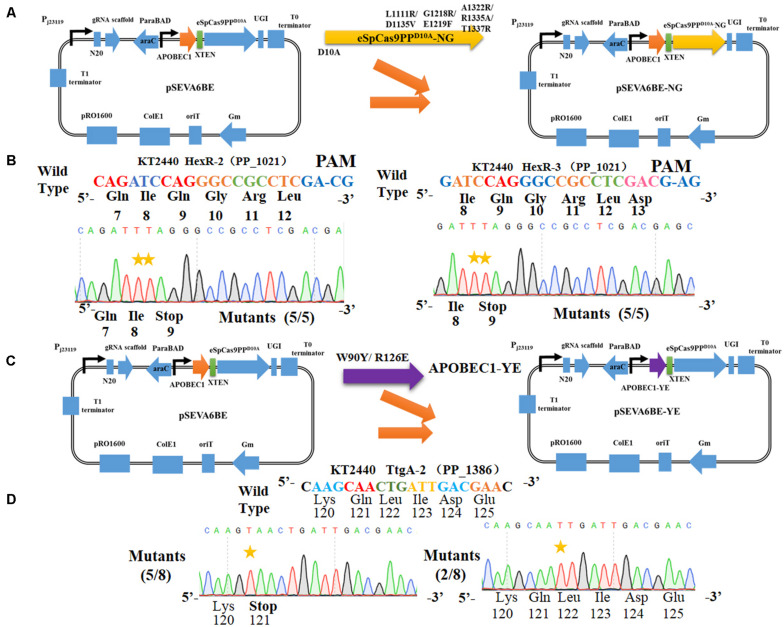
Altering the PAM specificity of eSpCas9PP^D10A^ and narrowing the editable window of APOBEC1. **(A)** The eSpCas9pp^D10A^–NG gene was cloned into pSEVA6BE, generating pSEVA6BE-NG. **(B)** C-to-T substitution was successfully achieved in target spacers HexR2 and HexR3 with NG PAM sequences. **(C)** The APOBEC1 gene in pSEVA6BE was replaced by the APOBEC1-YE1 gene, thus creating pSEVA6BE-YE. **(D)** pSEVA6BE-YE1-mediated base editing of TtgA-2 spacer tend to have a narrow editing windows compared to using pSEVA6BE (Figure 3F).

The cytosine base editor pSEVA6BE requires a strict PAM motif (SpCas9 recognizing NGG) and the target nucleotides (cytidines) must be distributed within the editable window. To expand the cytosine base editing candidate sites, the plasmid pSEVA6BE-NG containing an eSpCas9pp^D10A^-NG was constructed, which is capable of recognizing NG PAM. To that end the mutations [L1111R/D1135V/G1218R/E1219F/A1322R/R1335A/T1337R (Endo et al., 2019)] were introduced into the eSpCas9pp^D10A^ of pSEVA6BE (Figure 5A). To assess the application of pSEVA6BE-NG in *Pseudomonas* species, we selected two target spacers (HexR-2 and HexR-3 were inserted into pSEVA6BE-NG) with NG PAM in *hexR* using the online tool gBIG, and pSEVA6BE derivative plasmids were used as control. The results of HexR-2 and HexR-3 showed the conversion of C to T was present at the target sites with a CG or AG PAM (Figure 5B). Gene inactivation of both sites was achieved with an efficiency of 100%. Conversely, the pSEVA6BE-derived plasmids could not recognize non-NGG PAM target sites, thus all of these control strains failed to mutate C to T (Supplementary Figure S4). The application of pSEVA6BE-NG expands the candidate sites of the cytosine base editor in *P. putida* KT2440 and is equally applicable to other *Pseudomonas* species.

### Narrowing the Editable Window for Precision Editing

The CRISPR-assisted cytosine base editor exhibits a wide editable window width mainly from position 3 to position 8 (Chen et al., 2018; Rees and Liu, 2018), counting the first 5′ end nucleotide in the N20 sequence as position 1. The wide editing window allows substitution of multiple cytidines into thymines, but can also cause unpredictable editing effects when the desired target cytidine mutation is located among multiple cytidines. For example, the spacer HexR (5′-GCCCGGCAGATCCACTTCTT-3′) derived from *P. putida* KT2440 was selected as the target site for gene inactivation by the cytosine base editor. To achieve gene inactivation of *hexR*, the desired cytidine mutation should occur at position 7 by mutating CAG to TAG. However, DNA sequencing (Supplementary Figure S5) showed that six in ten edited colonies possessed C→T mutations at positions 3, 4, and 12, one colony had cytidines mutation at positions 4 and 18, and only three of the ten colonies displayed gene inactivation with cytidine substitutions at positions 3, 4, and 7. In another example presented in Figure 3F, the knockout efficiency of *ttgA* was only 25% when the editing window contained multiple cytidines. We hypothesize that a wide editing window can decrease the efficiency of gene inactivation by the cytosine base editor within a cytidine-rich target site due to the catalytic preference of APOBEC1 toward different NC motifs (in the order of TC ≥ CC ≥ AC > GC).

To obtain precise gene inactivation efficiency, we sought to narrow the editable window by introducing two mutations [W90Y and R126E (Kim et al., 2017)] into the APOBEC1 domain, generating pSEVA6BE-YE1 from pSEVA6BE (Figure 5C). Next, the spacer TtgA-2 was inserted into pSEVA6BE-YE1 to yield pSEVA6BE-YE1-TtgA-2. After base editing of pSEVA6BE-YE1-TtgA-2 in *P. putida* KT2440, we observed that the stop codon TAA was introduced successfully into five out of eight tested colonies (Figure 5D). By using pSEVA6BE-YE1, the knockout efficiency of the TtgA-2 spacer containing multiple cytidines increased from 25 to 62.5% in compared with using pSEVA6BE-TtgA. The construction of a cytosine base editor with a narrower editing window provides precision base editing of cytidine-rich sites.

### A Multiplex Base Editing System in *Pseudomonas putida* KT2440

A unique characteristic of the CRISPR/Cas9 system is the feasibility of concurrent multiplex genome editing, yet genome editing of two loci or more in eukaryotes has been the primary focus (Jakoèiunas et al., 2016) and few studies of multiplex genome editing have been reported for prokaryotes, which can be ascribed to the weak HDR and poor or lack of non-homologous end joining (NHEJ) repair (Wu et al., 2019b). Although multiplex genome editing of two loci by the CRISPR/Cas9 system had been tested in *P. putida* KT2440, the efficiency was extremely low and could not be applied readily in experiments (Aparicio et al., 2018).

Two multiplex base editing systems were constructed, that is, a one-plasmid system and two-plasmid system, to explore multiplex genome editing in *P. putida* KT2440. By connecting the base-editing cassette from pSEVA6BE with the broad-host-replicon RSF1010 and kanamycin-resistance marker from PVLT33, a kanamycin version of the base editing system pSEVA2BE (Supplementary Figure S6) was constructed and is compatible with pSEVA6BE in a two-plasmid system

**FIGURE 6:**
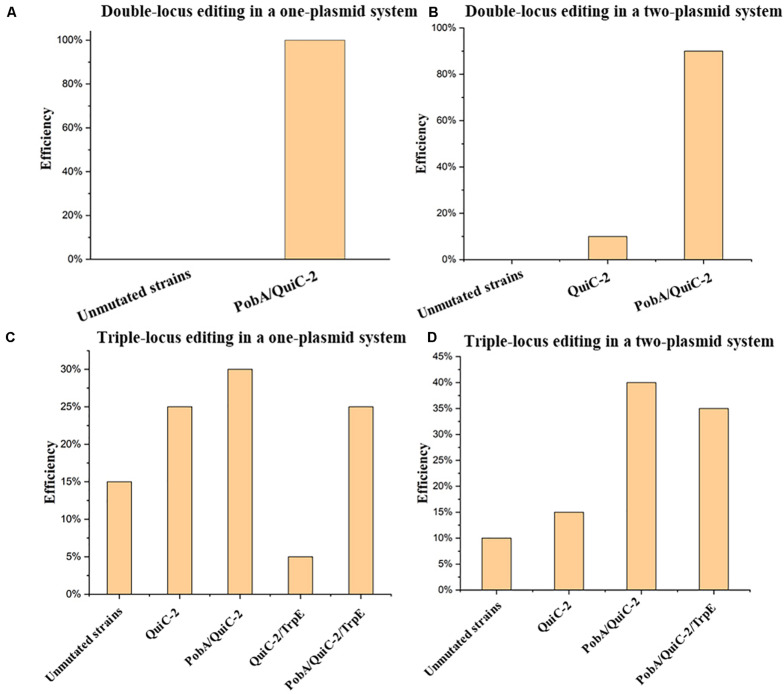
Assessment of multiplex base editing system in *Pseudomonas. putida* KT2440. **(A)** Double-locus editing of spacers PobA and QuiC-2 in the one-plasmid system pSEVA6BE-PobA-QuiC-2. **(B)** CRISPR-mediated double-locus base editing toward spacers PobA and QuiC-2 using a two-plasmid system. **(C)** Triple-locus base editing profiles (PobA, QuiC-2 and TrpE) using the one-plasmid system pSEVA6BE- PobA-QuiC-2-TrpE. **(D)** The two-plasmid system pSEVA6BE-PobA-TrpE/pSEVA2BE-QuiC-2-mediated triple-locus base editing in the *P. putida* KT2440.

In this study, multiplex base editing of a double-locus was tested initially for genomic loci *pobA* and *quiC*. Spacers PobA and QuiC-2 were inserted into pSEVA6BE and pSEVA2BE, respectively to generate the two-plasmid systems pSEVA6BE-PobA and pSEVA2BE-QuiC-2. An expression cassette containing the J23119 promoter, QuiC-2 spacer and sgRNA scaffold was inserted into pSEVA6BE-PobAto give the one-plasmid system pSEVA6BE-PobA-QuiC-2. After electroporation of the resulting plasmids, transformants were selected and cultivated according to base editing procedure. To confirm the substitution of C→T in the two loci (PobA and QuiC-2), ten randomly picked colonies in each system were used as DNA templates for colony PCR. The editing efficiency of the double-locus was 100 and 90% for the one-plasmid system and two-plasmid system (Figure 6A and Supplementary Figure S7), respectively.

Next, simultaneous base editing at three loci was tested using spacers TrpE, PobA and QuiC-2. *TrpE* encoding anthranilate synthase (TrpE) was selected as the third target site for multiple base editing. The TrpE editing cassette was inserted into pSEVA6BE-PobA-QuiC-2 and pSEVA6BE-PobA by using the same construction strategy to generate pSEVA6BE-PobA-QuiC-2-TrpE and pSEVA6BE-PobA-TrpE. After transformation of the one-plasmid (pSEVA6BE-PobA-QuiC-2-TrpE) or two-plasmid system (pSEVA6BE-PobA-TrpE and pSEVA2BE-QuiC-2) into *P. putida* KT2440 cells, the transformant colonies were identified, inoculated and cultivated in accordance with the previous cytosine base editing procedure. The simultaneous triple-locus gene inactivation efficiency was 25% (2/10 + 3/10) and 35% (4/10 + 3/10) for the one-plasmid and two-plasmid systems, respectively (Figure 6B and Supplementary Figure S8). The above results indicate that editing efficiency negatively correlates with the number of targeted spacers.

In our study, we investigated two kinds of system in multiplex base editing. The editing efficiency of double-locus and triple-locus showed no apparent difference between the one-plasmid and two-plasmid systems. The one-plasmid system gave a higher plasmid transformation efficiency when compared with that of the two-plasmid system. The two-plasmid system has the advantage of expressing different Cas9 proteins to recognize spacers with different PAM (Supplementary Figure S9). To test the feasibility of the two-plasmid system, the NG-recognize pSEVA6BE-NG-HexR3 and NGG-recognize pSEVA2BE-QuiC-2 were coelectroporated into *P. putida* KT2440. The sequencing results (Supplementary Figure S10) showed base editing can be achieved simultaneously in NG-spacer HexR3 and NGG-spacer QuiC-2 with an efficiency of 100%. The combination of different PAM-specificity Cas9 proteins in the two-plasmid system enriched the base editing scope of the one-step experiment.

Together, a fast and convenient multiplex base editing system has been established, which should facilitate construction of mutant libraries for metabolic engineering and synthetic biology in *P. putida* KT2440.

### Modification of *P. putida* KT2440 for Production of Protocatechuic Acid Using the Cytosine Base Editor

To construct a universal plasmid curing strategy for a base editing system in *Pseudomonas* spp., we inserted a counter-selection marker *sacB* into pSEVA6BE, pSEVA6BE-NG, pSEVA6BE-YE1 and pSEVA2BEto give pSEVA6BE-S, pSEVA6BE-NG-S, pSEVA6BE-YE1-S and pSEVA2BE-S (Supplementary Figure S11), respectively.

**FIGURE 7:**
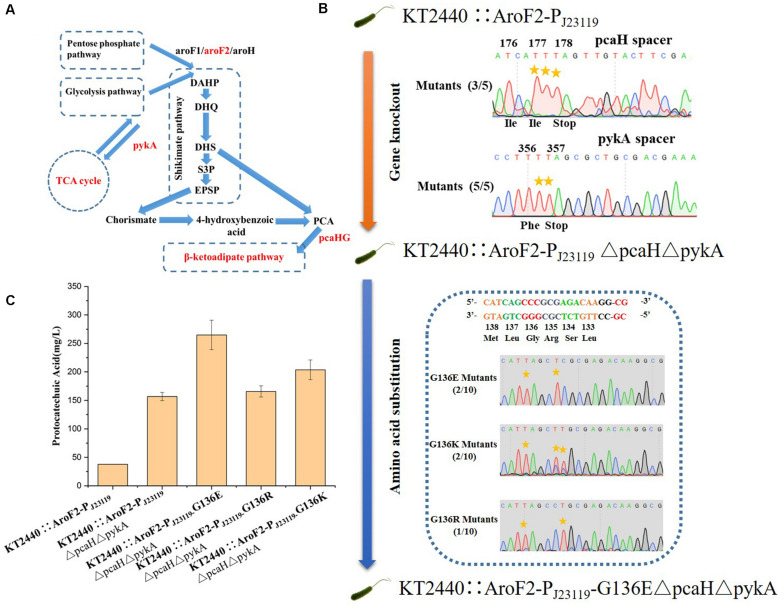
Protocatechuic acid (PCA) production titer in KT2440::AroF2-P_J__23119_-derived strains after modification by the base editor. **(A)** A schematic representation of PCA synthesis in *P. putida* KT2440. **(B)** Genomic modification of KT2440::AroF2-P_J__23119_ by gene knockout and amino acid substitution using the base editor. **(C)** The PCA titer in different KT2440::AroF2-P_J__23119_-derived strains after shaking-flask fermentation in King’s medium. Abbreviations: DAHP, 3-deoxy-D-arabinoheptulosonate-7-phosphate. DHQ, 3-dehydroquinate. DHS, 3-dehydroshikimate. S3P, shikimate-3-phosphate. EPSP, 5-enolpyruvyl-shikimate-5-phosphate. PCA, protocatechuic acid.

In addition to gene knockout, this base editing system can be used to make point mutations that relieve feedback inhibition in metabolic engineering. The application of our base editing system for metabolic engineering was tested by modifying *P. putida* KT2440 for the production of protocatechuic acid (PCA). The genes encoding protocatechuate 3,4-dioxygenase subunit beta (pcaH) and pyruvate kinase (pykA) (Figure 7A) were selected as knockout sites for enhancing the accumulation of PCA (Wang W. et al., 2018). The introduction of the G136E mutation was used to relieve feedback inhibition of the 3-deoxy-D-arabinoheptulosonate-7-phosphate (DAHP) synthase isozyme AroF-2 (Wynands et al., 2018). Cassettes targeting PcaH and PykA were inserted into pSEVA6BE-NG-S to create a double-locus editing plasmid pSEVA6BE-NG-pcaHpykA-S. The NG-PAM spacer AF1, which is adjacent G136 in AroF-2, was inserted into pSEVA6BE-NG-S to give pSEVA6BE-NG-AF1-S.

The *P. putida* KT2440 strain KT2440::AroF2-P_J__23119_ (containing a strong constitutive promoter P_J__23119_ inserted in the upstream region of AroF-2) was used as the starting strain for the production of PCA. We obtained the mutant strain KT2440::AroF2-P_J__23119_ΔpcaHΔpykA after double-locus base editing using pSEVA6BE-NG-pcaHpykA-S. After curing of the double-locus plasmid, pSEVA6BE-NG-AF1- S was transformed into KT2440::AroF2-P_J__23119_ΔpcaHΔpykA for achieving point mutations. The base editing results (Figure 7B) showed that mutations G136E, G136K, and G136R were introduced into genomic loci with efficiencies of 20, 20, and 10%, respectively. Next, the edited, unedited and starting strains were cultivated and used as seed cultures to transfer into King’s medium for shaking-flask experiments. After 66 h cultivation, the shaking flask fermentation showed that the production of PCA in the G136E mutation strain was 264.87 mg/L, which is an increase by 69.01 and 611.17% when compared with that of the AroF-2 unedited strain and the starting strain (Figure 7C). This study proved that the base editing system is a convenient tool for genomic modification in metabolic engineering.

## Discussion

*Pseudomonas putida* KT2440 is a potential chassis for industrial production of bio-based materials, pharmaceuticals and chemicals (Nikel et al., 2016). However, traditional genetic tools in *P. putida* KT2440 are difficult to manipulate and inefficient. The emergence of the CRISPR/Cas9 genome editing system has greatly simplified genetic engineering of *P. putida* KT2440, but still requires the provision of heterologous repair proteins and donor DNA templates (Aparicio et al., 2018; Sun et al., 2018). Additionally, simultaneous genome editing of two loci remains extremely challenging using the CRISPR/Cas9 system.

In the absence of templates and heterologous recombinases, the CRISPR-assisted cytosine deaminase system can achieve gene knockout or amino acid substitution, which is a quick and easy approach. Initially, the feasibility of cytosine base editing by expression of different cytidine deaminase modules was tested. After electroporation, strains containing pSEVA-Module 2 or pSEVA-Module 4 were cultivated at 30°C for 2–4 h after adding an inducer and then spread on LB agar containing antibiotics and inducer. However, all of the picked colonies from the plates were identified as wild-type strains. Based on this result and previous studies (Wang Y. et al., 2018; Li et al., 2019), we hypothesized that cytosine base editing could not fully perform its function in the initial transformant colonies, and that the cytosine base editor mutated only a fraction of the strains. By subculturing, we observed the C→T substitution of the target site, which demonstrated that a catalytic process for gradual accumulation was required for the generation of base editing mutants using the pSEVA6BE system in *P. putida* KT2440 cells.

In our study, we not only successfully developed the pSEVA6BE system as a knockout tool for *P. putida* KT2440, but also expanded the base-editing scope into *P. aeruginosa* PAO1, *P. fluorescens* Pf-5 and *P. entomophila* L48. *P. fluorescens* Pf-5 shows a great potential for application in biocontrol because of this strain possesses a wealth of antibacterial secondary metabolites (Paulsen et al., 2005). *P. entomophila* L48 is a model strain used for research of insect pathogenesis, which is hypothesized to produce hydrogen cyanide and novel secondary metabolites (Vodovar et al., 2006). Exploring the CRISPR-assisted cytosine base editing system scope in *P. fluorescens* Pf-5 and *P. entomophila* L48 will simplify genetic engineering of these strains and therefore advance research progress involving these strains.

During our study, Chen et al. (2018) reported a CRISPR-assisted cytosine base editor in multiple *Pseudomonas* strains, including *P. putida* KT2440 and *P. aeruginosa* PAO1. In this base editor, the expression of sgRNA and APOBEC1-nCas9 are under the control of constitutive promoters. The substitution of C to T can be identified from initial transformants after electroporation, which eliminates the required subculture step for colony generation. However, two limiting factors restrict its application in *Pseudomonas*: (i) High efficiency base editing was confined mainly to TC and CC motifs, with moderate efficiency toward the AC motif and no editing of the GC motif. (ii) The requirement of a NGG PAM reduces the candidate sites in target loci.

In our base editing system pSEVA6BE, cytosine base editing was achieved on all four NC motifs. The editing differences of Chen’s base editing system (Chen et al., 2018) and ours on the four motifs are possibly because of the different expression types of the APOBEC1-nCas9 complex. In our study, sgRNA and APOBEC1-nCas9-UGI were under the control of the PJ23119 constitutive promoter and arabinose-inducible promoter, respectively. The inducible expression of APOBEC1-nCas9-UGI generally yields higher expression levels when compared with that of the constitutive expression type used in the system reported by Chen et al. (2018), thereby achieving substitution of C to T in GC motif, even though this motif had the lowest catalytic order for APOBEC1. To expand the editing scope of the cytosine base editor, we constructed a NG-recognition base editor pSEVA6BE-NG by altering the PAM specificity of eSpCas9pp^D10A^. By utilizing the pSEVA6BE-NG system, we introduced a premature stop codon into the *HexR* gene with an efficiency of 100% (Figure 5A). Multiplex base editing of double and triple loci was validated by one-plasmid and two-plasmid systems, which proved to be a convenient method for double-deletion of the *pykA* and *pcaH* genes (Figure 7B). Taken together, our base editing system is a significant extension of the previous base editor (Chen et al., 2018) in *Pseudomonas*.

Although the base editor possesses C→T mutations on all four NC motifs, we observed that base editing could not be achieved or inefficient to realize in randomly selected spacers PA2018-2 (targeting the *PA2018* gene), HexR-5 (targeting the *PP_1021* gene) and HexR-4 (targeting the *PP_1021* gene), which contains a GC motif in the editable window (Supplementary Figures S12, S13). The catalytic preference of APOBEC1 toward different motifs (TC ≥ CC ≥ AC > GC) or off-target effects, or both of them were probably the major causes. To overcome these potential limitations, spacers PA2018, HexR, HexR-2, and HexR-3 were screened from these target genes by gBIG. By utilizing gBIG, these newly screened spacers PA2018, HexR, HexR-2, and HexR-3 do not contain a GC motif in target cytidines and the chance of off-target at these spacers was minimized. After the base editing procedure, DNA sequencing showed that spacers HexR (Supplementary Figure S5), PA2018 (Figure 4A), HexR-2 and HexR-3 (Figure 5B) exhibited significantly higher mutation efficiencies than the original spacers HexR-5, PA2018-2, and HexR-4. To minimize the motif preference of APOBEC1 or the disruption of off-target effects, the design of the 20-bp spacer with a NGG or NG PAM for gene inactivation should be assessed by base editing designing tools (Xiao et al., 2014; Hwang et al., 2018; Wang et al., 2019) in an effort to overcome this potential limitation (Wang et al., 2019).

Multiplex gene editing provides a fast and convenient tool for generating mutant strain libraries of *P. putida* KT2440. However, there are few studies reporting the use of CRISPR/Cas9-mediated multiplex genome editing in *Pseudomonas*, probably because of the lethality caused by DSBs or the relatively low efficiency of recombination afforded by recombination proteins. In our study, multiplex base editing in double and triple loci was achieved by using a one-plasmid or two-plasmid cytosine base editing system, which was simple and effective. By utilizing multiplex base editing system, double-deletion of the *pykA* and *pcaH* genes was achieved in one step experiment to generate the PCA-accumulating strain KT2440::AroF2-P_J__23119_ΔpcaHΔpykA. On the basis of the resulting strain, amino acid substitutions were successfully introduced to relieve feedback resistance of AroF-2, which increased the PCA titer to 264.87 mg/L.

In conclusion, this study demonstrated that a high-efficient CRISPR-assisted cytosine base editing system can be used to achieve multiplex gene editing in *P. putida* KT2440. Additionally, the accessibility and universality of the cytosine base editor was successfully extended to *P. aeruginosa* PAO1, *P. fluorescens* Pf-5 and *P. entomophila* L48. The established cytosine base editing system is an efficient tool to facilitate future research of *Pseudomonas* species.

## Data Availability Statement

The raw data supporting the conclusions of this article will be made available by the authors, without undue reservation, to any qualified researcher. Plasmids pSEVA6BE-S, pSEVA6BE-NG-S, pSEVA2BE-S, and pSEVA6BE-YE1-S are available on Addgene (Addgene IDs: 155266–155269).

## Author Contributions

JS, L-RY, and J-PW designed the experiments. JS, L-BL, and T-XL performed the experiments. JS analyzed the results and wrote the manuscript. All the authors contributed to the article and approved the submitted version.

## Conflict of Interest

The authors declare that the research was conducted in the absence of any commercial or financial relationships that could be construed as a potential conflict of interest.
