# Supplementary material for: CRISPR-Assisted Multiplex Base Editing System in Pseudomonas putida KT2440
Source: Front Bioeng Biotechnol. 2020 Jul 31;8:905. doi: 10.3389/fbioe.2020.00905 (PMC7413065; doi:10.3389/fbioe.2020.00905)
Supplement: Supplementary file 1 [file Table_1.docx]

**Table S1 Strains used in this study**

| **Original Strains** | **Relevant characteristics** | **Sources** |
| --- | --- | --- |
| *E. coli* DH5α | F- Φ80lacZΔM15, hsdR17 (rk-mk+)λ-, Δ(lacZYA-argF), U169, recA1, endA1, thi-1, gyrA96, relA, deoR, nupG,glnV44 | Lab stock |
| *Pseudomonas putida* KT2440 | mt-2 derivative, clearance of the TOL plasmid pWW0 | Lab stock |
| *Pseudomonas aeruginosa* PAO1 | Wild type | Lab stock |
| *Pseudomonas fluorescens* Pf-5 | Wild type | Lab stock |
| *Pseudomonas entomophila* L48 | Wild type | Lab stock |

**Table S2 Plasmids used in this study**

| **Plasmids** | **Relevant characteristics** | **Sources** |
| --- | --- | --- |
| pCAS-RK2T | oriRK2, Tet^R^, Pcas-Cas9, ParaB-Red, PrhaB-sgRNA-pRO1600, SacB | Lab stock |
| pSEVA-gRNAF | oriPRO1600/ColE1, Gm^R^, Pj23119-sgRNA-NicC | Lab stock |
| pSEVA-TtgA | oriPRO1600/ColE1, Gm^R^, Pj23119-sgRNA-TtgA | This study |
| pUC57-eSpCas9pp | Plasmid carrying an enhanced specificity Cas9 nuclease gene eSpCas9pp (Amp^R^) | Genscript |
| pUC57-APOBEC1 | Plasmid carrying rat cytidine deaminase gene APOBEC1 (Amp^R^) | Genscript |
| pCMV-BE3 | Plasmid carrying rAPOBEC1-XTEN linker-SpCas9(D10A)-UGI-NLS (Amp^R^) | Lab stock |
| pVLT33 | oriRSF1010, Km^R^, lacI-Ptac | Lab stock |
| pSEVA-Module 1 | oriPRO1600/ColE1, Gm^R^, Pj23119-sgRNA-TtgA, Pbs-APOBEC1-XTEN linker-SpCas9(D10A) | This study |
| pSEVA-Module 2 | oriPRO1600/ColE1, Gm^R^, Pj23119-sgRNA-TtgA, AraC-ParaBAD-APOBEC1-XTEN linker-SpCas9(D10A) | This study |
| pSEVA-Module 3 | oriPRO1600/ColE1, Gm^R^, Pj23119-sgRNA-TtgA, Pbs-APOBEC1-XTEN linker-eSpCas9pp(D10A) | This study |
| pSEVA-Module 4 | oriPRO1600/ColE1, Gm^R^, Pj23119-sgRNA-TtgA, AraC-ParaBAD-APOBEC1-XTEN linker -eSpCas9pp(D10A) | This study |
| pSEVA-Module 5 | oriPRO1600/ColE1, Gm^R^, Pj23119-sgRNA-TtgA, Xyls-Pm-APOBEC1-XTEN linker -eSpCas9pp(D10A) | This study |
| pSEVA-Module 6 /pSEVA6BE | oriPRO1600/ColE1, Gm^R^, Pj23119-sgRNA-TtgA, AraC-ParaBAD-APOBEC1-XTEN linker -eSpCas9pp(D10A)-UGI | This study |
| pSEVA2BE | oriRSF1010, Km^R^, Pj23119-sgRNA-TtgA, AraC-ParaBAD-APOBEC1-XTEN linker -eSpCas9pp(D10A)-UGI | This study |
| pSEVA6BE-NG | oriPRO1600/ColE1, Gm^R^, Pj23119-sgRNA-TtgA, AraC-ParaBAD-APOBEC1-XTEN linker -eSpCas9pp-NG(D10A, L1111R, D1135V, G1218R, E1219F, A1322R, R1335A and T1337R)-UGI | This study |
| pSEVA6BE-YE1 | oriPRO1600/ColE1, Gm^R^, Pj23119-sgRNA-TtgA, AraC-ParaBAD-APOBEC1-YE1(W90Y and R126E)-XTEN linker -eSpCas9pp(D10A)-UGI | This study |
| pSEVA6BE-HmgA | pSEVA6BE containing HmgA spacer, Gm^R^ | This study |
| pSEVA6BE-PobA | pSEVA6BE containing PobA spacer, Gm^R^ | This study |
| pSEVA6BE-QuiC | pSEVA6BE containing QuiC spacer, Gm^R^ | This study |
| pSEVA6BE-TtgA | pSEVA6BE containing TtgA spacer, Gm^R^ | This study |
| pSEVA6BE-TtgA-2 | pSEVA6BE containing TtgA-2 spacer, Gm^R^ | This study |
| pSEVA6BE-PA1236 | pSEVA6BE containing PA1236 spacer, Gm^R^ | This study |
| pSEVA6BE-PA2018 | pSEVA6BE containing PA2018 spacer, Gm^R^ | This study |
| pSEVA6BE-PFL0054 | pSEVA6BE containing PFL0054 spacer, Gm^R^ | This study |
| pSEVA6BE-PFL0556 | pSEVA6BE containing PFL0556 spacer, Gm^R^ | This study |
| pSEVA6BE-L48glpR | pSEVA6BE containing PSEEN1196 spacer, Gm^R^ | This study |
| pSEVA6BE-L48pykF | pSEVA6BE containing PSEEN1668 spacer, Gm^R^ | This study |
| pSEVA2BE-QuiC-2 | pSEVA2BE containing QuiC-2 spacer, Gm^R^ | This study |
| pSEVA6BE-PobA-TrpE | pSEVA6BE containing spacers PobA and TrpE, Gm^R^ | This study |
| pSEVA6BE-S | oriPRO1600/ColE1, Gm^R^, Pj23119-sgRNA-TtgA, AraC-ParaBAD-APOBEC1-XTEN linker -eSpCas9pp(D10A)-UGI, sacB | This study |
| pSEVA2BE-S | oriRSF1010, Km^R^, Pj23119-sgRNA-TtgA, AraC-ParaBAD-APOBEC1-XTEN linker -eSpCas9pp(D10A)-UGI, sacB | This study |
| pSEVA6BE-NG-S | pSEVA6BE-NG containing HexR-3 spacer, Gm^R^, sacB | This study |
| pSEVA6BE-YE1-S | pSEVA6BE-YE1 containing TtgA spacer, Gm^R^, sacB | This study |
| pSEVA6BE-PA2018-2 | pSEVA6BE containing PA2018-2 spacer, Gm^R^ | This study |
| pSEVA6BE-HexR-5 | pSEVA6BE containing HexR-5 spacer, Gm^R^ | This study |
| pSEVA6BE-HexR-4 | pSEVA6BE containing HexR-4 spacer, Gm^R^ | This study |
| pSEVA6BE-NG-HexR-2 | pSEVA6BE-NG containing HexR-2 spacer, Gm^R^ | This study |
| pSEVA6BE-NG-HexR-3 | pSEVA6BE-NG containing HexR-3 spacer, Gm^R^ | This study |
| pSEVA6BE-HexR-2 | pSEVA6BE containing HexR-2 spacer, Gm^R^ | This study |
| pSEVA6BE-HexR-3 | pSEVA6BE containing HexR-3 spacer, Gm^R^ | This study |
| pSEVA6BE-HexR | pSEVA6BE containing HexR spacer, Gm^R^ | This study |
| pSEVA6BE-YE-TtgA-2 | pSEVA6BE-YE containing TtgA-2 spacer, Gm^R^ | This study |
| pSEVA-Module 4-GllA | pSEVA-Module 4 containing GllA spacer, Gm^R^ | This study |
| pSEVA-Module 4-MexE | pSEVA-Module 4 containing MexE spacer, Gm^R^ | This study |
| pSEVA-Module 6-GllA | pSEVA-Module 6 containing GllA spacer, Gm^R^ | This study |
| pSEVA-Module 6-MexE | pSEVA-Module 6 containing MexE spacer, Gm^R^ | This study |
| pSEVA6BE-PobA-QuiC-2 | pSEVA6BE containing spacers PobA and QuiC-2, Gm^R^ | This study |
| pSEVA6BE-PobA-QuiC-2-TrpE | pSEVA6BE containing spacers PobA,QuiC-2 and TrpE, Gm^R^ | This study |
| pSEVA6BE-MexE | pSEVA6BE containing MexE spacer, Gm^R^ | This study |
| pSEVA6BE-MexE-QuiC-2 | pSEVA6BE containing spacers MexE and QuiC-2, Gm^R^ | This study |
| pSEVA6BE-NG-pcaHpykA-S | pSEVA6BE-NG-S containing spacers PcaH and PykA, Gm^R^, sacB | This study |
| pSEVA6BE-NG-AF1-S | pSEVA6BE-NG-S containing spacers AF1, Gm^R^, sacB | This study |

**Table S3 Primers used in this study**

| **Primers name** | **Primer sequence (5’ → 3’)** |
| --- | --- |
| S9-F | GATAAGAAATACTCAATAGGCTTAG |
| S9-R | TCAGTCACCTCCTAGCTGACTCAAATCAATG |
| U-F | TCTGGTGGTTCTACTAATCTGTCAGATATTATTG |
| U-R | AACAGGAGTCCAAGACTAGTTCAAGAACCACCAGAGAGCATC |
| eC9D10A-F | GGCCTGGCTATCGGCACCAACAGCG |
| eC9D10A-R | GTTGGTGCCGATAGCCAGGCCGATGCTG |
| C9D10A-F | CTTAGCTATCGGCACAAATAGCGTCGG |
| C9D10A-R | CTATTTGTGCCGATAGCTAAGCCTATTGAGTATTTC |
| 11-F | TGATCGCCCGCAAGAAGGACTGGGACCCGAAGAAGTACGGCGGCTTCGTCAGCCCGACCGTGGCC |
| 11-R | GTCCTTCTTGCGGGCGATCAGCTTGTCGCTGTTGCGCTTCGGCCGGATGCTTTCCTTGCTGAAGCC |
| 12-F | CAGCGCCCGCTTCCTGCAGAAGGGCAACGAAC |
| 12-R | TCTGCAGGAAGCGGGCGCTGGCCAGCATGCGC |
| 13-F | TTCGACACCACCATCGACCGCAAGGCCTACCGCAGCACCAAGGAAGTGC |
| 13-R | CGGTCGATGGTGGTGTCGAAGTACTTGAAGGCGCGCGGGGCGCCCAGG |
| YE-1F | CCGCTACCCGCACGTGACCCTGTTCATCTACATCGCCCGCCTGTACCACCACGCCGACCCGGAGAACCGCCAGGGCCTGCG |
| YE-1R | GGGTCACGTGCGGGTAGCGGCTCAGGAACTCGGTGATGGCGCGGCTGCATTCGCCGCACGGGCTATAGCTCAGGAACCAGG |
| eC9NG-1F | GACAAGAAGTACAGCATCGGCCTGGCTATCGGC |
| eC9NG-1R | CCGATGCTGTACTTCTTGTCACTTTCGGGTGTGGCGGACTC |
| eC9NG-2F | TCTGGTGGTTCTACTAATCTG |
| eC9NG-2R | AGATTAGTAGAACCACCAGAGTCGCCGCCCAGCTGGCTCAGG |
| BE-1F | TTAAGGGCGATAGGAGGAATATACCATGAGCAGCGAAACCGGCCCGG |
| BE-1R | TTCCTCCTATCGCCCTTAAGATGGAGAAACAGTAGAGAGTTG |
| BE-2F | GAGACTCCCGGGACCTCAGAGTCCGCCACACCCG |
| BE-2R | CTCTGAGGTCCCGGGAGTCTCGCTGCCGCTCTTCAGGCCGGTGGCCCACAGG |
| 62-1F | GGGTCCCCAATAATTACGATTTAAATTTGAC |
| 62-1R | ATCGTAATTATTGGGGACCCATCAAACAAAAGAGGAAAATAG |
| 62-2F | GCACCAGCGGCGCCTGAGAGGGGCGCGCCCAG |
| 62-2R | TCTCAGGCGCCGCTGGTGCTAAGCCATTGAATATAAAAGATAAAAATG |
| Sac-1F | CATTTTCTTTTGCGTTTTTATTTGTTAACTGTTAATTG |
| Sac-1R | AACAAATAAAAACGCAAAAGAAAATGCTGGATTCTCACCAATAAAAAACG |
| Sac-2F | GGGTCCCCAATAATTACGATTTAAATTTGACATAAGCC |
| Sac-2R | ATCGTAATTATTGGGGACCCATCAAACAAAAGAGGAAAATAG |
| C9-F | CCAAGAACCTGAGCGACGCCATCCTGC |
| C9-R | AGGCGTCGTGGGCGTGGTGGTAG |
| TtgA-gRNAF | TGCCAAACGATTGCAGGCTGGTTTTAGAGCTAGAAATAGCAAG |
| TtgA-gRNAR | CAGCCTGCAATCGTTTGGCAGCTAGCATTATACCTAGGAC |
| TtgA-2-gRNAF | CAAGCAACTGATTGACGAACGTTTTAGAGCTAGAAATAGCAAG |
| TtgA-2-gRNAR | GTTCGTCAATCAGTTGCTTGGCTAGCATTATACCTAGGAC |
| HmgA-gRNAF | GGGCAGAACTCCCCGCAGAGTTTTAGAGCTAGAAATAGCAAG |
| HmgA-gRNAR | TCTGCGGGGAGTTCTGCCCAGCTAGCATTATACCTAGGAC |
| PobA-gRNAF | AAAACTCAGGTTGCAATTATGTTTTAGAGCTAGAAATAGCAAG |
| PobA-gRNAR | ATAATTGCAACCTGAGTTTTGCTAGCATTATACCTAGGAC |
| QuiC-gRNAF | AACGTCCAGGCCGATGCCCTGTTTTAGAGCTAGAAATAGCAAG |
| QuiC-gRNAR | GGGCATCGGCCTGGACGTTGCTAGCATTATACCTAGGAC |
| PA1236-gRNAF | CTGCAGATAGTGCTGGAGGAGTTTTAGAGCTAGAAATAGCAAG |
| PA1236-gRNAR | TCCTCCAGCACTATCTGCAGGCTAGCATTATACCTAGGAC |
| PA2018-gRNAF | GTCAACCAAATGACCGCCACGTTTTAGAGCTAGAAATAGCAAG |
| PA2018-gRNAR | GTGGCGGTCATTTGGTTGACGCTAGCATTATACCTAGGAC |
| PFL0054-gRNAF | ATGCAGGACACCTACCCGGAGTTTTAGAGCTAGAAATAGCAAG |
| PFL0054-RNAR | TCCGGGTAGGTGTCCTGCATGCTAGCATTATACCTAGGAC |
| PFL0556-gRNAF | GTTGCAAGAGTTGCAGAACAGTTTTAGAGCTAGAAATAGCAAG |
| PFL0556-RNAR | TGTTCTGCAACTCTTGCAACGCTAGCATTATACCTAGGAC |
| L48glpR-gRNAF | CCAACAACAAATCCTCGAGCGTTTTAGAGCTAGAAATAGCAAG |
| L48glpR-gRNAR | GCTCGAGGATTTGTTGTTGGGCTAGCATTATACCTAGGAC |
| L48pykF-gRNAF | GGTGCAACTGCAACGCGGCCGTTTTAGAGCTAGAAATAGCAAG |
| L48pykF-gRNAR | GGCCGCGTTGCAGTTGCACCGCTAGCATTATACCTAGGAC |
| Quic2-gRNAF | TTACCAGCAAGTGTGGAACCGTTTTAGAGCTAGAAATAGCAAG |
| Quic2-gRNAR | GGTTCCACACTTGCTGGTAAGCTAGCATTATACCTAGGAC |
| TrpE-gRNAF | CAGGGCCAGGCACGTCTGCAGTTTTAGAGCTAGAAATAGCAAG |
| TrpE-gRNAR | TGCAGACGTGCCTGGCCCTGGCTAGCATTATACCTAGGAC |
| PA2018-2-RNAF | GTTGCAGGCGCTGCGCCGGGGTTTTAGAGCTAGAAATAGCAAG |
| PA2018-2-RNAR | CCCGGCGCAGCGCCTGCAACGCTAGCATTATACCTAGGAC |
| hexR-4-gRNAF | GCGCAGAGCCTGGCCAGTGGGTTTTAGAGCTAGAAATAGCAAG |
| hexR-4-gRNAR | CCACTGGCCAGGCTCTGCGCGCTAGCATTATACCTAGGAC |
| hexR-2-gRNAF | CAGATCCAGGGCCGCCTCGAGTTTTAGAGCTAGAAATAGCAAG |
| hexR-2-gRNAR | TCGAGGCGGCCCTGGATCTGGCTAGCATTATACCTAGGAC |
| hexR-3-gRNAF | GATCCAGGGCCGCCTCGACGGTTTTAGAGCTAGAAATAGCAAG |
| hexR-3-gRNAR | CGTCGAGGCGGCCCTGGATCGCTAGCATTATACCTAGGAC |
| hexR-5-gRNAF | GCAGTAGGTCAGCCGCGCCGGTTTTAGAGCTAGAAATAGCAAG |
| hexR-5-gRNAR | CGGCGCGGCTGACCTACTGCGCTAGCATTATACCTAGGAC |
| hexR-gRNAF | GCCCGGCAGATCCACTTCTTGTTTTAGAGCTAGAAATAGCAAG |
| hexR-gRNAR | AAGAAGTGGATCTGCCGGGCGCTAGCATTATACCTAGGAC |
| pykA-gF | CTTCCAGCGCTGCGACGAAGTTTTAGAGCTAGAAATAGCAAG |
| pykA-gR | TTCGTCGCAGCGCTGGAAGGGCTAGCATTATACCTAGGAC |
| PcaH-gF | TCACCCAGTTGTACTTCGAGTTTTAGAGCTAGAAATAGCAAG |
| PcaH-gR | TCGAAGTACAACTGGGTGATGCTAGCATTATACCTAGGAC |
| GllA-gF | ATCAGCCAGGTAATCACCTGTTTTAGAGCTAGAAATAGCAAG |
| GllA-gR | GGTGATTACCTGGCTGATCGCTAGCATTATACCTAGGAC |
| MexE-gF | GAACAACCGATCACCGAATGTTTTAGAGCTAGAAATAGCAAG |
| MexE-gR | TTCGGTGATCGGTTGTTCGGCTAGCATTATACCTAGGAC |
| AF1-gF | CATCAGCCCGCGAGACAAGGGTTTTAGAGCTAGAAATAGCAAG |
| AF1-gR | CCTTGTCTCGCGGGCTGATGGCTAGCATTATACCTAGGAC |

**Table S4. The sequences of base editing spacers used in this study.**

| **Spacer name** | **Sequence (5’ → 3’)** |
| --- | --- |
| TtgA (PP_1386) | TGCCAAACGATTGCAGGCTG |
| HmgA (PP_4621) | TGGGCAGAACTCCCCGCAGA |
| PobA (PP_3537) | AAAACTCAGGTTGCAATTAT |
| QuiC (PP_2554) | AACGTCCAGGCCGATGCCCT |
| TtgA-2 (PP_1386) | CAAGCAACTGATTGACGAAC |
| PA1236 | CTGCAGATAGTGCTGGAGGA |
| PA2018 | GTCAACCAAATGACCGCCAC |
| PFL0054 | ATGCAGGACACCTACCCGGA |
| PFL0556 | GTTGCAAGAGTTGCAGAACA |
| PSEEN1196 | CCAACAACAAATCCTCGAGC |
| PSEEN1668 | GGTGCAACTGCAACGCGGCC |
| QuiC-2 (PP_2554) | TTACCAGCAAGTGTGGAACC |
| TrpE (PP_0417) | CAGGGCCAGGCACGTCTGCA |
| PA2018-2 | GTTGCAGGCGCTGCGCCGGG |
| HexR-5 (PP_1021) | GCAGTAGGTCAGCCGCGCCG |
| HexR-4 (PP_1021) | GCGCAGAGCCTGGCCAGTGG |
| HexR-2 (PP_1021) | CAGATCCAGGGCCGCCTCGA |
| HexR-3 (PP_1021) | GATCCAGGGCCGCCTCGACG |
| HexR (PP_1021) | GCCCGGCAGATCCACTTCTT |
| PykA | CCTTCCAGCGCTGCGACGAA |
| PcaH | ATCACCCAGTTGTACTTCGA |
| GllA | GATCAGCCAGGTAATCACCT |
| MexE | CGAACAACCGATCACCGAAT |
| AF1 | CATCAGCCCGCGAGACAAGG |

**Figure S1 Base editing in GllA spacer by using pSEVA-Module 4 and pSEVA-Module 6**

**
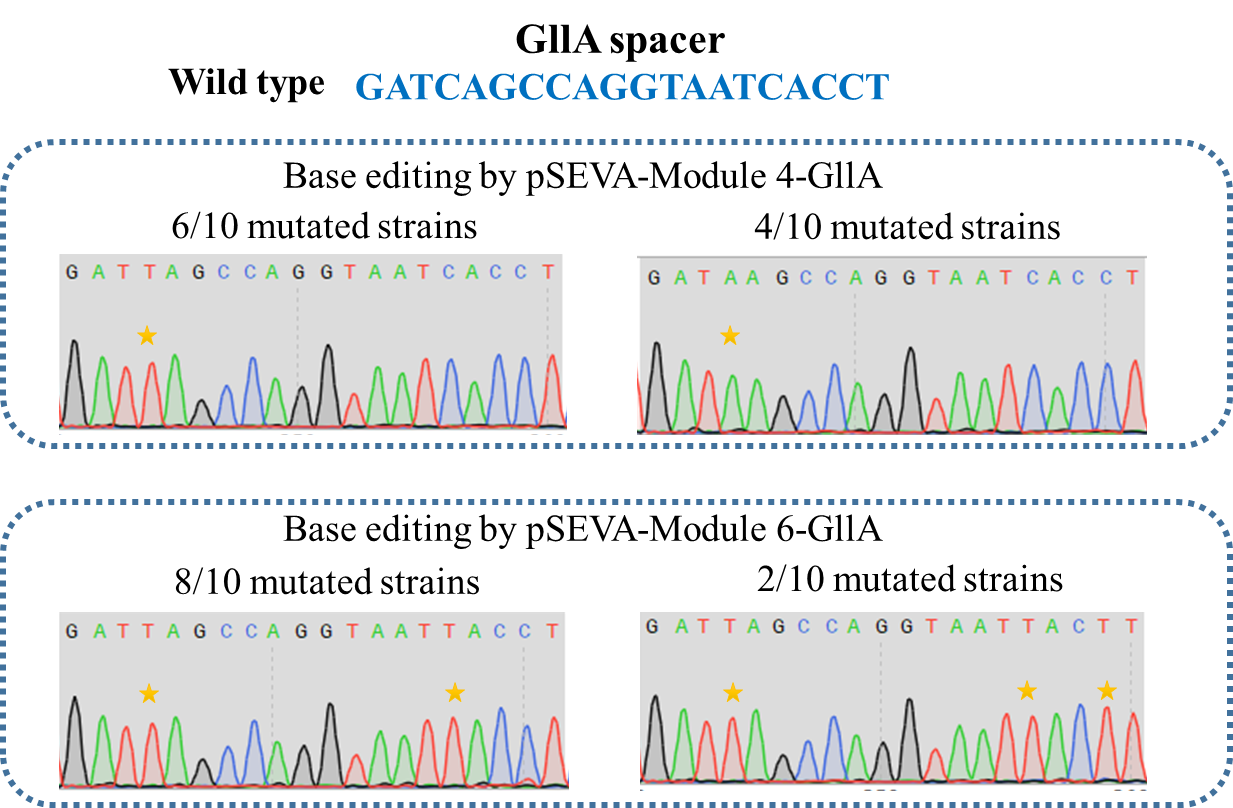
**

**Figure S2 Base editing in MexE spacer by using pSEVA-Module 4 and pSEVA-Module 6**

**
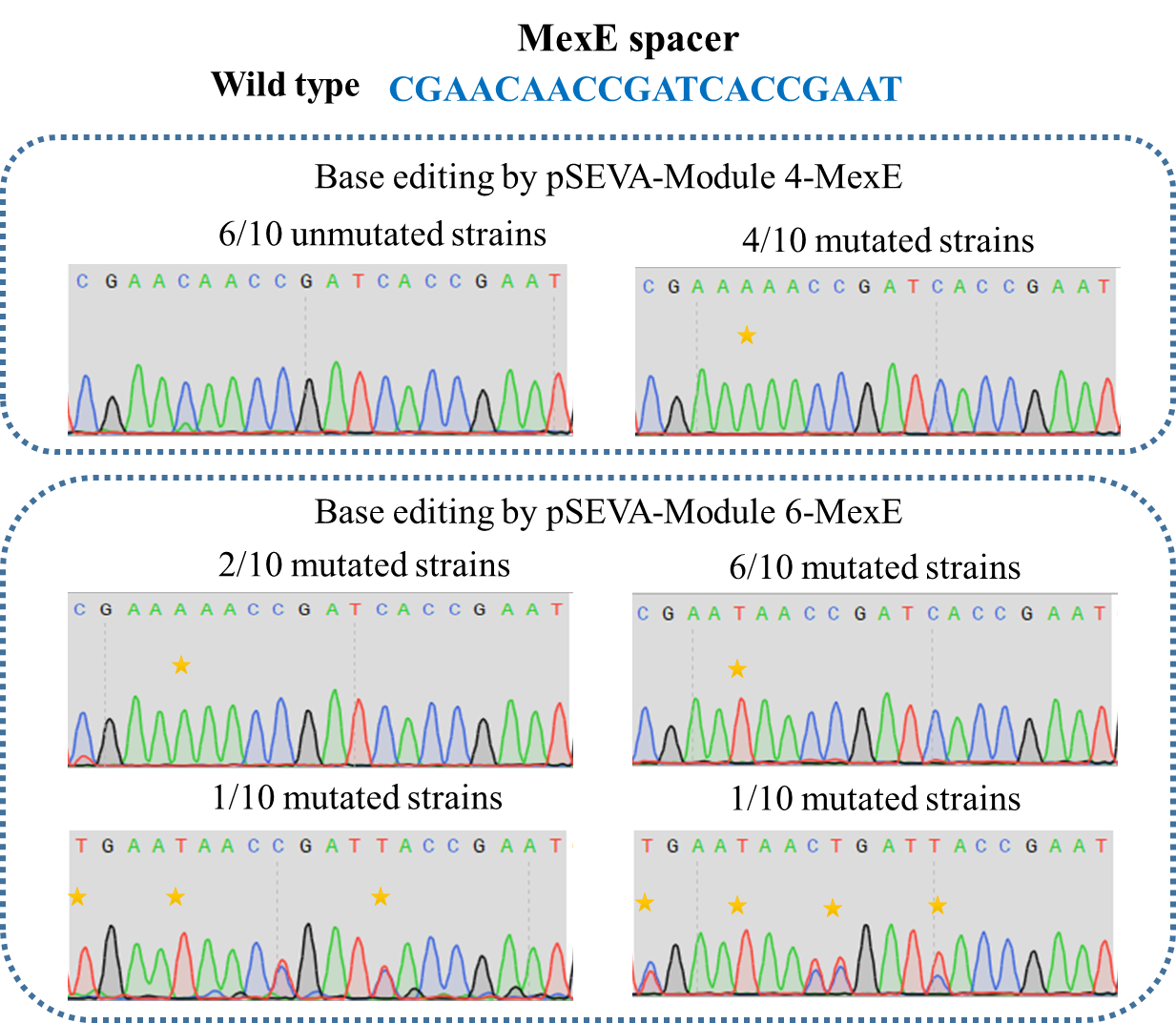
**

**Figure S3 Top three similar spacers of TtgA in the *P.putida* KT2440 genome and DNA sequencing results of these sites after base editing toward TtgA**

| **Spacer name** | **Sequence (5’-3’)** |
| --- | --- |
| **TtgA** | **TGCCAAACGATTGCAGGCTG** |
| **Spacer 1** | **TcCtgcAt_GATcGCAGGCTG-AGGG (888454-888477:-)** |
| **Spacer 2** | **caCgcgcC_GATgGCAGGCTG-TGGG (5584018-5584041:+)** |
| **Spacer 3** | **cGCtttgC_GATTtCAGGCTG-ACGG (1421323-1421346:-)** |

**DNA sequencing of the top three similar spacers of TtgA**

**
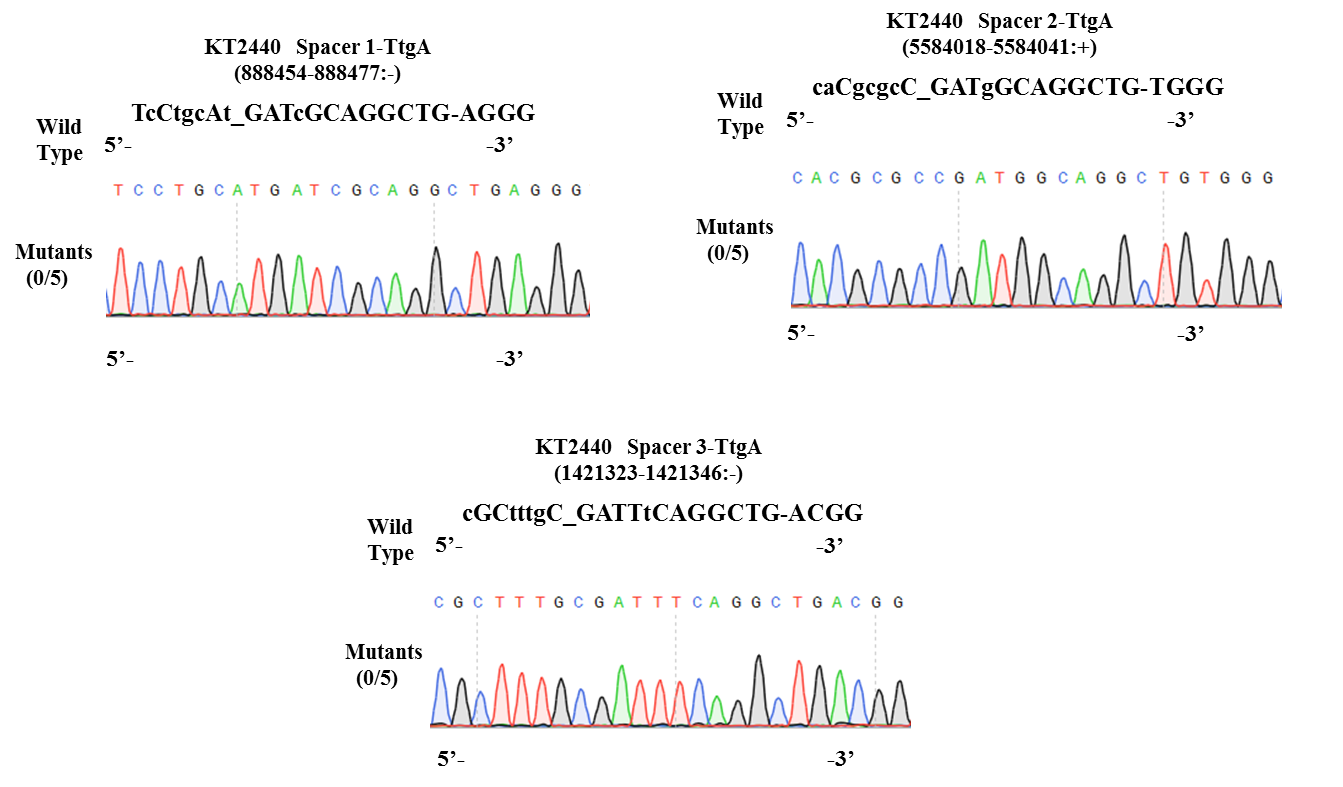
**

**Figure S4 DNA sequencing of spacers HexR-2 and HexR-3 after base editing using NGG- recognizing plasmids pSEVA6BE-HexR-2 and pSEVA6BE-HexR-3**

**
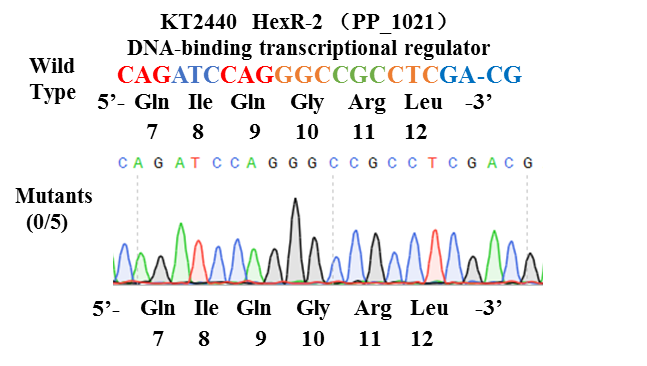
**

**DNA sequencing of HexR-2 spacer after base editing using pSEVA6BE-HexR-2**

**
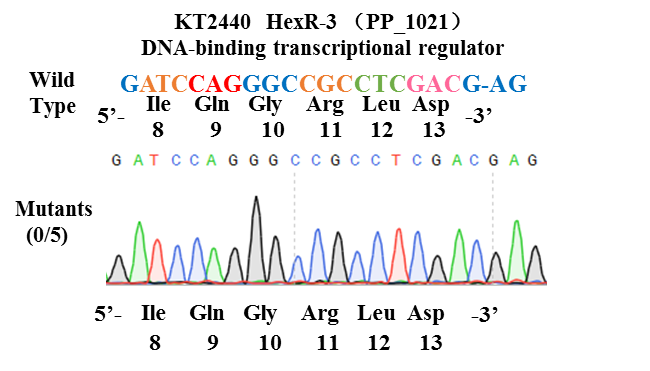
**

**DNA sequencing of HexR-3 spacer after base editing using pSEVA6BE-HexR-3**

**Figure S5** **Base editing in a cytidine-rich HexR spacer**


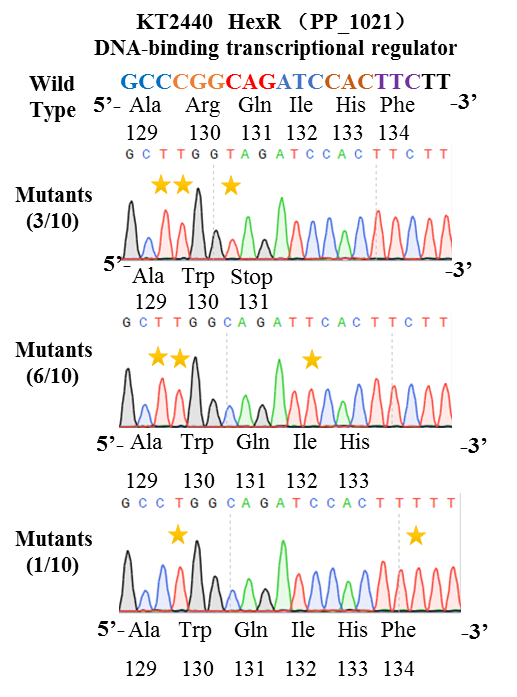


**Figure S6 The construction strategy of pSEVA2BE**

**
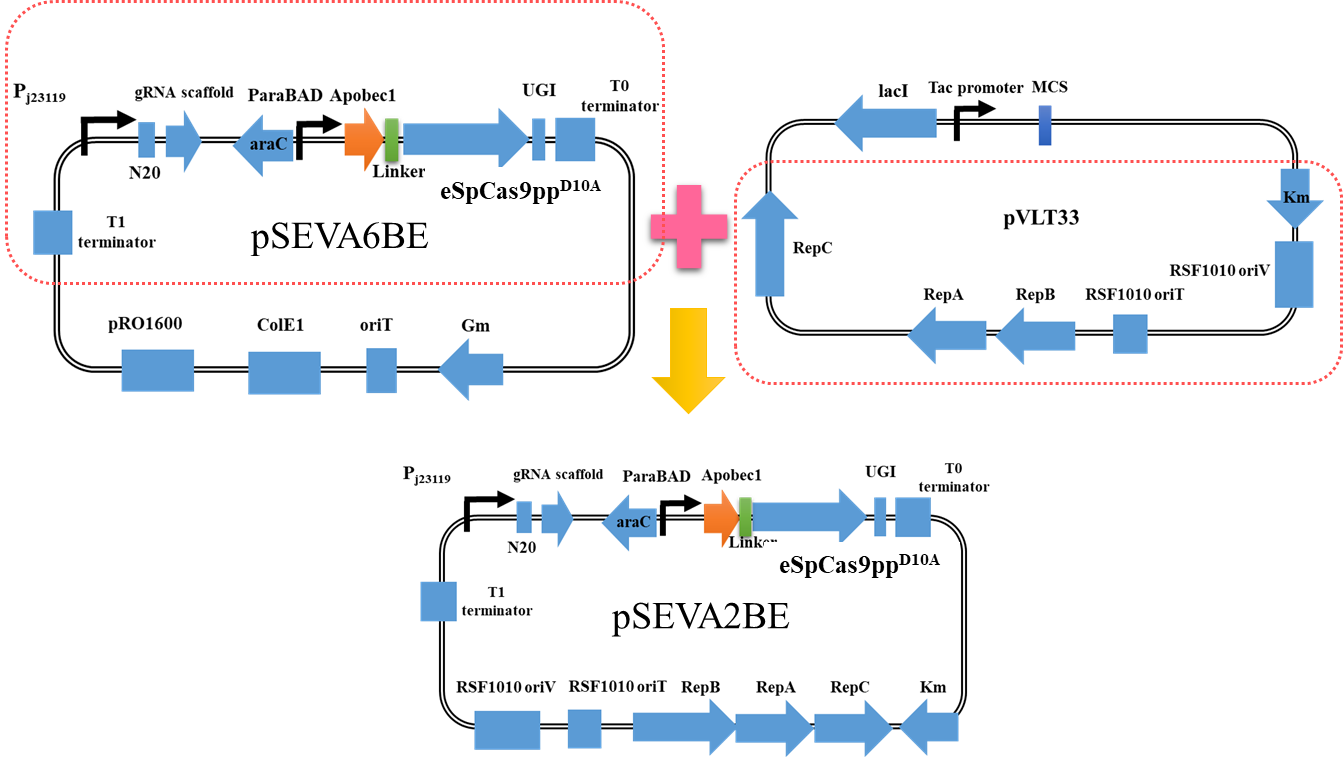
**

By connecting the base-editing cassette from pSEVA6BE with the broad-host-replicon RSF1010 and kanamycin-resistance marker from PVLT33, a kanamycin version of the base editing system pSEVA2BE (Figure S6) was constructed.

**Figure S7 Base editing in double-locus spacers PobA and QuiC-2**

**
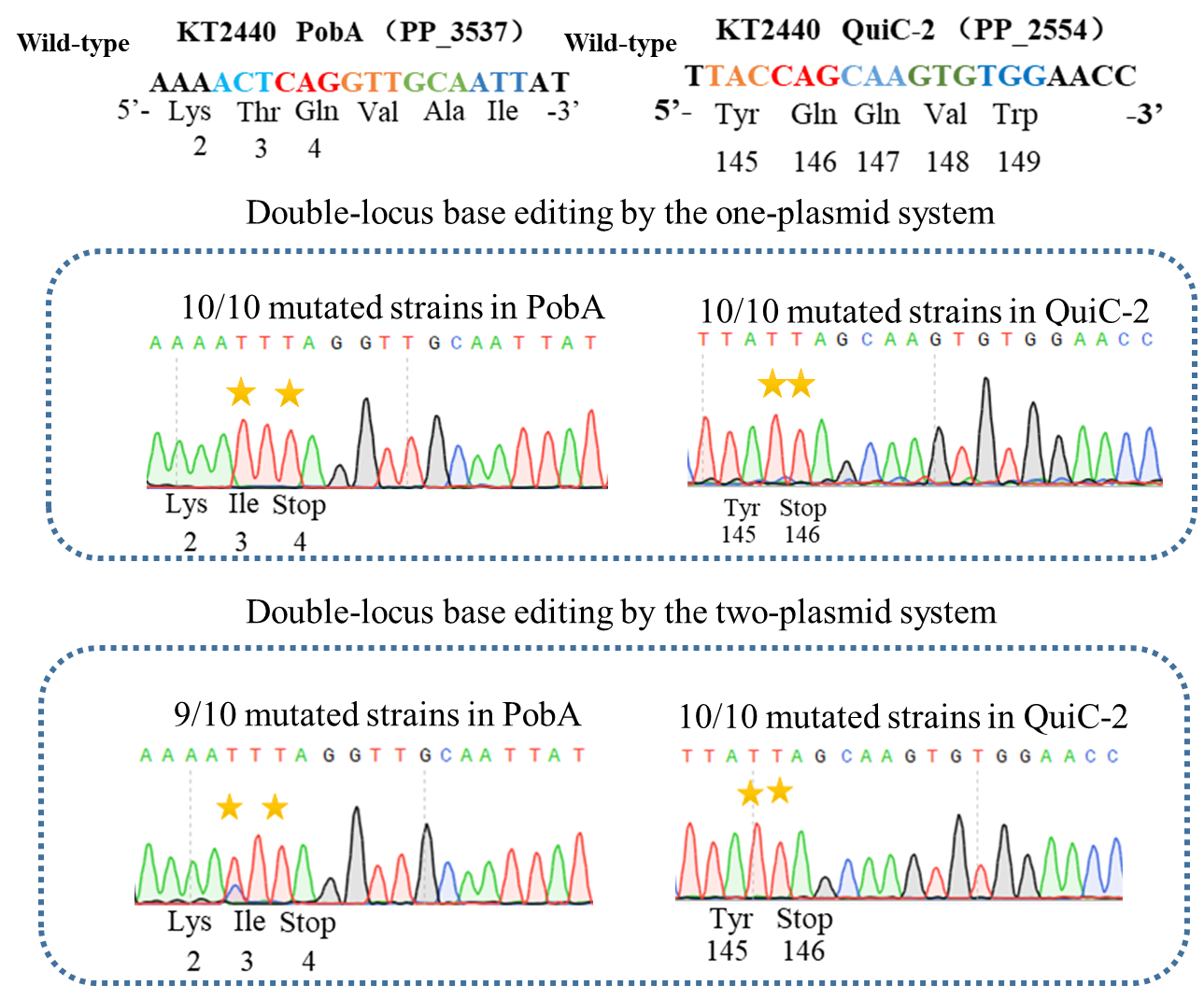
**

**Figure S8 Base editing in triple-locus spacers PobA, QuiC-2 and TrpE**

**
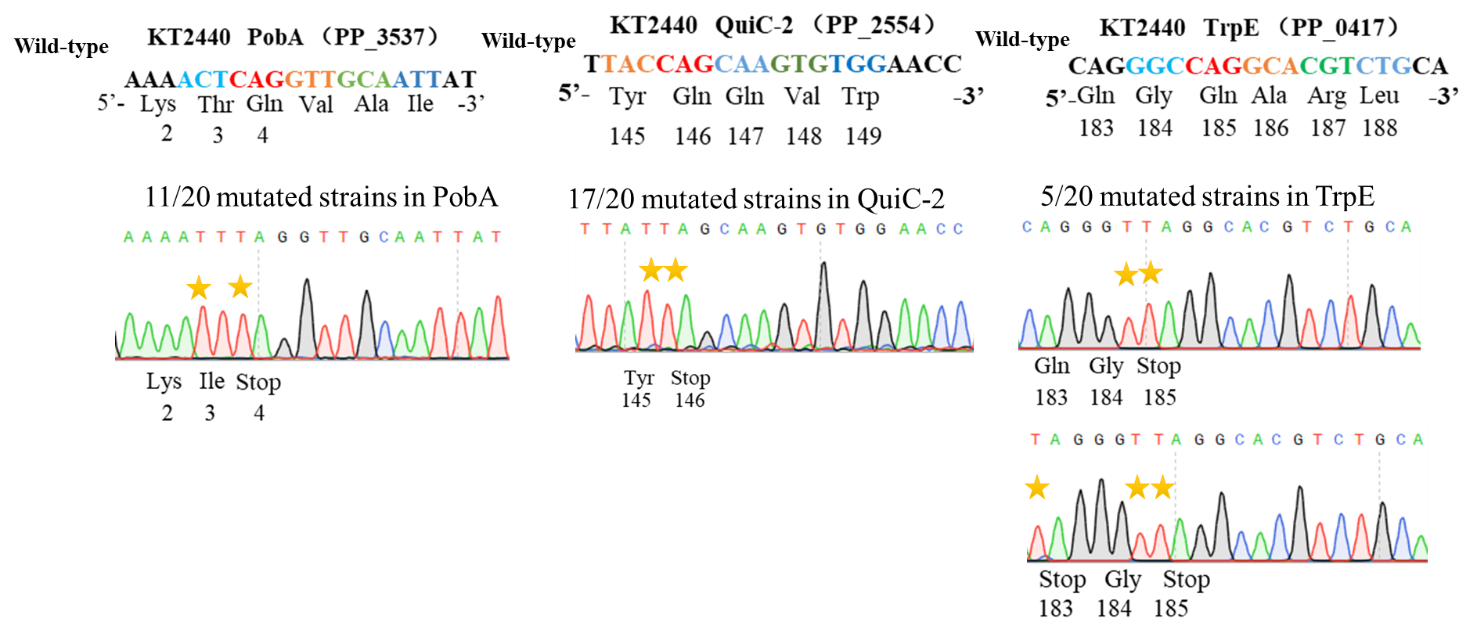
**

**DNA sequencing of triple-locus base editing in spacers PobA, QuiC-2 and TrpE using the one-plasmid system**

**
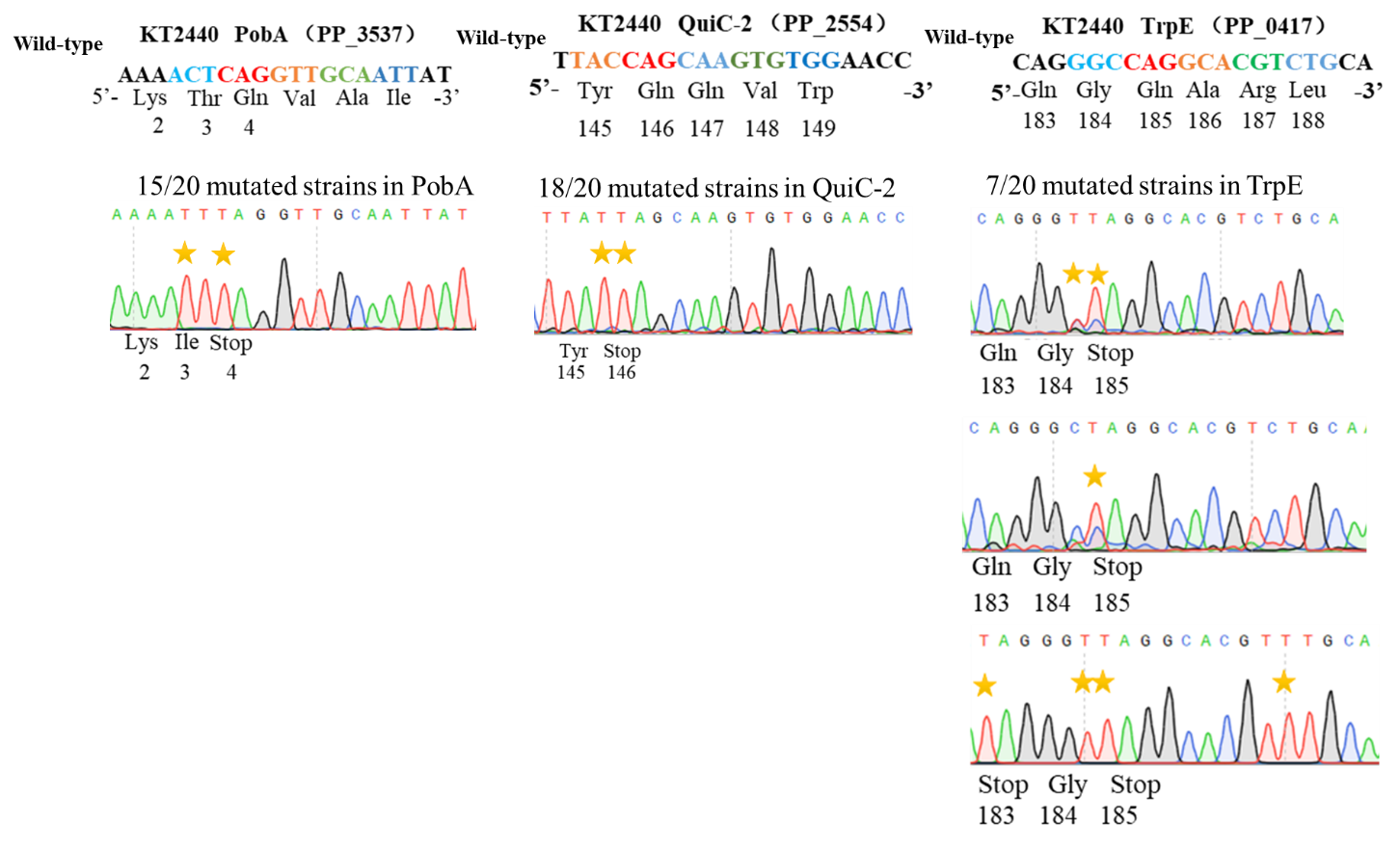
**

**DNA sequencing of triple-locus base editing in spacers PobA, QuiC-2 and TrpE using the two-plasmid system**

**Figure S9 Base editing of double-locus with different PAM sequences using a two-plasmid system**

**
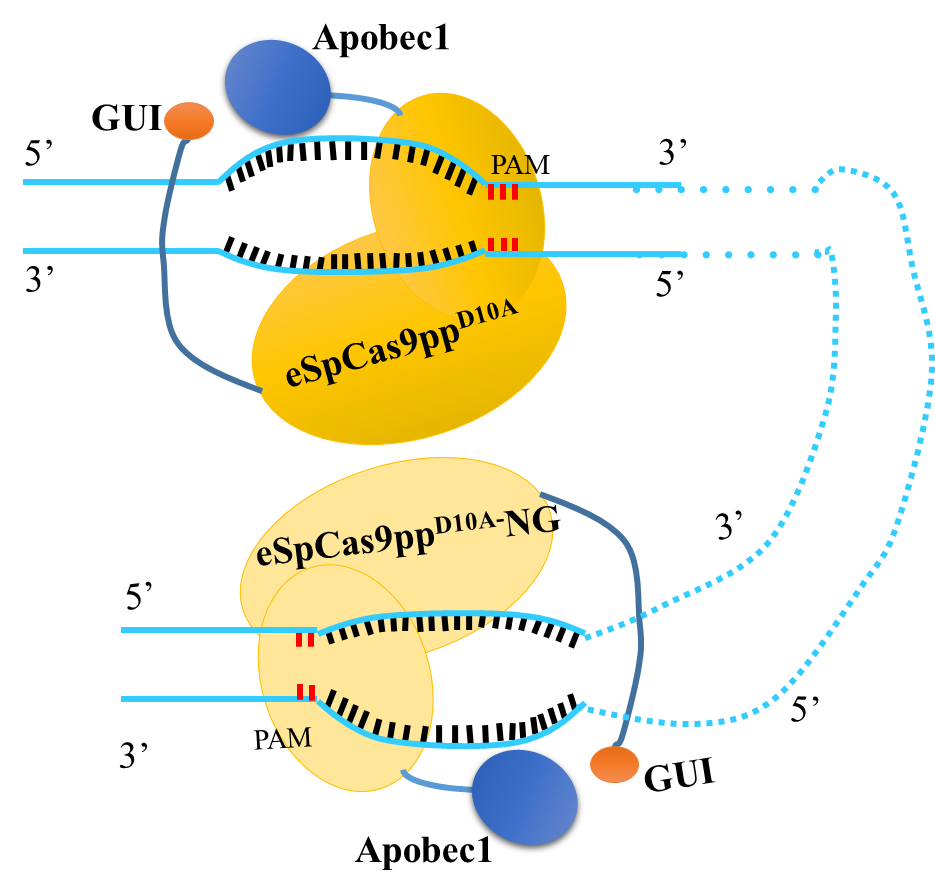
**

**Figure S10 DNA sequencing results of base editing in NG-type HexR3 spacer and NGG-type QuiC-2 spacer using a two-plasmid system**

**
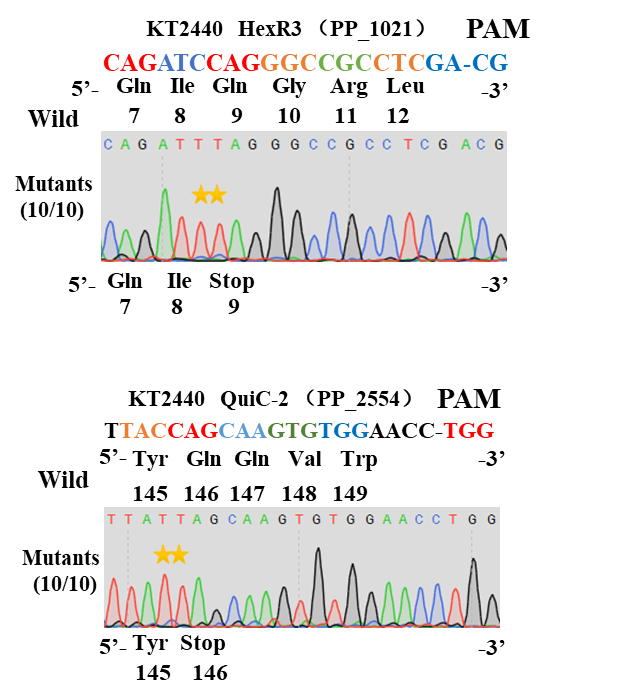
**

**Figure S11 Construction of a plasmid-curing strategy by appending with a counter-selection marker *sacB***

**
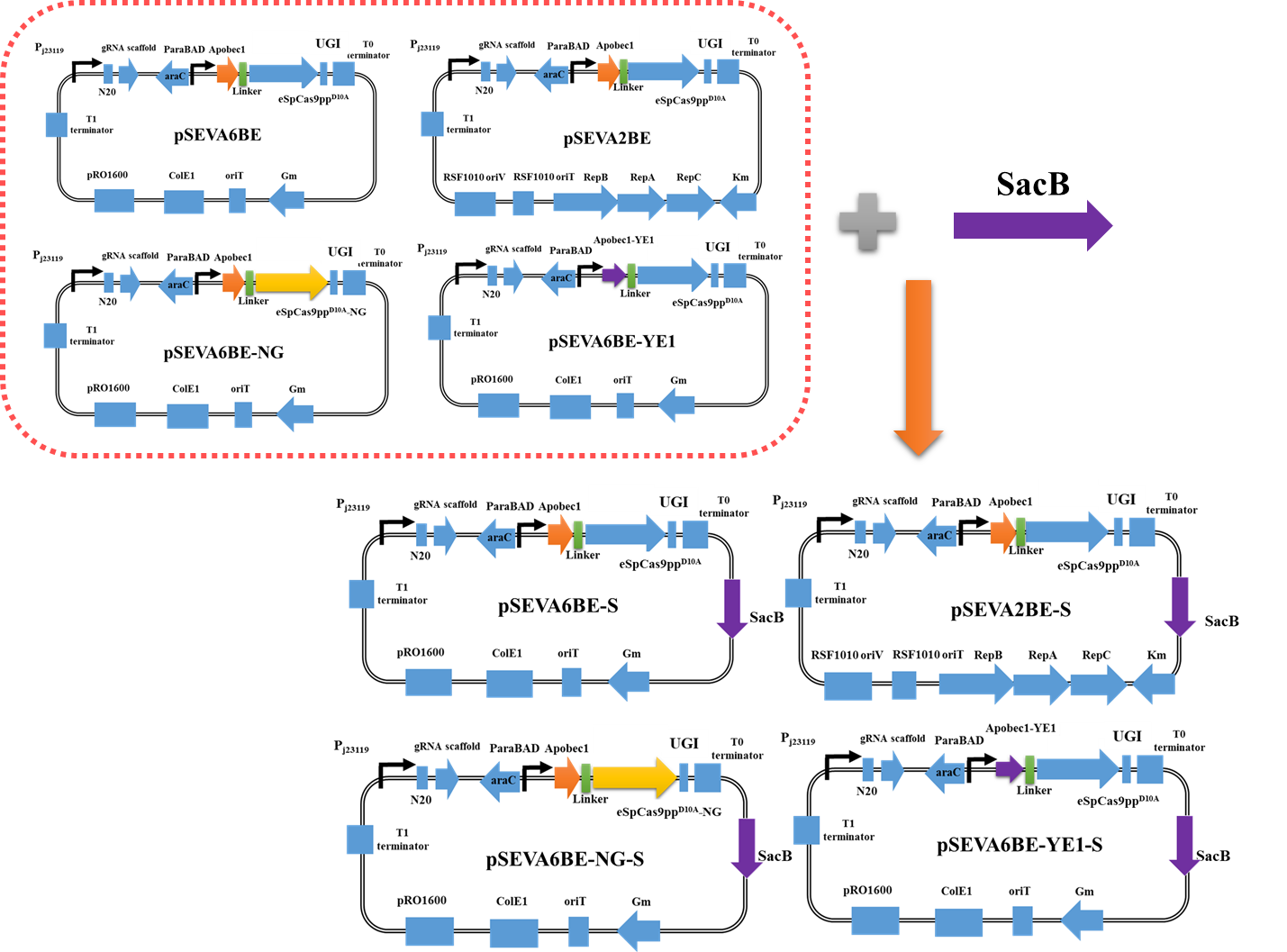
**

**Table S5 Potential off-target sites of spacers PA2018-2 and hexR-5**

| **Spacer name** | **Sequence** |
| --- | --- |
| **PA2018-2** | **GTTGCAGGCGCTGCGCCGGG** |
| **Spacer 1** | **cTgcgAGG_CGCTGCGCCGcG-AGGT (104290-104313:+)** |
| **Spacer 2** | **GgcGgAtG_CGCTGCGCCGGc-AGGC (351714-351737:+)** |
| **Spacer 3** | **cccGCAGG_CGCTGCGCCGGG-AGGG (426820-426843:+)** |
| **Spacer 4** | **cTcGCAGt_CGCTGCGCCaGG-AAGG (483385-483408:-)** |
| **Spacer 5** | **GTTGtgcG_CGtTGCGCCGGG-CGGT (511033-511056:+)** |
| **Spacer 6** | **GTTGCtGG_CGCTGCGCCaGG-CCGG (657777-657800:+)** |
| **Spacer 7** | **GcTcgAaG_CGCTGCGCCGGt-TGGC (686520-686543:+)** |
| **Spacer 8** | **GcgcaAGG_CGCTGCGCtGGG-CGGT (886710-886733:-)** |
| **Spacer 9** | **aTcGCtGG_CGCcGCGCCGGG-TCGG (1173438-1173461:-)** |
| **Spacer 10** | **caTcCAGG_CGCTGCtCCGGG-AAGT (1194962-1194985:+)** |
| **Spacer 11** | **cggtCtGc_CGCTGCGCCGGG-GAGG (1442849-1442872:+)** |
| **Spacer 12** | **GcaGCAGG_CGCTGCGCCaGG-CCGG (1490182-1490205:-)** |
| **Spacer 13** | **ccgaCAGG_CGCTGCGCCaGG-CGGC (1754980-1755003:+)** |
| **Spacer 14** | **cggcCAGG_CGCTGCGCCGGc-CGGC (1874807-1874830:-)** |
| **Spacer 15** | **tgTtCAGG_CGCTGCGCCaGG-GCGG (2060512-2060535:-)** |
| **Spacer 16** | **GaTaCccG_CGCTGCGCCtGG-AAGA (2259278-2259301:+)** |
| **Spacer 17** | **GcgGCgcG_CGCTGCGCCGGG-TGGC (2306075-2306098:+)** |
| **Spacer 18** | **GcTGatGG_CGCTGCGgCGGG-GAGA (2307769-2307792:+)** |
| **Spacer 19** | **ccTcCtGG_CGCgGCGCCGGG-GTGG (2377911-2377934:+)** |
| **Spacer 20** | **GcaaCAGG_CGCTGCGCCaGG-CGGA (2397940-2397963:+)** |
| **Spacer 21** | **GTcGtcGG_CGaTGCGCCGGG-CCGG (2565637-2565660:-)** |
| **Spacer 22** | **cTcGgAGc_CGCTGCGCCcGG-ACGG (2618888-2618911:+)** |
| **Spacer 23** | **cggGtAGt_CGCTGCGCCGGG-TCGG (2864827-2864850:+)** |
| **Spacer 24** | **GcTGgAac_CGCTGaGCCGGG-CGGC (3368924-3368947:+)** |
| **Spacer 25** | **tggcCAGG_CGCTGCGCCGcG-CGGG (3723616-3723639:-)** |
| **Spacer 26** | **cTacCtGG_CGCTGCGCCtGG-CGGC (3766330-3766353:-)** |
| **Spacer 27** | **GTTGCAGG_CGCaGCGCCGGG-AAGC (3809914-3809937:+)** |
| **Spacer 28** | **GTcGgtGa_CGCTGgGCCGGG-TGGC (3933993-3934016:-)** |
| **Spacer 29** | **GcgcCAGc_CGtTGCGCCGGG-TCGG (3954388-3954411:-)** |
| **Spacer 30** | **ccTGCtGG_CGCTGCGCCtGG-AGGA (4336248-4336271:-)** |
| **Spacer 31** | **GgaaCAGc_CGCTGCGCCtGG-CTGG (4516598-4516621:-)** |
| **Spacer 32** | **GTTcgAtt_gGCTGCGCCGGG-AGGC (4908305-4908328:+)** |
| **Spacer 33** | **GTTGagGc_CGCTGCGCaGGG-CGGC (5089778-5089801:+)** |
| **Spacer 34** | **caccCAGG_CGCTGCGCCtGG-CTGG (5288348-5288371:-)** |
| **Spacer 35** | **GcgGCcGG_CGCTGCGCCtGG-AAGT (5321367-5321390:+)** |
| **Spacer 36** | **cTccgAGG_CGCTGCGCCaGG-CGGC (5420357-5420380:-)** |
| **Spacer 37** | **ccgGCttG_CGCTGCGCCGGG-AAGA (5673910-5673933:+)** |
| **Spacer 38** | **cTTGaAct_CGCTGCGCCaGG-CGGT (5772477-5772500:+)** |

| **Spacer name** | **Sequence** |
| --- | --- |
| **HexR-5** | **GCAGCAGGTCAGCCGCGCCG** |
| **Spacer 1** | **cCtGaAGG_TCAGCCGCGgCG-CGGT (5158966-5158989:+)** |
| **Spacer 2** | **cgAaCAGa_TCcGCCGCGCCG-AGGC (2408182-2408205:-)** |
| **Spacer 3** | **aCAGCgtG_TCAGCCGCGaCG-AAGC (4252595-4252618:+)** |
| **Spacer 4** | **GgAGCgta_TCgGCCGCGCCG-TCGG (150576-150599:-)** |

**Figure S12** **DNA sequencing of spacers PA2018-2 and HexR-5**

**
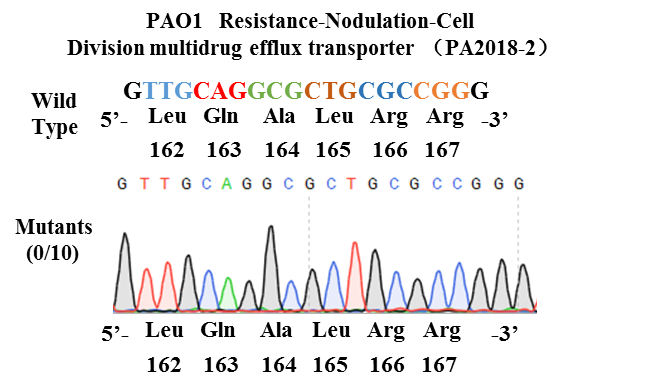
**

**
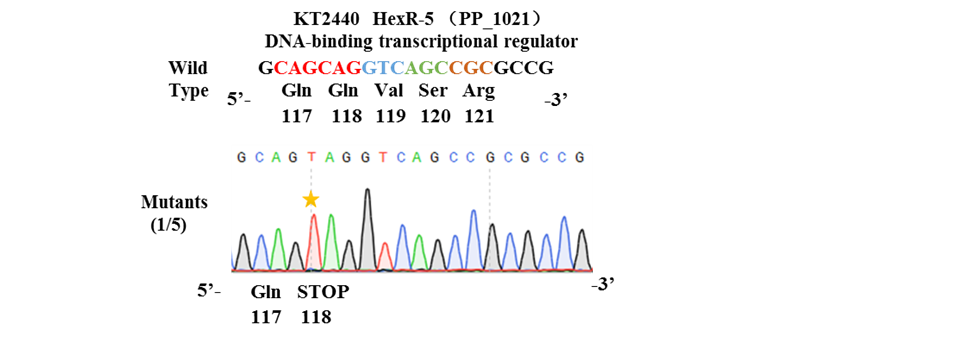
**

**Figure S13 Base editing in a NG PAM HexR-4 spacer**

**
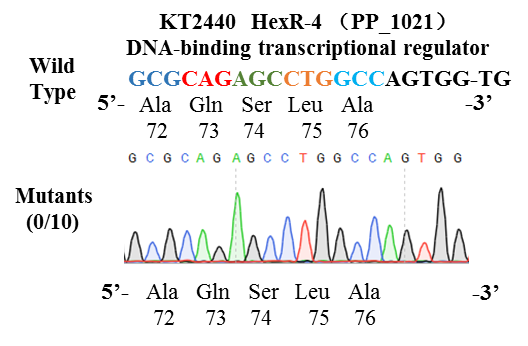
**

**DNA sequence of eSpCas9pp**

ATGGACAAGAAGTACAGCATCGGCCTGGACATCGGCACCAACAGCGTGGGCTGGGCCGTGATCACCGACGAGTACAAGGTGCCGAGCAAGAAGTTCAAGGTGCTGGGCAACACCGACCGCCACAGCATCAAGAAGAACCTGATCGGCGCCCTGCTGTTCGACAGCGGCGAGACCGCCGAAGCCACCCGCCTGAAGCGCACCGCCCGCCGCCGCTACACCCGTCGCAAGAACCGCATCTGCTACCTGCAGGAGATCTTCAGCAACGAAATGGCCAAGGTGGACGACAGCTTCTTCCACCGCCTGGAGGAAAGCTTCCTGGTGGAGGAAGACAAGAAGCACGAGCGCCACCCGATCTTCGGCAACATCGTGGACGAGGTGGCCTACCACGAAAAGTACCCGACCATCTACCACCTGCGCAAGAAGCTGGTCGACAGCACCGACAAGGCCGACCTGCGCCTGATCTACCTGGCCCTGGCCCACATGATCAAGTTCCGCGGCCACTTCCTGATCGAAGGCGACCTGAACCCGGACAACAGCGACGTGGACAAGCTGTTCATCCAGCTGGTGCAGACCTACAACCAGCTGTTCGAGGAAAACCCGATCAACGCCAGCGGCGTGGACGCCAAGGCCATCCTGAGCGCCCGCCTGAGCAAGAGCCGCCGCCTGGAGAACCTGATCGCCCAGCTGCCGGGCGAAAAGAAGAACGGCCTGTTCGGCAACCTGATCGCCCTGAGCCTGGGCCTGACCCCGAACTTCAAGAGCAACTTCGACCTGGCCGAGGACGCCAAGCTGCAGCTGAGCAAGGACACCTACGACGACGACCTGGACAACCTGCTGGCCCAGATCGGCGACCAGTACGCCGACCTGTTCCTGGCCGCCAAGAACCTGAGCGACGCCATCCTGCTGAGCGACATCCTGCGCGTGAACACCGAGATCACCAAGGCCCCGCTGAGCGCCAGCATGATCAAGCGCTACGACGAACACCACCAGGACCTGACCCTGCTGAAGGCCCTGGTGCGCCAGCAGCTGCCGGAGAAGTACAAGGAAATCTTCTTCGACCAGAGCAAGAACGGCTACGCCGGCTACATCGACGGTGGCGCCAGCCAGGAAGAGTTCTACAAGTTCATCAAGCCGATCCTGGAGAAGATGGACGGCACCGAGGAACTGCTGGTGAAGCTGAACCGCGAAGACCTGCTGCGCAAGCAGCGCACCTTCGACAACGGCAGCATCCCGCACCAGATCCACCTGGGCGAGCTGCACGCCATCCTGCGTCGCCAGGAAGACTTCTACCCGTTCCTGAAGGACAACCGCGAGAAGATCGAAAAGATCCTGACCTTCCGCATCCCGTACTACGTGGGCCCGCTGGCCCGCGGCAACAGCCGCTTCGCCTGGATGACCCGCAAGAGCGAGGAAACCATCACCCCGTGGAACTTCGAGGAAGTGGTGGACAAGGGCGCCAGCGCCCAGAGCTTCATCGAGCGCATGACCAACTTCGACAAGAACCTGCCGAACGAAAAGGTGCTGCCGAAGCACAGCCTGCTGTACGAGTACTTCACCGTGTACAACGAACTGACCAAGGTGAAGTACGTGACCGAGGGCATGCGCAAGCCGGCCTTCCTGAGCGGCGAACAGAAGAAGGCCATCGTGGACCTGCTGTTCAAGACCAACCGCAAGGTGACCGTGAAGCAGCTGAAGGAAGACTACTTCAAGAAGATCGAATGCTTCGACAGCGTGGAGATCAGCGGCGTGGAAGACCGCTTCAACGCCAGCCTGGGCACCTACCACGACCTGCTGAAGATCATCAAGGACAAGGACTTCCTGGACAACGAGGAAAACGAGGACATCCTGGAAGACATCGTGCTGACCCTGACCCTGTTCGAGGACCGCGAAATGATCGAGGAACGCCTGAAGACCTACGCCCACCTGTTCGACGACAAGGTGATGAAGCAGCTGAAGCGCCGCCGCTACACCGGCTGGGGCCGCCTGAGCCGCAAGCTGATCAACGGCATCCGCGACAAGCAGAGCGGCAAGACCATCCTGGACTTCCTGAAGAGCGACGGCTTCGCCAACCGCAACTTCATGCAGCTGATCCACGACGACAGCCTGACCTTCAAGGAAGACATCCAGAAGGCCCAAGTGAGCGGCCAGGGCGACAGCCTGCACGAACACATCGCCAACCTGGCCGGCAGCCCGGCCATCAAGAAGGGCATCCTGCAGACCGTGAAGGTGGTGGACGAGCTGGTGAAGGTGATGGGCCGCCACAAGCCGGAAAACATCGTGATCGAGATGGCCCGCGAAAACCAGACCACCCAGAAGGGCCAGAAGAACAGCCGCGAGCGCATGAAGCGCATCGAGGAAGGCATCAAGGAACTGGGCAGCCAGATCCTGAAGGAGCACCCGGTGGAAAACACCCAGCTGCAGAACGAGAAGCTGTACCTGTACTACCTGCAGAACGGCCGCGACATGTACGTGGACCAGGAACTGGACATCAACCGCCTGAGCGACTACGACGTGGACCACATCGTGCCGCAGAGCTTCCTGGCCGACGACAGCATCGACAACAAGGTGCTGACCCGCAGCGACAAGAACCGCGGCAAGAGCGACAACGTGCCGAGCGAGGAAGTGGTGAAGAAGATGAAGAACTACTGGCGCCAGCTGCTGAACGCCAAGCTGATCACCCAGCGCAAGTTCGACAACCTGACCAAGGCCGAGCGCGGCGGCCTGAGCGAACTGGACAAGGCCGGCTTCATCAAGCGCCAGCTGGTCGAGACCCGCCAGATCACCAAGCACGTGGCCCAGATCCTGGACAGCCGCATGAACACCAAGTACGACGAGAACGACAAGCTGATCCGCGAAGTGAAGGTGATCACCCTGAAGAGCAAGCTGGTCAGCGACTTCCGCAAGGACTTCCAGTTCTACAAGGTGCGCGAAATCAACAACTACCACCACGCCCACGACGCCTACCTGAACGCCGTGGTGGGCACCGCCCTGATCAAGAAGTACCCGGCCCTGGAGAGCGAATTCGTGTACGGCGACTACAAGGTGTACGACGTGCGCAAGATGATCGCCAAGAGCGAGCAGGAAATCGGCAAGGCCACCGCCAAGTACTTCTTCTACAGCAACATCATGAACTTCTTCAAGACCGAGATCACCCTGGCCAACGGCGAAATCCGCAAGGCCCCGCTGATCGAGACCAACGGCGAGACCGGCGAAATCGTGTGGGACAAGGGCCGCGACTTCGCCACCGTGCGCAAGGTGCTGAGCATGCCGCAGGTGAACATCGTGAAGAAGACCGAGGTGCAGACCGGCGGCTTCAGCAAGGAAAGCATCCTGCCGAAGCGCAACAGCGACAAGCTGATCGCCCGCAAGAAGGACTGGGACCCGAAGAAGTACGGCGGCTTCGACAGCCCGACCGTGGCCTACAGCGTGCTGGTCGTGGCCAAGGTGGAGAAGGGCAAGAGCAAGAAGCTGAAGAGCGTGAAGGAACTGCTGGGCATCACCATCATGGAGCGCAGCAGCTTCGAAAAGAACCCGATCGACTTCCTGGAGGCCAAGGGCTACAAGGAAGTGAAGAAGGACCTGATCATCAAGCTGCCGAAGTACAGCCTGTTCGAGCTGGAAAACGGCCGCAAGCGCATGCTGGCCAGCGCCGGCGAGCTGCAGAAGGGCAACGAACTGGCCCTGCCGAGCAAGTACGTGAACTTCCTGTACCTGGCCTCGCACTACGAGAAGCTGAAGGGCAGCCCGGAGGACAACGAACAGAAGCAGCTGTTCGTGGAGCAGCACAAGCACTACCTGGACGAGATCATCGAACAGATCAGCGAATTCAGCAAGCGCGTGATCCTGGCCGACGCCAACCTGGACAAGGTGCTGAGCGCCTACAACAAGCACCGCGACAAGCCGATCCGCGAGCAGGCCGAAAACATCATCCACCTGTTCACCCTGACCAACCTGGGCGCCCCGGCCGCCTTCAAGTACTTCGACACCACCATCGACCGCAAGCGCTACACCAGCACCAAGGAAGTGCTGGACGCCACCCTGATCCACCAGAGCATCACCGGCCTGTACGAAACCCGCATCGACCTGAGCCAGCTGGGCGGCGACTAG

**DNA sequence of APOBEC1**

ATGAGCAGCGAAACCGGCCCGGTGGCCGTGGACCCGACCCTGCGTCGCCGCATCGAGCCGCACGAGTTCGAAGTGTTCTTCGACCCGCGCGAGCTGCGCAAGGAAACCTGCCTGCTGTACGAAATCAACTGGGGCGGCCGCCACAGCATCTGGCGCCACACCAGCCAGAACACCAACAAGCACGTGGAGGTGAACTTCATCGAAAAGTTCACCACCGAGCGCTACTTCTGCCCGAACACCCGCTGCAGCATCACCTGGTTCCTGAGCTGGAGCCCGTGCGGCGAATGCAGCCGCGCCATCACCGAGTTCCTGAGCCGCTACCCGCACGTGACCCTGTTCATCTACATCGCCCGCCTGTACCACCACGCCGACCCGCGCAACCGCCAGGGCCTGCGCGACCTGATCAGCAGCGGCGTGACCATCCAGATCATGACCGAGCAGGAAAGCGGCTACTGCTGGCGCAACTTCGTGAACTACAGCCCGAGCAACGAAGCCCACTGGCCGCGCTACCCGCACCTGTGGGTGCGCCTGTACGTGCTGGAGCTGTACTGCATCATCCTGGGCCTGCCGCCGTGCCTGAACATCCTGCGTCGCAAGCAGCCGCAGCTGACCTTCTTCACCATCGCCCTGCAGAGCTGCCACTACCAGCGCCTGCCGCCGCACATCCTGTGGGCCACCGGCCTGAAGTAG

**DNA sequence of eSpCas9ppD10A-NG**

ATGGACAAGAAGTACAGCATCGGCCTGGATATCGGCACCAACAGCGTGGGCTGGGCCGTGATCACCGACGAGTACAAGGTGCCGAGCAAGAAGTTCAAGGTGCTGGGCAACACCGACCGCCACAGCATCAAGAAGAACCTGATCGGCGCCCTGCTGTTCGACAGCGGCGAGACCGCCGAAGCCACCCGCCTGAAGCGCACCGCCCGCCGCCGCTACACCCGTCGCAAGAACCGCATCTGCTACCTGCAGGAGATCTTCAGCAACGAAATGGCCAAGGTGGACGACAGCTTCTTCCACCGCCTGGAGGAAAGCTTCCTGGTGGAGGAAGACAAGAAGCACGAGCGCCACCCGATCTTCGGCAACATCGTGGACGAGGTGGCCTACCACGAAAAGTACCCGACCATCTACCACCTGCGCAAGAAGCTGGTCGACAGCACCGACAAGGCCGACCTGCGCCTGATCTACCTGGCCCTGGCCCACATGATCAAGTTCCGCGGCCACTTCCTGATCGAAGGCGACCTGAACCCGGACAACAGCGACGTGGACAAGCTGTTCATCCAGCTGGTGCAGACCTACAACCAGCTGTTCGAGGAAAACCCGATCAACGCCAGCGGCGTGGACGCCAAGGCCATCCTGAGCGCCCGCCTGAGCAAGAGCCGCCGCCTGGAGAACCTGATCGCCCAGCTGCCGGGCGAAAAGAAGAACGGCCTGTTCGGCAACCTGATCGCCCTGAGCCTGGGCCTGACCCCGAACTTCAAGAGCAACTTCGACCTGGCCGAGGACGCCAAGCTGCAGCTGAGCAAGGACACCTACGACGACGACCTGGACAACCTGCTGGCCCAGATCGGCGACCAGTACGCCGACCTGTTCCTGGCCGCCAAGAACCTGAGCGACGCCATCCTGCTGAGCGACATCCTGCGCGTGAACACCGAGATCACCAAGGCCCCGCTGAGCGCCAGCATGATCAAGCGCTACGACGAACACCACCAGGACCTGACCCTGCTGAAGGCCCTGGTGCGCCAGCAGCTGCCGGAGAAGTACAAGGAAATCTTCTTCGACCAGAGCAAGAACGGCTACGCCGGCTACATCGACGGTGGCGCCAGCCAGGAAGAGTTCTACAAGTTCATCAAGCCGATCCTGGAGAAGATGGACGGCACCGAGGAACTGCTGGTGAAGCTGAACCGCGAAGACCTGCTGCGCAAGCAGCGCACCTTCGACAACGGCAGCATCCCGCACCAGATCCACCTGGGCGAGCTGCACGCCATCCTGCGTCGCCAGGAAGACTTCTACCCGTTCCTGAAGGACAACCGCGAGAAGATCGAAAAGATCCTGACCTTCCGCATCCCGTACTACGTGGGCCCGCTGGCCCGCGGCAACAGCCGCTTCGCCTGGATGACCCGCAAGAGCGAGGAAACCATCACCCCGTGGAACTTCGAGGAAGTGGTGGACAAGGGCGCCAGCGCCCAGAGCTTCATCGAGCGCATGACCAACTTCGACAAGAACCTGCCGAACGAAAAGGTGCTGCCGAAGCACAGCCTGCTGTACGAGTACTTCACCGTGTACAACGAACTGACCAAGGTGAAGTACGTGACCGAGGGCATGCGCAAGCCGGCCTTCCTGAGCGGCGAACAGAAGAAGGCCATCGTGGACCTGCTGTTCAAGACCAACCGCAAGGTGACCGTGAAGCAGCTGAAGGAAGACTACTTCAAGAAGATCGAATGCTTCGACAGCGTGGAGATCAGCGGCGTGGAAGACCGCTTCAACGCCAGCCTGGGCACCTACCACGACCTGCTGAAGATCATCAAGGACAAGGACTTCCTGGACAACGAGGAAAACGAGGACATCCTGGAAGACATCGTGCTGACCCTGACCCTGTTCGAGGACCGCGAAATGATCGAGGAACGCCTGAAGACCTACGCCCACCTGTTCGACGACAAGGTGATGAAGCAGCTGAAGCGCCGCCGCTACACCGGCTGGGGCCGCCTGAGCCGCAAGCTGATCAACGGCATCCGCGACAAGCAGAGCGGCAAGACCATCCTGGACTTCCTGAAGAGCGACGGCTTCGCCAACCGCAACTTCATGCAGCTGATCCACGACGACAGCCTGACCTTCAAGGAAGACATCCAGAAGGCCCAAGTGAGCGGCCAGGGCGACAGCCTGCACGAACACATCGCCAACCTGGCCGGCAGCCCGGCCATCAAGAAGGGCATCCTGCAGACCGTGAAGGTGGTGGACGAGCTGGTGAAGGTGATGGGCCGCCACAAGCCGGAAAACATCGTGATCGAGATGGCCCGCGAAAACCAGACCACCCAGAAGGGCCAGAAGAACAGCCGCGAGCGCATGAAGCGCATCGAGGAAGGCATCAAGGAACTGGGCAGCCAGATCCTGAAGGAGCACCCGGTGGAAAACACCCAGCTGCAGAACGAGAAGCTGTACCTGTACTACCTGCAGAACGGCCGCGACATGTACGTGGACCAGGAACTGGACATCAACCGCCTGAGCGACTACGACGTGGACCACATCGTGCCGCAGAGCTTCCTGGCCGACGACAGCATCGACAACAAGGTGCTGACCCGCAGCGACAAGAACCGCGGCAAGAGCGACAACGTGCCGAGCGAGGAAGTGGTGAAGAAGATGAAGAACTACTGGCGCCAGCTGCTGAACGCCAAGCTGATCACCCAGCGCAAGTTCGACAACCTGACCAAGGCCGAGCGCGGCGGCCTGAGCGAACTGGACAAGGCCGGCTTCATCAAGCGCCAGCTGGTCGAGACCCGCCAGATCACCAAGCACGTGGCCCAGATCCTGGACAGCCGCATGAACACCAAGTACGACGAGAACGACAAGCTGATCCGCGAAGTGAAGGTGATCACCCTGAAGAGCAAGCTGGTCAGCGACTTCCGCAAGGACTTCCAGTTCTACAAGGTGCGCGAAATCAACAACTACCACCACGCCCACGACGCCTACCTGAACGCCGTGGTGGGCACCGCCCTGATCAAGAAGTACCCGGCCCTGGAGAGCGAATTCGTGTACGGCGACTACAAGGTGTACGACGTGCGCAAGATGATCGCCAAGAGCGAGCAGGAAATCGGCAAGGCCACCGCCAAGTACTTCTTCTACAGCAACATCATGAACTTCTTCAAGACCGAGATCACCCTGGCCAACGGCGAAATCCGCAAGGCCCCGCTGATCGAGACCAACGGCGAGACCGGCGAAATCGTGTGGGACAAGGGCCGCGACTTCGCCACCGTGCGCAAGGTGCTGAGCATGCCGCAGGTGAACATCGTGAAGAAGACCGAGGTGCAGACCGGCGGCTTCAGCAAGGAAAGCATCCGGCCGAAGCGCAACAGCGACAAGCTGATCGCCCGCAAGAAGGACTGGGACCCGAAGAAGTACGGCGGCTTCGTCAGCCCGACCGTGGCCTACAGCGTGCTGGTCGTGGCCAAGGTGGAGAAGGGCAAGAGCAAGAAGCTGAAGAGCGTGAAGGAACTGCTGGGCATCACCATCATGGAGCGCAGCAGCTTCGAAAAGAACCCGATCGACTTCCTGGAGGCCAAGGGCTACAAGGAAGTGAAGAAGGACCTGATCATCAAGCTGCCGAAGTACAGCCTGTTCGAGCTGGAAAACGGCCGCAAGCGCATGCTGGCCAGCGCCCGCTTCCTGCAGAAGGGCAACGAACTGGCCCTGCCGAGCAAGTACGTGAACTTCCTGTACCTGGCCTCGCACTACGAGAAGCTGAAGGGCAGCCCGGAGGACAACGAACAGAAGCAGCTGTTCGTGGAGCAGCACAAGCACTACCTGGACGAGATCATCGAACAGATCAGCGAATTCAGCAAGCGCGTGATCCTGGCCGACGCCAACCTGGACAAGGTGCTGAGCGCCTACAACAAGCACCGCGACAAGCCGATCCGCGAGCAGGCCGAAAACATCATCCACCTGTTCACCCTGACCAACCTGGGCGCCCCGCGCGCCTTCAAGTACTTCGACACCACCATCGACCGCAAGGCCTACCGCAGCACCAAGGAAGTGCTGGACGCCACCCTGATCCACCAGAGCATCACCGGCCTGTACGAAACCCGCATCGACCTGAGCCAGCTGGGCGGCGAC TAG

**DNA sequence of plasmid pSEVA6BE ( 9856 bp)**

**TTAATTAATGACACCATGAATTCTTGACAGCTAGCTCAGTCCTAGGTATAATGCTAGCTGCCAAACGATTGCAGGCTGGTTTTAGAGCTAGAAATAGCAAGTTAAAATAAGGCTAGTCCGTTATCAACTTGAAAAAGTGGCACCGAGTCGGTGCTTTTTTTGAGGAGCTCGGTCACCGTTACATATCAAAGGGAAAACTGTCCATACCCATGGGGCATGGCGAATTATGACAACTTGACGGCTACATCATTCACTTTTTCTTCACAACCGGCACGGAACTCGCTCGGGCTGGCCCCGGTGCATTTTTTAAATACCCGCGAGAAGTAGAGTTGATCGTCAAAACCAACATTGCGACCGACGGTGGCGATAGGCATCCGGGTGGTGCTCAAAAGCAGCTTCGCCTGGCTGATACGTTGGTCCTCGCGCCAGCTTAAGACGCTAATCCCTAACTGCTGGCGGAAAAGATGTGACAGACGCGACGGCGACAAGCAAACATGCTGTGCGACGCTGGCGATATCAAAATTGCTGTCTGCCAGGTGATCGCTGATGTACTGACAAGCCTCGCGTACCCGATTATCCATCGGTGGATGGAGCGACTCGTTAATCGCTTCCATGCGCCGCAGTAACAATTGCTCAAGCAGATTTATCGCCAGCAGCTCCGAATAGCGCCCTTCCCCTTGCCCGGCGTTAATGATTTGCCCAAACAGGTCGCTGAAATGCGGCTGGTGCGCTTCATCCGGGCGAAAGAACCCCGTATTGGCAAATATTGACGGCCAGTTAAGCCATTCATGCCAGTAGGCGCGCGGACGAAAGTAAACCCACTGGTGATACCATTCGCGAGCCTCCGGATGACGACCGTAGTGATGAATCTCTCCTGGCGGGAACAGCAAAATATCACCCGGTCGGCAAACAAATTCTCGTCCCTGATTTTTCACCACCCCCTGACCGCGAATGGTGAGATTGAGAATATAACCTTTCATTCCCAGCGGTCGGTCGATAAAAAAATCGAGATAACCGTTGGCCTCAATCGGCGTTAAACCCGCCACCAGATGGGCATTAAACGAGTATCCCGGCAGCAGGGGATCATTTTGCGCTTCAGCCATACTTTTCATACTCCCGCCATTCAGAGAAGAAACCAATTGTCCATATTGCATCAGACATTGCCGTCACTGCGTCTTTTACTGGCTCTTCTCGCTAACCAAACCGGTAACCCCGCTTATTAAAAGCATTCTGTAACAAAGCGGGACCAAAGCCATGACAAAAACGCGTAACAAAAGTGTCTATAATCACGGCAGAAAAGTCCACATTGATTATTTGCACGGCGTCACACTTTGCTATGCCATAGCATTTTTATCCATAAGATTAGCGGATCCTACCTGACGCTTTTTATCGCAACTCTCTACTGTTTCTCCATCTTAAGGGCGATAGGAGGAATATACCATGAGCAGCGAAACCGGCCCGGTGGCCGTGGACCCGACCCTGCGTCGCCGCATCGAGCCGCACGAGTTCGAAGTGTTCTTCGACCCGCGCGAGCTGCGCAAGGAAACCTGCCTGCTGTACGAAATCAACTGGGGCGGCCGCCACAGCATCTGGCGCCACACCAGCCAGAACACCAACAAGCACGTGGAGGTGAACTTCATCGAAAAGTTCACCACCGAGCGCTACTTCTGCCCGAACACCCGCTGCAGCATCACCTGGTTCCTGAGCTGGAGCCCGTGCGGCGAATGCAGCCGCGCCATCACCGAGTTCCTGAGCCGCTACCCGCACGTGACCCTGTTCATCTACATCGCCCGCCTGTACCACCACGCCGACCCGCGCAACCGCCAGGGCCTGCGCGACCTGATCAGCAGCGGCGTGACCATCCAGATCATGACCGAGCAGGAAAGCGGCTACTGCTGGCGCAACTTCGTGAACTACAGCCCGAGCAACGAAGCCCACTGGCCGCGCTACCCGCACCTGTGGGTGCGCCTGTACGTGCTGGAGCTGTACTGCATCATCCTGGGCCTGCCGCCGTGCCTGAACATCCTGCGTCGCAAGCAGCCGCAGCTGACCTTCTTCACCATCGCCCTGCAGAGCTGCCACTACCAGCGCCTGCCGCCGCACATCCTGTGGGCCACCGGCCTGAAGAGCGGCAGCGAGACTCCCGGGACCTCAGAGTCCGCCACACCCGAAAGTGACAAGAAGTACAGCATCGGCCTGGCTATCGGCACCAACAGCGTGGGCTGGGCCGTGATCACCGACGAGTACAAGGTGCCGAGCAAGAAGTTCAAGGTGCTGGGCAACACCGACCGCCACAGCATCAAGAAGAACCTGATCGGCGCCCTGCTGTTCGACAGCGGCGAGACCGCCGAAGCCACCCGCCTGAAGCGCACCGCCCGCCGCCGCTACACCCGTCGCAAGAACCGCATCTGCTACCTGCAGGAGATCTTCAGCAACGAAATGGCCAAGGTGGACGACAGCTTCTTCCACCGCCTGGAGGAAAGCTTCCTGGTGGAGGAAGACAAGAAGCACGAGCGCCACCCGATCTTCGGCAACATCGTGGACGAGGTGGCCTACCACGAAAAGTACCCGACCATCTACCACCTGCGCAAGAAGCTGGTCGACAGCACCGACAAGGCCGACCTGCGCCTGATCTACCTGGCCCTGGCCCACATGATCAAGTTCCGCGGCCACTTCCTGATCGAAGGCGACCTGAACCCGGACAACAGCGACGTGGACAAGCTGTTCATCCAGCTGGTGCAGACCTACAACCAGCTGTTCGAGGAAAACCCGATCAACGCCAGCGGCGTGGACGCCAAGGCCATCCTGAGCGCCCGCCTGAGCAAGAGCCGCCGCCTGGAGAACCTGATCGCCCAGCTGCCGGGCGAAAAGAAGAACGGCCTGTTCGGCAACCTGATCGCCCTGAGCCTGGGCCTGACCCCGAACTTCAAGAGCAACTTCGACCTGGCCGAGGACGCCAAGCTGCAGCTGAGCAAGGACACCTACGACGACGACCTGGACAACCTGCTGGCCCAGATCGGCGACCAGTACGCCGACCTGTTCCTGGCCGCCAAGAACCTGAGCGACGCCATCCTGCTGAGCGACATCCTGCGCGTGAACACCGAGATCACCAAGGCCCCGCTGAGCGCCAGCATGATCAAGCGCTACGACGAACACCACCAGGACCTGACCCTGCTGAAGGCCCTGGTGCGCCAGCAGCTGCCGGAGAAGTACAAGGAAATCTTCTTCGACCAGAGCAAGAACGGCTACGCCGGCTACATCGACGGTGGCGCCAGCCAGGAAGAGTTCTACAAGTTCATCAAGCCGATCCTGGAGAAGATGGACGGCACCGAGGAACTGCTGGTGAAGCTGAACCGCGAAGACCTGCTGCGCAAGCAGCGCACCTTCGACAACGGCAGCATCCCGCACCAGATCCACCTGGGCGAGCTGCACGCCATCCTGCGTCGCCAGGAAGACTTCTACCCGTTCCTGAAGGACAACCGCGAGAAGATCGAAAAGATCCTGACCTTCCGCATCCCGTACTACGTGGGCCCGCTGGCCCGCGGCAACAGCCGCTTCGCCTGGATGACCCGCAAGAGCGAGGAAACCATCACCCCGTGGAACTTCGAGGAAGTGGTGGACAAGGGCGCCAGCGCCCAGAGCTTCATCGAGCGCATGACCAACTTCGACAAGAACCTGCCGAACGAAAAGGTGCTGCCGAAGCACAGCCTGCTGTACGAGTACTTCACCGTGTACAACGAACTGACCAAGGTGAAGTACGTGACCGAGGGCATGCGCAAGCCGGCCTTCCTGAGCGGCGAACAGAAGAAGGCCATCGTGGACCTGCTGTTCAAGACCAACCGCAAGGTGACCGTGAAGCAGCTGAAGGAAGACTACTTCAAGAAGATCGAATGCTTCGACAGCGTGGAGATCAGCGGCGTGGAAGACCGCTTCAACGCCAGCCTGGGCACCTACCACGACCTGCTGAAGATCATCAAGGACAAGGACTTCCTGGACAACGAGGAAAACGAGGACATCCTGGAAGACATCGTGCTGACCCTGACCCTGTTCGAGGACCGCGAAATGATCGAGGAACGCCTGAAGACCTACGCCCACCTGTTCGACGACAAGGTGATGAAGCAGCTGAAGCGCCGCCGCTACACCGGCTGGGGCCGCCTGAGCCGCAAGCTGATCAACGGCATCCGCGACAAGCAGAGCGGCAAGACCATCCTGGACTTCCTGAAGAGCGACGGCTTCGCCAACCGCAACTTCATGCAGCTGATCCACGACGACAGCCTGACCTTCAAGGAAGACATCCAGAAGGCCCAAGTGAGCGGCCAGGGCGACAGCCTGCACGAACACATCGCCAACCTGGCCGGCAGCCCGGCCATCAAGAAGGGCATCCTGCAGACCGTGAAGGTGGTGGACGAGCTGGTGAAGGTGATGGGCCGCCACAAGCCGGAAAACATCGTGATCGAGATGGCCCGCGAAAACCAGACCACCCAGAAGGGCCAGAAGAACAGCCGCGAGCGCATGAAGCGCATCGAGGAAGGCATCAAGGAACTGGGCAGCCAGATCCTGAAGGAGCACCCGGTGGAAAACACCCAGCTGCAGAACGAGAAGCTGTACCTGTACTACCTGCAGAACGGCCGCGACATGTACGTGGACCAGGAACTGGACATCAACCGCCTGAGCGACTACGACGTGGACCACATCGTGCCGCAGAGCTTCCTGGCCGACGACAGCATCGACAACAAGGTGCTGACCCGCAGCGACAAGAACCGCGGCAAGAGCGACAACGTGCCGAGCGAGGAAGTGGTGAAGAAGATGAAGAACTACTGGCGCCAGCTGCTGAACGCCAAGCTGATCACCCAGCGCAAGTTCGACAACCTGACCAAGGCCGAGCGCGGCGGCCTGAGCGAACTGGACAAGGCCGGCTTCATCAAGCGCCAGCTGGTCGAGACCCGCCAGATCACCAAGCACGTGGCCCAGATCCTGGACAGCCGCATGAACACCAAGTACGACGAGAACGACAAGCTGATCCGCGAAGTGAAGGTGATCACCCTGAAGAGCAAGCTGGTCAGCGACTTCCGCAAGGACTTCCAGTTCTACAAGGTGCGCGAAATCAACAACTACCACCACGCCCACGACGCCTACCTGAACGCCGTGGTGGGCACCGCCCTGATCAAGAAGTACCCGGCCCTGGAGAGCGAATTCGTGTACGGCGACTACAAGGTGTACGACGTGCGCAAGATGATCGCCAAGAGCGAGCAGGAAATCGGCAAGGCCACCGCCAAGTACTTCTTCTACAGCAACATCATGAACTTCTTCAAGACCGAGATCACCCTGGCCAACGGCGAAATCCGCAAGGCCCCGCTGATCGAGACCAACGGCGAGACCGGCGAAATCGTGTGGGACAAGGGCCGCGACTTCGCCACCGTGCGCAAGGTGCTGAGCATGCCGCAGGTGAACATCGTGAAGAAGACCGAGGTGCAGACCGGCGGCTTCAGCAAGGAAAGCATCCTGCCGAAGCGCAACAGCGACAAGCTGATCGCCCGCAAGAAGGACTGGGACCCGAAGAAGTACGGCGGCTTCGACAGCCCGACCGTGGCCTACAGCGTGCTGGTCGTGGCCAAGGTGGAGAAGGGCAAGAGCAAGAAGCTGAAGAGCGTGAAGGAACTGCTGGGCATCACCATCATGGAGCGCAGCAGCTTCGAAAAGAACCCGATCGACTTCCTGGAGGCCAAGGGCTACAAGGAAGTGAAGAAGGACCTGATCATCAAGCTGCCGAAGTACAGCCTGTTCGAGCTGGAAAACGGCCGCAAGCGCATGCTGGCCAGCGCCGGCGAGCTGCAGAAGGGCAACGAACTGGCCCTGCCGAGCAAGTACGTGAACTTCCTGTACCTGGCCTCGCACTACGAGAAGCTGAAGGGCAGCCCGGAGGACAACGAACAGAAGCAGCTGTTCGTGGAGCAGCACAAGCACTACCTGGACGAGATCATCGAACAGATCAGCGAATTCAGCAAGCGCGTGATCCTGGCCGACGCCAACCTGGACAAGGTGCTGAGCGCCTACAACAAGCACCGCGACAAGCCGATCCGCGAGCAGGCCGAAAACATCATCCACCTGTTCACCCTGACCAACCTGGGCGCCCCGGCCGCCTTCAAGTACTTCGACACCACCATCGACCGCAAGCGCTACACCAGCACCAAGGAAGTGCTGGACGCCACCCTGATCCACCAGAGCATCACCGGCCTGTACGAAACCCGCATCGACCTGAGCCAGCTGGGCGGCGACTCTGGTGGTTCTACTAATCTGTCAGATATTATTGAAAAGGAAACCGGTAAGCAACTGGTTATCCAGGAATCCATCCTCATGCTCCCAGAGGAGGTGGAAGAAGTCATTGGGAACAAGCCGGAAAGCGATATACTCGTGCACACCGCCTACGACGAGAGCACCGACGAGAATGTCATGCTTCTGACTAGCGACGCCCCTGAATACAAGCCTTGGGCTCTGGTCATACAGGATAGCAACGGTGAGAACAAGATTAAGATGCTCTCTGGTGGTTCTTGAACTAGTCTTGGACTCCTGTTGATAGATCCAGTAATGACCTCAGAACTCCATCTGGATTTGTTCAGAACGCTCGGTTGCCGCCGGGCGTTTTTTATTGGTGAGAATCCAGGGGTCCCCAATAATTACGATTTAAATTTGACATAAGCCTGTTCGGTTCGTAAACTGTAATGCAAGTAGCGTATGCGCTCACGCAACTGGTCCAGAACCTTGACCGAACGCAGCGGTGGTAACGGCGCAGTGGCGGTTTTCATGGCTTGTTATGACTGTTTTTTTGTACAGCCTATGCCTCGGGCATCCAAGCAGCAAGCGCGTTACGCCGTGGGTCGATGTTTGATGTTATGGAGCAGCAACGATGTTACGCAGCAGCAACGATGTTACGCAGCAGGGCAGTCGCCCTAAAACAAAGTTAGGTGGCTCAAGTATGGGCATCATTCGCACATGTAGGCTCGGCCCTGACCAAGTCAAATCCATGCGGGCTGCTCTTGATCTTTTCGGTCGTGAGTTCGGAGACGTAGCCACCTACTCCCAACATCAGCCGGACTCCGATTACCTCGGGAACTTGCTCCGTAGTAAGACATTCATCGCGCTTGCTGCCTTCGACCAAGAAGCGGTTGTTGGCGCTCTCGCGGCTTACGTTCTGCCCAAGTTTGAGCAGCCGCGTAGTGAGATCTATATCTATGATCTCGCAGTCTCCGGAGAGCACCGGAGGCAGGGCATTGCCACCGCGCTCATCAATCTCCTCAAGCATGAGGCCAACGCGCTTGGTGCTTATGTGATCTACGTGCAAGCAGATTACGGTGACGATCCCGCAGTGGCTCTCTATACAAAGTTGGGCATACGGGAAGAAGTGATGCACTTTGATATCGACCCAAGTACCGCCACCTAACAATTCGTTCAAGCCGAGATCGGCTTCCCGGCCGCGGAGTTGTTCGGTAAATTGGACAACGGTCCGCGCGTTGTCCTTTTCCGCTGCATAACCCTGCTTCGGGGTCATTATAGCGATTTTTTCGGTATATCCATCCTTTTTCGCACGATATACAGGATTTTGCCAAAGGGTTCGTGTAGACTTTCCTTGGTGTATCCAACGGCGTCAGCCGGGCAGGATAGGTGAAGTAGGCCCACCCGCGAGCGGGTGTTCCTTCTTCACTGTCCCTTATTCGCACCTGGCGGTGCTCAACGGGAATCCTGCTCTGCGAGGCTGGCCGTAGGCCGGCCGATAATCTCATGACCAAAATCCCTTAACGTGAGTTTTCGTTCCACTGAGCGTCAGACCCCGTAGAAAAGATCAAAGGATCTTCTTGAGATCCTTTTTTTCTGCGCGTAATCTGCTGCTTGCAAACAAAAAAACCACCGCTACCAGCGGTGGTTTGTTTGCCGGATCAAGAGCTACCAACTCTTTTTCCGAAGGTAACTGGCTTCAGCAGAGCGCAGATACCAAATACTGTTCTTCTAGTGTAGCCGTAGTTAGGCCACCACTTCAAGAACTCTGTAGCACCGCCTACATACCTCGCTCTGCTAATCCTGTTACCAGTGGCTGCTGCCAGTGGCGATAAGTCGTGTCTTACCGGGTTGGACTCAAGACGATAGTTACCGGATAAGGCGCAGCGGTCGGGCTGAACGGGGGGTTCGTGCACACAGCCCAGCTTGGAGCGAACGACCTACACCGAACTGAGATACCTACAGCGTGAGCTATGAGAAAGCGCCACGCTTCCCGAAGGGAGAAAGGCGGACAGGCATCCGGTAAGCGGCAGGGTCGGAACAGGAGAGCGCACGAGGGAGCTTCCAGGGGGAAACGCCTGGTATCTTTATAGTCCTGTCGGGTTTCGCCACCTCTGACTTGAGCGTCGATTTTTGTGATGCTCGTCAGGGGGGCGGAGCCTATGGAAAAACGCCAGCAACGCGGCCGTGAAAGGCAGGCCGGTCCGTGGTGGCCACGGCCTCTAGGCCAGATCCAGCGGCATCTGGGTTAGTCGAGCGCGGGCCGCTTCCCATGTCTCACCAGGGCGAGCCTGTTTCGCGATCTCAGCATCTGAAATCTTCCCGGCCTTGCGCTTCGCTGGGGCCTTACCCACCGCCTTGGCGGGCTTCTTCGGTCCAAAACTGAACAACAGATGTGTGACCTTGCGCCCGGTCTTTCGCTGCGCCCACTCCACCTGTAGCGGGCTGTGCTCGTTGATCTGCGTCACGGCTGGATCAAGCACTCGCAACTTGAAGTCCTTGATCGAGGGATACCGGCCTTCCAGTTGAAACCACTTTCGCAGCTGGTCAATTTCTATTTCGCGCTGGCCGATGCTGTCCCATTGCATGAGCAGCTCGTAAAGCCTGATCGCGTGGGTGCTGTCCATCTTGGCCACGTCAGCCAAGGCGTATTTGGTGAACTGTTTGGTGAGTTCCGTCAGGTACGGCAGCATGTCTTTGGTGAACCTGAGTTCTACACGGCCCTCACCCTCCCGGTAGATGATTGTTTGCACCCAGCCGGTAATCATCACACTCGGTCTTTTCCCCTTGCCATTGGGCTCTTGGGTTAACCGGACTTCCCGCCGTTTCAGGCGCAGGGCCGCTTCTTTGAGCTGGTTGTAGGAAGATTCGATAGGGACACCCGCCATCGTCGCTATGTCCTCCGCCGTCACTGAATACATCACTTCATCGGTGACAGGCTCGCTCCTCTTCACCTGGCTAATACAGGCCAGAACGATCCGCTGTTCCTGAACACTGAGGCGATACGCGGCCTCGACCAGGGCATTGCTTTTGTAAACCATTGGGGGTGAGGCCACGTTCGACATTCCTTGTGTATAAGGGGACACTGTATCTGCGTCCCACAATACAACAAATCCGTCCCTTTACAACAACAAATCCGTCCCTTCTTAACAACAAATCCGTCCCTTAATGGCAACAAATCCGTCCCTTTTTAAACTCTACAGGCCACGGATTACGTGGCCTGTAGACGTCCTAAAAGGTTTAAAAGGGAAAAGGAAGAAAAGGGTGGAAACGCAAAAAACGCACCACTACGTGGCCCCGTTGGGGCCGCATTTGTGCCCCTGAAGGGGCGGGGGAGGCGTCTGGGCAATCCCCGTTTTACCAGTCCCCTATCGCCGCCTGAGAGGGCGCAGGAAGCGAGTAATCAGGGTATCGAGGCGGATTCACCCTTGGCGTCCAACCAGCGGCACCAGCGGCGCCTGAGAGGGGCGCGCCCAGCTGTCTAGGGCGGCGGATTTGTCCTACTCAGGAGAGCGTTCACCGACAAACAACAGATAAAACGAAAGGCCCAGTCTTTCGACTGAGCCTTTCGTTTTATTTGATGCCT**

**DNA sequence of plasmid pSEVA6BE-S**

TTAATTAATGACACCATGAATTCTTGACAGCTAGCTCAGTCCTAGGTATAATGCTAGCTGCCAAACGATTGCAGGCTGGTTTTAGAGCTAGAAATAGCAAGTTAAAATAAGGCTAGTCCGTTATCAACTTGAAAAAGTGGCACCGAGTCGGTGCTTTTTTTGAGGAGCTCGGTCACCGTTACATATCAAAGGGAAAACTGTCCATACCCATGGGGCATGGCGAATTATGACAACTTGACGGCTACATCATTCACTTTTTCTTCACAACCGGCACGGAACTCGCTCGGGCTGGCCCCGGTGCATTTTTTAAATACCCGCGAGAAGTAGAGTTGATCGTCAAAACCAACATTGCGACCGACGGTGGCGATAGGCATCCGGGTGGTGCTCAAAAGCAGCTTCGCCTGGCTGATACGTTGGTCCTCGCGCCAGCTTAAGACGCTAATCCCTAACTGCTGGCGGAAAAGATGTGACAGACGCGACGGCGACAAGCAAACATGCTGTGCGACGCTGGCGATATCAAAATTGCTGTCTGCCAGGTGATCGCTGATGTACTGACAAGCCTCGCGTACCCGATTATCCATCGGTGGATGGAGCGACTCGTTAATCGCTTCCATGCGCCGCAGTAACAATTGCTCAAGCAGATTTATCGCCAGCAGCTCCGAATAGCGCCCTTCCCCTTGCCCGGCGTTAATGATTTGCCCAAACAGGTCGCTGAAATGCGGCTGGTGCGCTTCATCCGGGCGAAAGAACCCCGTATTGGCAAATATTGACGGCCAGTTAAGCCATTCATGCCAGTAGGCGCGCGGACGAAAGTAAACCCACTGGTGATACCATTCGCGAGCCTCCGGATGACGACCGTAGTGATGAATCTCTCCTGGCGGGAACAGCAAAATATCACCCGGTCGGCAAACAAATTCTCGTCCCTGATTTTTCACCACCCCCTGACCGCGAATGGTGAGATTGAGAATATAACCTTTCATTCCCAGCGGTCGGTCGATAAAAAAATCGAGATAACCGTTGGCCTCAATCGGCGTTAAACCCGCCACCAGATGGGCATTAAACGAGTATCCCGGCAGCAGGGGATCATTTTGCGCTTCAGCCATACTTTTCATACTCCCGCCATTCAGAGAAGAAACCAATTGTCCATATTGCATCAGACATTGCCGTCACTGCGTCTTTTACTGGCTCTTCTCGCTAACCAAACCGGTAACCCCGCTTATTAAAAGCATTCTGTAACAAAGCGGGACCAAAGCCATGACAAAAACGCGTAACAAAAGTGTCTATAATCACGGCAGAAAAGTCCACATTGATTATTTGCACGGCGTCACACTTTGCTATGCCATAGCATTTTTATCCATAAGATTAGCGGATCCTACCTGACGCTTTTTATCGCAACTCTCTACTGTTTCTCCATCTTAAGGGCGATAGGAGGAATATACCATGAGCAGCGAAACCGGCCCGGTGGCCGTGGACCCGACCCTGCGTCGCCGCATCGAGCCGCACGAGTTCGAAGTGTTCTTCGACCCGCGCGAGCTGCGCAAGGAAACCTGCCTGCTGTACGAAATCAACTGGGGCGGCCGCCACAGCATCTGGCGCCACACCAGCCAGAACACCAACAAGCACGTGGAGGTGAACTTCATCGAAAAGTTCACCACCGAGCGCTACTTCTGCCCGAACACCCGCTGCAGCATCACCTGGTTCCTGAGCTGGAGCCCGTGCGGCGAATGCAGCCGCGCCATCACCGAGTTCCTGAGCCGCTACCCGCACGTGACCCTGTTCATCTACATCGCCCGCCTGTACCACCACGCCGACCCGCGCAACCGCCAGGGCCTGCGCGACCTGATCAGCAGCGGCGTGACCATCCAGATCATGACCGAGCAGGAAAGCGGCTACTGCTGGCGCAACTTCGTGAACTACAGCCCGAGCAACGAAGCCCACTGGCCGCGCTACCCGCACCTGTGGGTGCGCCTGTACGTGCTGGAGCTGTACTGCATCATCCTGGGCCTGCCGCCGTGCCTGAACATCCTGCGTCGCAAGCAGCCGCAGCTGACCTTCTTCACCATCGCCCTGCAGAGCTGCCACTACCAGCGCCTGCCGCCGCACATCCTGTGGGCCACCGGCCTGAAGAGCGGCAGCGAGACTCCCGGGACCTCAGAGTCCGCCACACCCGAAAGTGACAAGAAGTACAGCATCGGCCTGGCTATCGGCACCAACAGCGTGGGCTGGGCCGTGATCACCGACGAGTACAAGGTGCCGAGCAAGAAGTTCAAGGTGCTGGGCAACACCGACCGCCACAGCATCAAGAAGAACCTGATCGGCGCCCTGCTGTTCGACAGCGGCGAGACCGCCGAAGCCACCCGCCTGAAGCGCACCGCCCGCCGCCGCTACACCCGTCGCAAGAACCGCATCTGCTACCTGCAGGAGATCTTCAGCAACGAAATGGCCAAGGTGGACGACAGCTTCTTCCACCGCCTGGAGGAAAGCTTCCTGGTGGAGGAAGACAAGAAGCACGAGCGCCACCCGATCTTCGGCAACATCGTGGACGAGGTGGCCTACCACGAAAAGTACCCGACCATCTACCACCTGCGCAAGAAGCTGGTCGACAGCACCGACAAGGCCGACCTGCGCCTGATCTACCTGGCCCTGGCCCACATGATCAAGTTCCGCGGCCACTTCCTGATCGAAGGCGACCTGAACCCGGACAACAGCGACGTGGACAAGCTGTTCATCCAGCTGGTGCAGACCTACAACCAGCTGTTCGAGGAAAACCCGATCAACGCCAGCGGCGTGGACGCCAAGGCCATCCTGAGCGCCCGCCTGAGCAAGAGCCGCCGCCTGGAGAACCTGATCGCCCAGCTGCCGGGCGAAAAGAAGAACGGCCTGTTCGGCAACCTGATCGCCCTGAGCCTGGGCCTGACCCCGAACTTCAAGAGCAACTTCGACCTGGCCGAGGACGCCAAGCTGCAGCTGAGCAAGGACACCTACGACGACGACCTGGACAACCTGCTGGCCCAGATCGGCGACCAGTACGCCGACCTGTTCCTGGCCGCCAAGAACCTGAGCGACGCCATCCTGCTGAGCGACATCCTGCGCGTGAACACCGAGATCACCAAGGCCCCGCTGAGCGCCAGCATGATCAAGCGCTACGACGAACACCACCAGGACCTGACCCTGCTGAAGGCCCTGGTGCGCCAGCAGCTGCCGGAGAAGTACAAGGAAATCTTCTTCGACCAGAGCAAGAACGGCTACGCCGGCTACATCGACGGTGGCGCCAGCCAGGAAGAGTTCTACAAGTTCATCAAGCCGATCCTGGAGAAGATGGACGGCACCGAGGAACTGCTGGTGAAGCTGAACCGCGAAGACCTGCTGCGCAAGCAGCGCACCTTCGACAACGGCAGCATCCCGCACCAGATCCACCTGGGCGAGCTGCACGCCATCCTGCGTCGCCAGGAAGACTTCTACCCGTTCCTGAAGGACAACCGCGAGAAGATCGAAAAGATCCTGACCTTCCGCATCCCGTACTACGTGGGCCCGCTGGCCCGCGGCAACAGCCGCTTCGCCTGGATGACCCGCAAGAGCGAGGAAACCATCACCCCGTGGAACTTCGAGGAAGTGGTGGACAAGGGCGCCAGCGCCCAGAGCTTCATCGAGCGCATGACCAACTTCGACAAGAACCTGCCGAACGAAAAGGTGCTGCCGAAGCACAGCCTGCTGTACGAGTACTTCACCGTGTACAACGAACTGACCAAGGTGAAGTACGTGACCGAGGGCATGCGCAAGCCGGCCTTCCTGAGCGGCGAACAGAAGAAGGCCATCGTGGACCTGCTGTTCAAGACCAACCGCAAGGTGACCGTGAAGCAGCTGAAGGAAGACTACTTCAAGAAGATCGAATGCTTCGACAGCGTGGAGATCAGCGGCGTGGAAGACCGCTTCAACGCCAGCCTGGGCACCTACCACGACCTGCTGAAGATCATCAAGGACAAGGACTTCCTGGACAACGAGGAAAACGAGGACATCCTGGAAGACATCGTGCTGACCCTGACCCTGTTCGAGGACCGCGAAATGATCGAGGAACGCCTGAAGACCTACGCCCACCTGTTCGACGACAAGGTGATGAAGCAGCTGAAGCGCCGCCGCTACACCGGCTGGGGCCGCCTGAGCCGCAAGCTGATCAACGGCATCCGCGACAAGCAGAGCGGCAAGACCATCCTGGACTTCCTGAAGAGCGACGGCTTCGCCAACCGCAACTTCATGCAGCTGATCCACGACGACAGCCTGACCTTCAAGGAAGACATCCAGAAGGCCCAAGTGAGCGGCCAGGGCGACAGCCTGCACGAACACATCGCCAACCTGGCCGGCAGCCCGGCCATCAAGAAGGGCATCCTGCAGACCGTGAAGGTGGTGGACGAGCTGGTGAAGGTGATGGGCCGCCACAAGCCGGAAAACATCGTGATCGAGATGGCCCGCGAAAACCAGACCACCCAGAAGGGCCAGAAGAACAGCCGCGAGCGCATGAAGCGCATCGAGGAAGGCATCAAGGAACTGGGCAGCCAGATCCTGAAGGAGCACCCGGTGGAAAACACCCAGCTGCAGAACGAGAAGCTGTACCTGTACTACCTGCAGAACGGCCGCGACATGTACGTGGACCAGGAACTGGACATCAACCGCCTGAGCGACTACGACGTGGACCACATCGTGCCGCAGAGCTTCCTGGCCGACGACAGCATCGACAACAAGGTGCTGACCCGCAGCGACAAGAACCGCGGCAAGAGCGACAACGTGCCGAGCGAGGAAGTGGTGAAGAAGATGAAGAACTACTGGCGCCAGCTGCTGAACGCCAAGCTGATCACCCAGCGCAAGTTCGACAACCTGACCAAGGCCGAGCGCGGCGGCCTGAGCGAACTGGACAAGGCCGGCTTCATCAAGCGCCAGCTGGTCGAGACCCGCCAGATCACCAAGCACGTGGCCCAGATCCTGGACAGCCGCATGAACACCAAGTACGACGAGAACGACAAGCTGATCCGCGAAGTGAAGGTGATCACCCTGAAGAGCAAGCTGGTCAGCGACTTCCGCAAGGACTTCCAGTTCTACAAGGTGCGCGAAATCAACAACTACCACCACGCCCACGACGCCTACCTGAACGCCGTGGTGGGCACCGCCCTGATCAAGAAGTACCCGGCCCTGGAGAGCGAATTCGTGTACGGCGACTACAAGGTGTACGACGTGCGCAAGATGATCGCCAAGAGCGAGCAGGAAATCGGCAAGGCCACCGCCAAGTACTTCTTCTACAGCAACATCATGAACTTCTTCAAGACCGAGATCACCCTGGCCAACGGCGAAATCCGCAAGGCCCCGCTGATCGAGACCAACGGCGAGACCGGCGAAATCGTGTGGGACAAGGGCCGCGACTTCGCCACCGTGCGCAAGGTGCTGAGCATGCCGCAGGTGAACATCGTGAAGAAGACCGAGGTGCAGACCGGCGGCTTCAGCAAGGAAAGCATCCTGCCGAAGCGCAACAGCGACAAGCTGATCGCCCGCAAGAAGGACTGGGACCCGAAGAAGTACGGCGGCTTCGACAGCCCGACCGTGGCCTACAGCGTGCTGGTCGTGGCCAAGGTGGAGAAGGGCAAGAGCAAGAAGCTGAAGAGCGTGAAGGAACTGCTGGGCATCACCATCATGGAGCGCAGCAGCTTCGAAAAGAACCCGATCGACTTCCTGGAGGCCAAGGGCTACAAGGAAGTGAAGAAGGACCTGATCATCAAGCTGCCGAAGTACAGCCTGTTCGAGCTGGAAAACGGCCGCAAGCGCATGCTGGCCAGCGCCGGCGAGCTGCAGAAGGGCAACGAACTGGCCCTGCCGAGCAAGTACGTGAACTTCCTGTACCTGGCCTCGCACTACGAGAAGCTGAAGGGCAGCCCGGAGGACAACGAACAGAAGCAGCTGTTCGTGGAGCAGCACAAGCACTACCTGGACGAGATCATCGAACAGATCAGCGAATTCAGCAAGCGCGTGATCCTGGCCGACGCCAACCTGGACAAGGTGCTGAGCGCCTACAACAAGCACCGCGACAAGCCGATCCGCGAGCAGGCCGAAAACATCATCCACCTGTTCACCCTGACCAACCTGGGCGCCCCGGCCGCCTTCAAGTACTTCGACACCACCATCGACCGCAAGCGCTACACCAGCACCAAGGAAGTGCTGGACGCCACCCTGATCCACCAGAGCATCACCGGCCTGTACGAAACCCGCATCGACCTGAGCCAGCTGGGCGGCGACTCTGGTGGTTCTACTAATCTGTCAGATATTATTGAAAAGGAAACCGGTAAGCAACTGGTTATCCAGGAATCCATCCTCATGCTCCCAGAGGAGGTGGAAGAAGTCATTGGGAACAAGCCGGAAAGCGATATACTCGTGCACACCGCCTACGACGAGAGCACCGACGAGAATGTCATGCTTCTGACTAGCGACGCCCCTGAATACAAGCCTTGGGCTCTGGTCATACAGGATAGCAACGGTGAGAACAAGATTAAGATGCTCTCTGGTGGTTCTTGAACTAGTCTTGGACTCCTGTTGATAGATCCAGTAATGACCTCAGAACTCCATCTGGATTTGTTCAGAACGCTCGGTTGCCGCCGGGCGTTTTTTATTGGTGAGAATCCAGCATTTTCTTTTGCGTTTTTATTTGTTAACTGTTAATTGTCCTTGTTCAAGGATGCTGTCTTTGACAACAGATGTTTTCTTGCCTTTGATGTTCAGCAGGAAGCTTGGCGCAAACGTTGATTGTTTGTCTGCGTAGAATCCTCTGTTTGTCATATAGCTTGTAATCACGACATTGTTTCCTTTCGCTTGAGGTACAGCGAAGTGTGAGTAAGTAAAGGTTACATCGTTAGGATCAAGATCCATTTTTAACACAAGGCCAGTTTTGTTCAGCGGCTTGTATGGGCCAGTTAAAGAATTAGAAACATAACCAAGCATGTAAATATCGTTAGACGTAATGCCGTCAATCGTCATTTTTGATCCGCGGGAGTCAGTGAACAGGTACCATTTGCCGTTCATTTTAAAGACGTTCGCGCGTTCAATTTCATCTGTTACTGTGTTAGATGCAATCAGCGGTTTCATCACTTTTTTCAGTGTGTAATCATCGTTTAGCTCAATCATACCGAGAGCGCCGTTTGCTAACTCAGCCGTGCGTTTTTTATCGCTTTGCAGAAGTTTTTGACTTTCTTGACGGAAGAATGATGTGCTTTTGCCATAGTATGCTTTGTTAAATAAAGATTCTTCGCCTTGGTAGCCATCTTCAGTTCCAGTGTTTGCTTCAAATACTAAGTATTTGTGGCCTTTATCTTCTACGTAGTGAGGATCTCTCAGCGTATGGTTGTCGCCTGAGCTGTAGTTGCCTTCATCGATGAACTGCTGTACATTTTGATACGTTTTTCCGTCACCGTCAAAGATTGATTTATAATCCTCTACACCGTTGATGTTCAAAGAGCTGTCTGATGCTGATACGTTAACTTGTGCAGTTGTCAGTGTTTGTTTGCCGTAATGTTTACCGGAGAAATCAGTGTAGAATAAACGGATTTTTCCGTCAGATGTAAATGTGGCTGAACCTGACCATTCTTGTGTTTGGTCTTTTAGGATAGAATCATTTGCATCGAATTTGTCGCTGTCTTTAAAGACGCGGCCAGCGTTTTTCCAGCTGTCAATAGAAGTTTCGCCGACTTTTTGATAGAACATGTAAATCGATGTGTCATCCGCATTTTTAGGATCTCCGGCTAATGCAAAGACGATGTGGTAGCCGTGATAGTTTGCGACAGTGCCGTCAGCGTTTTGTAATGGCCAGCTGTCCCAAACGTCCAGGCCTTTTGCAGAAGAGATATTTTTAATTGTGGACGAATCGAATTCAGGAACTTGATATTTTTCATTTTTTTGCTGTTCAGGGATTTGCAGCATATCATGGCGTGTAATATGGGAAATGCCGTATGTTTCCTTATATGGCTTTTGGTTCGTTTCTTTCGCAAACGCTTGAGTTGCGCCTCCTGCCAGCAGTGCGGTAGTAAAGGTTAATACTGTTGCTTGTTTTGCAAACTTTTTGATGTTCATCGTTCATGTCTCCTTTTTTATGTACTGTGTTAGCGGTCTGCTTCTTCCAGCCCTCCTGTTTGAAGATGGCAAGTTAGTTACGCACAATAAAAAAAGACCTAAAATATGTAAGGGGTGACGCCAAAGTATACACTTTGCCCTTTACACATTTTAGGTCTTGCCTGCTTTATCAGTAACAAACCCGCGCGATTTACTTTTCGACCTCATTCTATTAGACTCTCGTTTGGATTGCAACTGGTCTATTTTCCTCTTTTGTTTGATGGGTCCCCAATAATTACGATTTAAATTTGACATAAGCCTGTTCGGTTCGTAAACTGTAATGCAAGTAGCGTATGCGCTCACGCAACTGGTCCAGAACCTTGACCGAACGCAGCGGTGGTAACGGCGCAGTGGCGGTTTTCATGGCTTGTTATGACTGTTTTTTTGTACAGCCTATGCCTCGGGCATCCAAGCAGCAAGCGCGTTACGCCGTGGGTCGATGTTTGATGTTATGGAGCAGCAACGATGTTACGCAGCAGCAACGATGTTACGCAGCAGGGCAGTCGCCCTAAAACAAAGTTAGGTGGCTCAAGTATGGGCATCATTCGCACATGTAGGCTCGGCCCTGACCAAGTCAAATCCATGCGGGCTGCTCTTGATCTTTTCGGTCGTGAGTTCGGAGACGTAGCCACCTACTCCCAACATCAGCCGGACTCCGATTACCTCGGGAACTTGCTCCGTAGTAAGACATTCATCGCGCTTGCTGCCTTCGACCAAGAAGCGGTTGTTGGCGCTCTCGCGGCTTACGTTCTGCCCAAGTTTGAGCAGCCGCGTAGTGAGATCTATATCTATGATCTCGCAGTCTCCGGAGAGCACCGGAGGCAGGGCATTGCCACCGCGCTCATCAATCTCCTCAAGCATGAGGCCAACGCGCTTGGTGCTTATGTGATCTACGTGCAAGCAGATTACGGTGACGATCCCGCAGTGGCTCTCTATACAAAGTTGGGCATACGGGAAGAAGTGATGCACTTTGATATCGACCCAAGTACCGCCACCTAACAATTCGTTCAAGCCGAGATCGGCTTCCCGGCCGCGGAGTTGTTCGGTAAATTGGACAACGGTCCGCGCGTTGTCCTTTTCCGCTGCATAACCCTGCTTCGGGGTCATTATAGCGATTTTTTCGGTATATCCATCCTTTTTCGCACGATATACAGGATTTTGCCAAAGGGTTCGTGTAGACTTTCCTTGGTGTATCCAACGGCGTCAGCCGGGCAGGATAGGTGAAGTAGGCCCACCCGCGAGCGGGTGTTCCTTCTTCACTGTCCCTTATTCGCACCTGGCGGTGCTCAACGGGAATCCTGCTCTGCGAGGCTGGCCGTAGGCCGGCCGATAATCTCATGACCAAAATCCCTTAACGTGAGTTTTCGTTCCACTGAGCGTCAGACCCCGTAGAAAAGATCAAAGGATCTTCTTGAGATCCTTTTTTTCTGCGCGTAATCTGCTGCTTGCAAACAAAAAAACCACCGCTACCAGCGGTGGTTTGTTTGCCGGATCAAGAGCTACCAACTCTTTTTCCGAAGGTAACTGGCTTCAGCAGAGCGCAGATACCAAATACTGTTCTTCTAGTGTAGCCGTAGTTAGGCCACCACTTCAAGAACTCTGTAGCACCGCCTACATACCTCGCTCTGCTAATCCTGTTACCAGTGGCTGCTGCCAGTGGCGATAAGTCGTGTCTTACCGGGTTGGACTCAAGACGATAGTTACCGGATAAGGCGCAGCGGTCGGGCTGAACGGGGGGTTCGTGCACACAGCCCAGCTTGGAGCGAACGACCTACACCGAACTGAGATACCTACAGCGTGAGCTATGAGAAAGCGCCACGCTTCCCGAAGGGAGAAAGGCGGACAGGCATCCGGTAAGCGGCAGGGTCGGAACAGGAGAGCGCACGAGGGAGCTTCCAGGGGGAAACGCCTGGTATCTTTATAGTCCTGTCGGGTTTCGCCACCTCTGACTTGAGCGTCGATTTTTGTGATGCTCGTCAGGGGGGCGGAGCCTATGGAAAAACGCCAGCAACGCGGCCGTGAAAGGCAGGCCGGTCCGTGGTGGCCACGGCCTCTAGGCCAGATCCAGCGGCATCTGGGTTAGTCGAGCGCGGGCCGCTTCCCATGTCTCACCAGGGCGAGCCTGTTTCGCGATCTCAGCATCTGAAATCTTCCCGGCCTTGCGCTTCGCTGGGGCCTTACCCACCGCCTTGGCGGGCTTCTTCGGTCCAAAACTGAACAACAGATGTGTGACCTTGCGCCCGGTCTTTCGCTGCGCCCACTCCACCTGTAGCGGGCTGTGCTCGTTGATCTGCGTCACGGCTGGATCAAGCACTCGCAACTTGAAGTCCTTGATCGAGGGATACCGGCCTTCCAGTTGAAACCACTTTCGCAGCTGGTCAATTTCTATTTCGCGCTGGCCGATGCTGTCCCATTGCATGAGCAGCTCGTAAAGCCTGATCGCGTGGGTGCTGTCCATCTTGGCCACGTCAGCCAAGGCGTATTTGGTGAACTGTTTGGTGAGTTCCGTCAGGTACGGCAGCATGTCTTTGGTGAACCTGAGTTCTACACGGCCCTCACCCTCCCGGTAGATGATTGTTTGCACCCAGCCGGTAATCATCACACTCGGTCTTTTCCCCTTGCCATTGGGCTCTTGGGTTAACCGGACTTCCCGCCGTTTCAGGCGCAGGGCCGCTTCTTTGAGCTGGTTGTAGGAAGATTCGATAGGGACACCCGCCATCGTCGCTATGTCCTCCGCCGTCACTGAATACATCACTTCATCGGTGACAGGCTCGCTCCTCTTCACCTGGCTAATACAGGCCAGAACGATCCGCTGTTCCTGAACACTGAGGCGATACGCGGCCTCGACCAGGGCATTGCTTTTGTAAACCATTGGGGGTGAGGCCACGTTCGACATTCCTTGTGTATAAGGGGACACTGTATCTGCGTCCCACAATACAACAAATCCGTCCCTTTACAACAACAAATCCGTCCCTTCTTAACAACAAATCCGTCCCTTAATGGCAACAAATCCGTCCCTTTTTAAACTCTACAGGCCACGGATTACGTGGCCTGTAGACGTCCTAAAAGGTTTAAAAGGGAAAAGGAAGAAAAGGGTGGAAACGCAAAAAACGCACCACTACGTGGCCCCGTTGGGGCCGCATTTGTGCCCCTGAAGGGGCGGGGGAGGCGTCTGGGCAATCCCCGTTTTACCAGTCCCCTATCGCCGCCTGAGAGGGCGCAGGAAGCGAGTAATCAGGGTATCGAGGCGGATTCACCCTTGGCGTCCAACCAGCGGCACCAGCGGCGCCTGAGAGGGGCGCGCCCAGCTGTCTAGGGCGGCGGATTTGTCCTACTCAGGAGAGCGTTCACCGACAAACAACAGATAAAACGAAAGGCCCAGTCTTTCGACTGAGCCTTTCGTTTTATTTGATGCCT

**DNA sequence of plasmid pSEVA6BE-PobA-TrpE**

**
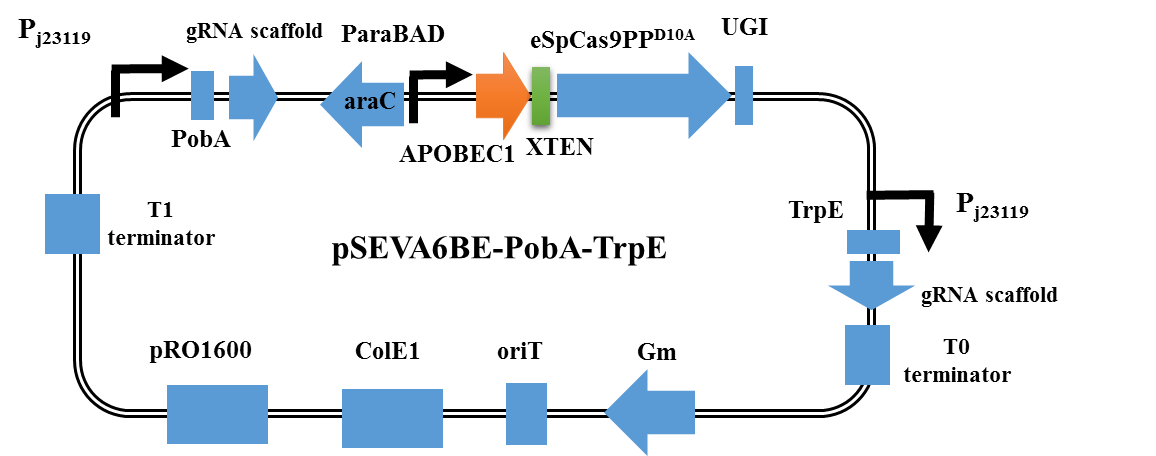
**

TTAATTAATGACACCATGAATTCTTGACAGCTAGCTCAGTCCTAGGTATAATGCTAGCAAAACTCAGGTTGCAATTATGTTTTAGAGCTAGAAATAGCAAGTTAAAATAAGGCTAGTCCGTTATCAACTTGAAAAAGTGGCACCGAGTCGGTGCTTTTTTTGAGGAGCTCGGTCACCGTTACATATCAAAGGGAAAACTGTCCATACCCATGGGGCATGGCGAATTATGACAACTTGACGGCTACATCATTCACTTTTTCTTCACAACCGGCACGGAACTCGCTCGGGCTGGCCCCGGTGCATTTTTTAAATACCCGCGAGAAGTAGAGTTGATCGTCAAAACCAACATTGCGACCGACGGTGGCGATAGGCATCCGGGTGGTGCTCAAAAGCAGCTTCGCCTGGCTGATACGTTGGTCCTCGCGCCAGCTTAAGACGCTAATCCCTAACTGCTGGCGGAAAAGATGTGACAGACGCGACGGCGACAAGCAAACATGCTGTGCGACGCTGGCGATATCAAAATTGCTGTCTGCCAGGTGATCGCTGATGTACTGACAAGCCTCGCGTACCCGATTATCCATCGGTGGATGGAGCGACTCGTTAATCGCTTCCATGCGCCGCAGTAACAATTGCTCAAGCAGATTTATCGCCAGCAGCTCCGAATAGCGCCCTTCCCCTTGCCCGGCGTTAATGATTTGCCCAAACAGGTCGCTGAAATGCGGCTGGTGCGCTTCATCCGGGCGAAAGAACCCCGTATTGGCAAATATTGACGGCCAGTTAAGCCATTCATGCCAGTAGGCGCGCGGACGAAAGTAAACCCACTGGTGATACCATTCGCGAGCCTCCGGATGACGACCGTAGTGATGAATCTCTCCTGGCGGGAACAGCAAAATATCACCCGGTCGGCAAACAAATTCTCGTCCCTGATTTTTCACCACCCCCTGACCGCGAATGGTGAGATTGAGAATATAACCTTTCATTCCCAGCGGTCGGTCGATAAAAAAATCGAGATAACCGTTGGCCTCAATCGGCGTTAAACCCGCCACCAGATGGGCATTAAACGAGTATCCCGGCAGCAGGGGATCATTTTGCGCTTCAGCCATACTTTTCATACTCCCGCCATTCAGAGAAGAAACCAATTGTCCATATTGCATCAGACATTGCCGTCACTGCGTCTTTTACTGGCTCTTCTCGCTAACCAAACCGGTAACCCCGCTTATTAAAAGCATTCTGTAACAAAGCGGGACCAAAGCCATGACAAAAACGCGTAACAAAAGTGTCTATAATCACGGCAGAAAAGTCCACATTGATTATTTGCACGGCGTCACACTTTGCTATGCCATAGCATTTTTATCCATAAGATTAGCGGATCCTACCTGACGCTTTTTATCGCAACTCTCTACTGTTTCTCCATCTTAAGGGCGATAGGAGGAATATACCATGAGCAGCGAAACCGGCCCGGTGGCCGTGGACCCGACCCTGCGTCGCCGCATCGAGCCGCACGAGTTCGAAGTGTTCTTCGACCCGCGCGAGCTGCGCAAGGAAACCTGCCTGCTGTACGAAATCAACTGGGGCGGCCGCCACAGCATCTGGCGCCACACCAGCCAGAACACCAACAAGCACGTGGAGGTGAACTTCATCGAAAAGTTCACCACCGAGCGCTACTTCTGCCCGAACACCCGCTGCAGCATCACCTGGTTCCTGAGCTGGAGCCCGTGCGGCGAATGCAGCCGCGCCATCACCGAGTTCCTGAGCCGCTACCCGCACGTGACCCTGTTCATCTACATCGCCCGCCTGTACCACCACGCCGACCCGCGCAACCGCCAGGGCCTGCGCGACCTGATCAGCAGCGGCGTGACCATCCAGATCATGACCGAGCAGGAAAGCGGCTACTGCTGGCGCAACTTCGTGAACTACAGCCCGAGCAACGAAGCCCACTGGCCGCGCTACCCGCACCTGTGGGTGCGCCTGTACGTGCTGGAGCTGTACTGCATCATCCTGGGCCTGCCGCCGTGCCTGAACATCCTGCGTCGCAAGCAGCCGCAGCTGACCTTCTTCACCATCGCCCTGCAGAGCTGCCACTACCAGCGCCTGCCGCCGCACATCCTGTGGGCCACCGGCCTGAAGAGCGGCAGCGAGACTCCCGGGACCTCAGAGTCCGCCACACCCGAAAGTGACAAGAAGTACAGCATCGGCCTGGCTATCGGCACCAACAGCGTGGGCTGGGCCGTGATCACCGACGAGTACAAGGTGCCGAGCAAGAAGTTCAAGGTGCTGGGCAACACCGACCGCCACAGCATCAAGAAGAACCTGATCGGCGCCCTGCTGTTCGACAGCGGCGAGACCGCCGAAGCCACCCGCCTGAAGCGCACCGCCCGCCGCCGCTACACCCGTCGCAAGAACCGCATCTGCTACCTGCAGGAGATCTTCAGCAACGAAATGGCCAAGGTGGACGACAGCTTCTTCCACCGCCTGGAGGAAAGCTTCCTGGTGGAGGAAGACAAGAAGCACGAGCGCCACCCGATCTTCGGCAACATCGTGGACGAGGTGGCCTACCACGAAAAGTACCCGACCATCTACCACCTGCGCAAGAAGCTGGTCGACAGCACCGACAAGGCCGACCTGCGCCTGATCTACCTGGCCCTGGCCCACATGATCAAGTTCCGCGGCCACTTCCTGATCGAAGGCGACCTGAACCCGGACAACAGCGACGTGGACAAGCTGTTCATCCAGCTGGTGCAGACCTACAACCAGCTGTTCGAGGAAAACCCGATCAACGCCAGCGGCGTGGACGCCAAGGCCATCCTGAGCGCCCGCCTGAGCAAGAGCCGCCGCCTGGAGAACCTGATCGCCCAGCTGCCGGGCGAAAAGAAGAACGGCCTGTTCGGCAACCTGATCGCCCTGAGCCTGGGCCTGACCCCGAACTTCAAGAGCAACTTCGACCTGGCCGAGGACGCCAAGCTGCAGCTGAGCAAGGACACCTACGACGACGACCTGGACAACCTGCTGGCCCAGATCGGCGACCAGTACGCCGACCTGTTCCTGGCCGCCAAGAACCTGAGCGACGCCATCCTGCTGAGCGACATCCTGCGCGTGAACACCGAGATCACCAAGGCCCCGCTGAGCGCCAGCATGATCAAGCGCTACGACGAACACCACCAGGACCTGACCCTGCTGAAGGCCCTGGTGCGCCAGCAGCTGCCGGAGAAGTACAAGGAAATCTTCTTCGACCAGAGCAAGAACGGCTACGCCGGCTACATCGACGGTGGCGCCAGCCAGGAAGAGTTCTACAAGTTCATCAAGCCGATCCTGGAGAAGATGGACGGCACCGAGGAACTGCTGGTGAAGCTGAACCGCGAAGACCTGCTGCGCAAGCAGCGCACCTTCGACAACGGCAGCATCCCGCACCAGATCCACCTGGGCGAGCTGCACGCCATCCTGCGTCGCCAGGAAGACTTCTACCCGTTCCTGAAGGACAACCGCGAGAAGATCGAAAAGATCCTGACCTTCCGCATCCCGTACTACGTGGGCCCGCTGGCCCGCGGCAACAGCCGCTTCGCCTGGATGACCCGCAAGAGCGAGGAAACCATCACCCCGTGGAACTTCGAGGAAGTGGTGGACAAGGGCGCCAGCGCCCAGAGCTTCATCGAGCGCATGACCAACTTCGACAAGAACCTGCCGAACGAAAAGGTGCTGCCGAAGCACAGCCTGCTGTACGAGTACTTCACCGTGTACAACGAACTGACCAAGGTGAAGTACGTGACCGAGGGCATGCGCAAGCCGGCCTTCCTGAGCGGCGAACAGAAGAAGGCCATCGTGGACCTGCTGTTCAAGACCAACCGCAAGGTGACCGTGAAGCAGCTGAAGGAAGACTACTTCAAGAAGATCGAATGCTTCGACAGCGTGGAGATCAGCGGCGTGGAAGACCGCTTCAACGCCAGCCTGGGCACCTACCACGACCTGCTGAAGATCATCAAGGACAAGGACTTCCTGGACAACGAGGAAAACGAGGACATCCTGGAAGACATCGTGCTGACCCTGACCCTGTTCGAGGACCGCGAAATGATCGAGGAACGCCTGAAGACCTACGCCCACCTGTTCGACGACAAGGTGATGAAGCAGCTGAAGCGCCGCCGCTACACCGGCTGGGGCCGCCTGAGCCGCAAGCTGATCAACGGCATCCGCGACAAGCAGAGCGGCAAGACCATCCTGGACTTCCTGAAGAGCGACGGCTTCGCCAACCGCAACTTCATGCAGCTGATCCACGACGACAGCCTGACCTTCAAGGAAGACATCCAGAAGGCCCAAGTGAGCGGCCAGGGCGACAGCCTGCACGAACACATCGCCAACCTGGCCGGCAGCCCGGCCATCAAGAAGGGCATCCTGCAGACCGTGAAGGTGGTGGACGAGCTGGTGAAGGTGATGGGCCGCCACAAGCCGGAAAACATCGTGATCGAGATGGCCCGCGAAAACCAGACCACCCAGAAGGGCCAGAAGAACAGCCGCGAGCGCATGAAGCGCATCGAGGAAGGCATCAAGGAACTGGGCAGCCAGATCCTGAAGGAGCACCCGGTGGAAAACACCCAGCTGCAGAACGAGAAGCTGTACCTGTACTACCTGCAGAACGGCCGCGACATGTACGTGGACCAGGAACTGGACATCAACCGCCTGAGCGACTACGACGTGGACCACATCGTGCCGCAGAGCTTCCTGGCCGACGACAGCATCGACAACAAGGTGCTGACCCGCAGCGACAAGAACCGCGGCAAGAGCGACAACGTGCCGAGCGAGGAAGTGGTGAAGAAGATGAAGAACTACTGGCGCCAGCTGCTGAACGCCAAGCTGATCACCCAGCGCAAGTTCGACAACCTGACCAAGGCCGAGCGCGGCGGCCTGAGCGAACTGGACAAGGCCGGCTTCATCAAGCGCCAGCTGGTCGAGACCCGCCAGATCACCAAGCACGTGGCCCAGATCCTGGACAGCCGCATGAACACCAAGTACGACGAGAACGACAAGCTGATCCGCGAAGTGAAGGTGATCACCCTGAAGAGCAAGCTGGTCAGCGACTTCCGCAAGGACTTCCAGTTCTACAAGGTGCGCGAAATCAACAACTACCACCACGCCCACGACGCCTACCTGAACGCCGTGGTGGGCACCGCCCTGATCAAGAAGTACCCGGCCCTGGAGAGCGAATTCGTGTACGGCGACTACAAGGTGTACGACGTGCGCAAGATGATCGCCAAGAGCGAGCAGGAAATCGGCAAGGCCACCGCCAAGTACTTCTTCTACAGCAACATCATGAACTTCTTCAAGACCGAGATCACCCTGGCCAACGGCGAAATCCGCAAGGCCCCGCTGATCGAGACCAACGGCGAGACCGGCGAAATCGTGTGGGACAAGGGCCGCGACTTCGCCACCGTGCGCAAGGTGCTGAGCATGCCGCAGGTGAACATCGTGAAGAAGACCGAGGTGCAGACCGGCGGCTTCAGCAAGGAAAGCATCCTGCCGAAGCGCAACAGCGACAAGCTGATCGCCCGCAAGAAGGACTGGGACCCGAAGAAGTACGGCGGCTTCGACAGCCCGACCGTGGCCTACAGCGTGCTGGTCGTGGCCAAGGTGGAGAAGGGCAAGAGCAAGAAGCTGAAGAGCGTGAAGGAACTGCTGGGCATCACCATCATGGAGCGCAGCAGCTTCGAAAAGAACCCGATCGACTTCCTGGAGGCCAAGGGCTACAAGGAAGTGAAGAAGGACCTGATCATCAAGCTGCCGAAGTACAGCCTGTTCGAGCTGGAAAACGGCCGCAAGCGCATGCTGGCCAGCGCCGGCGAGCTGCAGAAGGGCAACGAACTGGCCCTGCCGAGCAAGTACGTGAACTTCCTGTACCTGGCCTCGCACTACGAGAAGCTGAAGGGCAGCCCGGAGGACAACGAACAGAAGCAGCTGTTCGTGGAGCAGCACAAGCACTACCTGGACGAGATCATCGAACAGATCAGCGAATTCAGCAAGCGCGTGATCCTGGCCGACGCCAACCTGGACAAGGTGCTGAGCGCCTACAACAAGCACCGCGACAAGCCGATCCGCGAGCAGGCCGAAAACATCATCCACCTGTTCACCCTGACCAACCTGGGCGCCCCGGCCGCCTTCAAGTACTTCGACACCACCATCGACCGCAAGCGCTACACCAGCACCAAGGAAGTGCTGGACGCCACCCTGATCCACCAGAGCATCACCGGCCTGTACGAAACCCGCATCGACCTGAGCCAGCTGGGCGGCGACTCTGGTGGTTCTACTAATCTGTCAGATATTATTGAAAAGGAAACCGGTAAGCAACTGGTTATCCAGGAATCCATCCTCATGCTCCCAGAGGAGGTGGAAGAAGTCATTGGGAACAAGCCGGAAAGCGATATACTCGTGCACACCGCCTACGACGAGAGCACCGACGAGAATGTCATGCTTCTGACTAGCGACGCCCCTGAATACAAGCCTTGGGCTCTGGTCATACAGGATAGCAACGGTGAGAACAAGATTAAGATGCTCTCTGGTGGTTCTTGAACTAGTCTTGGACTCCTGTTGATAGATCCAGTAATGACCTCAGAACTCCATCTGGATTTGTTCAGAACGCTCGGTTGCCGCCGGGCGTTTTTTATTGGTGAGAATCCAGGGGTCCCCAATAATTACGATTTAAATCGCCATATGGCGTTGACAGCTAGCTCAGTCCTAGGTATAATGCTAGCCAGGGCCAGGCACGTCTGCAGTTTTAGAGCTAGAAATAGCAAGTTAAAATAAGGCTAGTCCGTTATCAACTTGAAAAAGTGGCACCGAGTCGGTGCTTTTTTTGAGGAGCTCGGTCACCGTTACATATCAAAGGGAAAACTGTCCATACCCATGGGGCATGGCGAATTATGACAACTTGACGGCTACATCATTCACTTTTTCTTCACAACCGGCACGGAACTCGCTCGGGCTGGCCCCGGTGCATTTTTTAAATACCCGCGAGAAGTAGAGTTGATCGTCAAAACCAACATTGCGACCGACGGTGGCGATAGGCATCCGGGTGGTGCTCAAAAGCAGCTTCGCCTGGCTGATACGTTGGTCCTCGCGCCAGCTTAAGACGCTAATCCCTAACTGCTGGCGGAAAAGATGTGACAGACGCGACGGCGACAAGCAAACATGCTGTGCGACGCTGGCGATATCAAAATTGCTGTCTGCCAGGTGATCGCTGATGTACTGACAAGCCTCGCGTACCCGATTATCCATCGGTGGATGGAGCGACTCGTTAATCGCTTCCATGCGCCGCAGTAACAATTGCTCAAGCAGATTTATCGCCAGCAGCTCCGAATAGCGCCCTTCCCCTTGCCCGGCGTTAATGATTTGCCCAAACAGGTCGCTGAAATGCGGCTGGTGCGCTTCATCCGGGCGAAAGAACCCCGTATTGGCAAATATTGACGGCCAGTTAAGCCATTCATGCCAGTAGGCGCGCGGACGAAAGTAAACCCACTGGTGATACCATTCGCGAGCCTCCGGATGACGACCGTAGTGATGAATCTCTCCTGGCGGGAACAGCAAAATATCACCCGGTCGGCAAACAAATTCTCGTCCCTGATTTTTCACCACCCCCTGACCGCGAATGGTGAGATTGAGAATATAACCTTTCATTCCCAGCGGTCGGTCGATAAAAAAATCGAGATAACCGTTGGCCCCGCTCGAGCGGTTGACATAAGCCTGTTCGGTTCGTAAACTGTAATGCAAGTAGCGTATGCGCTCACGCAACTGGTCCAGAACCTTGACCGAACGCAGCGGTGGTAACGGCGCAGTGGCGGTTTTCATGGCTTGTTATGACTGTTTTTTTGTACAGCCTATGCCTCGGGCATCCAAGCAGCAAGCGCGTTACGCCGTGGGTCGATGTTTGATGTTATGGAGCAGCAACGATGTTACGCAGCAGCAACGATGTTACGCAGCAGGGCAGTCGCCCTAAAACAAAGTTAGGTGGCTCAAGTATGGGCATCATTCGCACATGTAGGCTCGGCCCTGACCAAGTCAAATCCATGCGGGCTGCTCTTGATCTTTTCGGTCGTGAGTTCGGAGACGTAGCCACCTACTCCCAACATCAGCCGGACTCCGATTACCTCGGGAACTTGCTCCGTAGTAAGACATTCATCGCGCTTGCTGCCTTCGACCAAGAAGCGGTTGTTGGCGCTCTCGCGGCTTACGTTCTGCCCAAGTTTGAGCAGCCGCGTAGTGAGATCTATATCTATGATCTCGCAGTCTCCGGAGAGCACCGGAGGCAGGGCATTGCCACCGCGCTCATCAATCTCCTCAAGCATGAGGCCAACGCGCTTGGTGCTTATGTGATCTACGTGCAAGCAGATTACGGTGACGATCCCGCAGTGGCTCTCTATACAAAGTTGGGCATACGGGAAGAAGTGATGCACTTTGATATCGACCCAAGTACCGCCACCTAACAATTCGTTCAAGCCGAGATCGGCTTCCCGGCCGCGGAGTTGTTCGGTAAATTGGACAACGGTCCGCGCGTTGTCCTTTTCCGCTGCATAACCCTGCTTCGGGGTCATTATAGCGATTTTTTCGGTATATCCATCCTTTTTCGCACGATATACAGGATTTTGCCAAAGGGTTCGTGTAGACTTTCCTTGGTGTATCCAACGGCGTCAGCCGGGCAGGATAGGTGAAGTAGGCCCACCCGCGAGCGGGTGTTCCTTCTTCACTGTCCCTTATTCGCACCTGGCGGTGCTCAACGGGAATCCTGCTCTGCGAGGCTGGCCGTAGGCCGGCCGATAATCTCATGACCAAAATCCCTTAACGTGAGTTTTCGTTCCACTGAGCGTCAGACCCCGTAGAAAAGATCAAAGGATCTTCTTGAGATCCTTTTTTTCTGCGCGTAATCTGCTGCTTGCAAACAAAAAAACCACCGCTACCAGCGGTGGTTTGTTTGCCGGATCAAGAGCTACCAACTCTTTTTCCGAAGGTAACTGGCTTCAGCAGAGCGCAGATACCAAATACTGTTCTTCTAGTGTAGCCGTAGTTAGGCCACCACTTCAAGAACTCTGTAGCACCGCCTACATACCTCGCTCTGCTAATCCTGTTACCAGTGGCTGCTGCCAGTGGCGATAAGTCGTGTCTTACCGGGTTGGACTCAAGACGATAGTTACCGGATAAGGCGCAGCGGTCGGGCTGAACGGGGGGTTCGTGCACACAGCCCAGCTTGGAGCGAACGACCTACACCGAACTGAGATACCTACAGCGTGAGCTATGAGAAAGCGCCACGCTTCCCGAAGGGAGAAAGGCGGACAGGCATCCGGTAAGCGGCAGGGTCGGAACAGGAGAGCGCACGAGGGAGCTTCCAGGGGGAAACGCCTGGTATCTTTATAGTCCTGTCGGGTTTCGCCACCTCTGACTTGAGCGTCGATTTTTGTGATGCTCGTCAGGGGGGCGGAGCCTATGGAAAAACGCCAGCAACGCGGCCGTGAAAGGCAGGCCGGTCCGTGGTGGCCACGGCCTCTAGGCCAGATCCAGCGGCATCTGGGTTAGTCGAGCGCGGGCCGCTTCCCATGTCTCACCAGGGCGAGCCTGTTTCGCGATCTCAGCATCTGAAATCTTCCCGGCCTTGCGCTTCGCTGGGGCCTTACCCACCGCCTTGGCGGGCTTCTTCGGTCCAAAACTGAACAACAGATGTGTGACCTTGCGCCCGGTCTTTCGCTGCGCCCACTCCACCTGTAGCGGGCTGTGCTCGTTGATCTGCGTCACGGCTGGATCAAGCACTCGCAACTTGAAGTCCTTGATCGAGGGATACCGGCCTTCCAGTTGAAACCACTTTCGCAGCTGGTCAATTTCTATTTCGCGCTGGCCGATGCTGTCCCATTGCATGAGCAGCTCGTAAAGCCTGATCGCGTGGGTGCTGTCCATCTTGGCCACGTCAGCCAAGGCGTATTTGGTGAACTGTTTGGTGAGTTCCGTCAGGTACGGCAGCATGTCTTTGGTGAACCTGAGTTCTACACGGCCCTCACCCTCCCGGTAGATGATTGTTTGCACCCAGCCGGTAATCATCACACTCGGTCTTTTCCCCTTGCCATTGGGCTCTTGGGTTAACCGGACTTCCCGCCGTTTCAGGCGCAGGGCCGCTTCTTTGAGCTGGTTGTAGGAAGATTCGATAGGGACACCCGCCATCGTCGCTATGTCCTCCGCCGTCACTGAATACATCACTTCATCGGTGACAGGCTCGCTCCTCTTCACCTGGCTAATACAGGCCAGAACGATCCGCTGTTCCTGAACACTGAGGCGATACGCGGCCTCGACCAGGGCATTGCTTTTGTAAACCATTGGGGGTGAGGCCACGTTCGACATTCCTTGTGTATAAGGGGACACTGTATCTGCGTCCCACAATACAACAAATCCGTCCCTTTACAACAACAAATCCGTCCCTTCTTAACAACAAATCCGTCCCTTAATGGCAACAAATCCGTCCCTTTTTAAACTCTACAGGCCACGGATTACGTGGCCTGTAGACGTCCTAAAAGGTTTAAAAGGGAAAAGGAAGAAAAGGGTGGAAACGCAAAAAACGCACCACTACGTGGCCCCGTTGGGGCCGCATTTGTGCCCCTGAAGGGGCGGGGGAGGCGTCTGGGCAATCCCCGTTTTACCAGTCCCCTATCGCCGCCTGAGAGGGCGCAGGAAGCGAGTAATCAGGGTATCGAGGCGGATTCACCCTTGGCGTCCAACCAGCGGCACCAGCGGCGCCTGAGAGGGGCGCGCCCAGCTGTCTAGGGCGGCGGATTTGTCCTACTCAGGAGAGCGTTCACCGACAAACAACAGATAAAACGAAAGGCCCAGTCTTTCGACTGAGCCTTTCGTTTTATTTGATGCCT

**DNA sequence of UGI**

ATGTCTGGTGGTTCTACTAATCTGTCAGATATTATTGAAAAGGAAACCGGTAAGCAACTGGTTATCCAGGAATCCATCCTCATGCTCCCAGAGGAGGTGGAAGAAGTCATTGGGAACAAGCCGGAAAGCGATATACTCGTGCACACCGCCTACGACGAGAGCACCGACGAGAATGTCATGCTTCTGACTAGCGACGCCCCTGAATACAAGCCTTGGGCTCTGGTCATACAGGATAGCAACGGTGAGAACAAGATTAAGATGCTCTCTGGTGGTTCTTGA

**DNA sequence of XTEN linker**

GATAGCGGCGGCTCGAGCGGCGGCAGTAGCGGCAGCGAAACCCCGGGCACCTCGGAGTCGGCCACCCCGGAGAGCTCGGGTGGAAGCTCGGGTGGCTCC

**DNA sequence of SacB**

ATGAACATCAAAAAGTTTGCAAAACAAGCAACAGTATTAACCTTTACTACCGCACTGCTGGCAGGAGGCGCAACTCAAGCGTTTGCGAAAGAAACGAACCAAAAGCCATATAAGGAAACATACGGCATTTCCCATATTACACGCCATGATATGCTGCAAATCCCTGAACAGCAAAAAAATGAAAAATATCAAGTTCCTGAATTCGATTCGTCCACAATTAAAAATATCTCTTCTGCAAAAGGCCTGGACGTTTGGGACAGCTGGCCATTACAAAACGCTGACGGCACTGTCGCAAACTATCACGGCTACCACATCGTCTTTGCATTAGCCGGAGATCCTAAAAATGCGGATGACACATCGATTTACATGTTCTATCAAAAAGTCGGCGAAACTTCTATTGACAGCTGGAAAAACGCTGGCCGCGTCTTTAAAGACAGCGACAAATTCGATGCAAATGATTCTATCCTAAAAGACCAAACACAAGAATGGTCAGGTTCAGCCACATTTACATCTGACGGAAAAATCCGTTTATTCTACACTGATTTCTCCGGTAAACATTACGGCAAACAAACACTGACAACTGCACAAGTTAACGTATCAGCATCAGACAGCTCTTTGAACATCAACGGTGTAGAGGATTATAAATCAATCTTTGACGGTGACGGAAAAACGTATCAAAATGTACAGCAGTTCATCGATGAAGGCAACTACAGCTCAGGCGACAACCATACGCTGAGAGATCCTCACTACGTAGAAGATAAAGGCCACAAATACTTAGTATTTGAAGCAAACACTGGAACTGAAGATGGCTACCAAGGCGAAGAATCTTTATTTAACAAAGCATACTATGGCAAAAGCACATCATTCTTCCGTCAAGAAAGTCAAAAACTTCTGCAAAGCGATAAAAAACGCACGGCTGAGTTAGCAAACGGCGCTCTCGGTATGATTGAGCTAAACGATGATTACACACTGAAAAAAGTGATGAAACCGCTGATTGCATCTAACACAGTAACAGATGAAATTGAACGCGCGAACGTCTTTAAAATGAACGGCAAATGGTACCTGTTCACTGACTCCCGCGGATCAAAAATGACGATTGACGGCATTACGTCTAACGATATTTACATGCTTGGTTATGTTTCTAATTCTTTAACTGGCCCATACAAGCCGCTGAACAAAACTGGCCTTGTGTTAAAAATGGATCTTGATCCTAACGATGTAACCTTTACTTACTCACACTTCGCTGTACCTCAAGCGAAAGGAAACAATGTCGTGATTACAAGCTATATGACAAACAGAGGATTCTACGCAGACAAACAATCAACGTTTGCGCCAAGCTTCCTGCTGAACATCAAAGGCAAGAAAACATCTGTTGTCAAAGACAGCATCCTTGAACAAGGACAATTAACAGTTAACAAATAA

**DNA sequence of the pykA gene in *P.putida* KT2440**

ATGAGCATCCGCCGCACCAAAATCGTCGCCACCCTTGGCCCCGCCAGCAACTCGCCGGAAGTGATCGAACAACTGATCCTCGCCGGCCTGGACGTGGCACGTCTGAACTTCTCCCACGGCACGCCGGACGAGCACAAGGCCCGCGCGCGCCTGATCCGTGACATCGCCGCCAAGAATGGCCGCCATGTTGCACTGCTGGGCGACCTGCAGGGTCCGAAGATCCGCATCGCCAAGTTCGCCAACAAGCGCATCGAACTGAAGATCGGTGACAAGTTCACCTTCTCCACCGCCCACCCGCTGACCGAAGGCAACCAGGACATCGTCGGTATCGACTACCCCGACCTGGTCAAGGACTGCGGCGTTGGTGACGAACTGCTGCTCGACGATGGCCGCGTGGTCATGCGCGTCGAAACCGCCACTGCAGATGCCCTGCACTGCGTGGTGATCATCGGTGGCCCACTGTCGGACCACAAAGGCATCAACCGTAAAGGTGGCGGCCTGACCGCACCGGCCCTGACCGAAAAAGACAAGGCCGACATCAAACTGGCTGCGGAAATGGACCTGGACTACCTGGCCGTATCCTTCCCGCGTGACGCCAGCGATATGGAATACGCGCGCAAGCTGCGTGACGAAGCCGGCGGCAGCGCCTGGCTGGTAGCCAAGATCGAACGCGCCGAAGCGGTGGCCGATGACGAGACCCTCGACAAGCTGATCGCCGCCTCCGACGCTGTGATGGTTGCCCGTGGTGACCTGGGCGTGGAAATCGGCGACGCCGAGCTGATCGCTATCCAGAAGAAGATCATCCAGCACGCCCGCCGCAACAACAAGGCCGTGATCGTGGCGACCCAGATGATGGAGTCGATGATCCAGAACCCGATGCCGACCCGCGCGGAAGTGTCCGACGTGGCCAACGCCGTGCTGGACAACACCGATGCGGTGATGCTGTCGGCCGAAAGCGCCGCCGGTTCCTACCCGATCGAAGCTGTCCAGGCCATGGCACGCATCTGCCTGGGTGCCGAAAAGCACCCGACCAGCCAGAAGTCCAGCCATCGCCTGCACACCACCTTCCAGCGCTGCGACGAAAGCATCGCCTTGGCGGCCATGTACACTGCCAACCACTTCCCGGGCGTAAAGGCGATCATCGCCCTGACCGAAAGCGGCTACACCCCGCTGATCATGTCGCGCCTGCGTTCGCATGTACCGATCTTCGCCCTGTCGCCGCACCGCGCCACCCAGGCGCGCGCCTCGATGTTCCGTGGCGTGTACCCGATTGCCTTCGACCCGGCCGCGCTGCCGGCCGATAAGGTGAGCCAGGCGGCGGTCGACGAACTGCTCAAACGTGGCCTGGTGGAGCAAGGTGACTGGGTGATCCTGACCAAGGGTGACAGCTACCACACCATCGGTGGCACCAATGGCATGAAGATCCTGCACGTCGGTGATCCGCTGGTCGGTTGA
